# Supplementary material for: Convergent approach for direct cross-coupling enabled by flash irreversible generation of cationic and anionic species
Source: Nat Commun. 2024 Jun 13;15:4873. doi: 10.1038/s41467-024-48723-1 (PMC11176188; doi:10.1038/s41467-024-48723-1)
Supplement: Supplementary file 1 — Supplementary Information [file 41467_2024_48723_MOESM1_ESM.pdf]

Supplementary information

Convergent approach for direct cross-coupling enabled by flash irreversible generation of cationic and anionic species

Hiroki Soutome[1,2], Hiroki Yamashita[1], Yutaka Shimizu[1], Masahiro Takumi[1], Yosuke Ashikari[1], and Aiichiro Nagaki[1,*]

[1] Department of Chemistry, Graduate School of Science, Hokkaido University, kita-10 nishi-8, kita-ku, Sapporo, Hokkaido 060-0810 Japan

[2] Yokohama Technical Center, AGC Inc. 1-1 Suehiro-cho, Tsurumi-ku, Yokohama, Kanagawa 230-0045, Japan

*Corresponding author: nagaki@sci.hokudai.ac.jp

1. General remarks
2. Synthetic procedures
    2.1 Generation and reactions of *N*-acyliminium ion in a batch reactor (Procedure A–D)
    2.2 Effect of flow rate and mixer for generation and reaction of *N*-acyliminium ion 2a in a flow microreactor (Method E)
    2.3 Mass transfer characterization
    2.4 Reactions of enamines with TfOH followed by reaction with allyltrimethylsilane with varying residence time and temperature
    2.5 Reactions of enamines with other acids
    2.6 Generation of carbocationic species and reactions with nucleophiles
    2.7 Low-temperature NMR analysis of carbocations
    2.8 Reaction of carbocationic species with sp^2-carbanions
    2.9 Reaction of carbocationic species with sp-carbanions
    2.10 Twice direct cross-coupling reaction
3. ^{1}H, ^{13}C and ^{19}F NMR spectra
4. References

1. General Information

Abbreviations. atmospheric pressure chemical ionization (APCI), approximately (approx. or ~), broad (br), butyl (Bu), degrees Celsius (°C), calculated (calcd), deuteritated chloroform ($CDCl_3$), deuteritated dichloromethane (CD_2Cl_2), dichloromethane (CH_2Cl_2), centimeter(s) (cm), doublet (d), Damköhler number (Da), electron ionization (EI), equivalent (equiv or eq), electrospray ionization (ESI), ethyl acetate (EtOAc), gram(s) (g), gas chromatography (GC), gel permeation chromatography (GPC), hour(s) (h), high resolution mass-spectrometry (HRMS), hertz (Hz), coupling constant (*J*), length of tubes (L), liter(s) (L), mol L^{-1} of molar concentration (M), multiplet (m), methyl (Me), metallic functionality (*Met*), milligram(s) (mg), megahertz (MHz), minute(s) (min), milliliter(s) (mL), millimole(s) (mmol), mole(s) (mol), normal (*n*), nuclear magnetic resonance (NMR), parts per million (ppm), polytetrafluoro-ethylene or Teflon (PTFE), quartet (q), Reynold's number (Re), room temperature (25±3 °C, rt), second(s) (s or sec), singlet (s), triplet (t), tertiary (*t* or *tert*), tri-*n*-butylammonium fluoride (TBAF), trifluoromethanesulfonic acid (TfOH), tetrahydrofuran (THF), tetramethylsilane (TMS), residence time of microtube reactor R_n (t^{Rn}), chemical shift in ppm downfield from TMS (δ), inner diameter of tubes and mixers (ϕ), micrometer(s) (μm).

General. ^{1}H, ^{13}C and ^{19}F NMR spectra were recorded in on Varian MERCURY plus-400 (^{1}H 400 MHz, ^{13}C 100 MHz), JEOL JNM-ECZ400S (^{1}H 400 MHz, ^{13}C 100 MHz, ^{19}F 376 MHz), or JEOL JNM-ECZ-500R spectrometer (^{1}H 500 MHz). Chemical shifts are recorded using a solvent ($CHCl_3$: 7.26 ppm, CH_2Cl_2: 5.32 ppm) signal as an internal standard for ^{1}H NMR, methin signal of $CHCl_3$ for ^{13}C NMR (77.36 ppm) unless otherwise noted. No internal standard for chemical shifts was used for ^{19}F NMR analyses. Because of rotemers, the ^{1}H and ^{13}C NMR charts of carbamates showed broad peaks. GC analysis was performed on a SHIMADZU GC-2014 gas chromatograph equipped with a flame ionization detector using a fused silica capillary column (column, CBP1; 0.22 mm x 25 m). Temperature of GC oven was 50 °C at first, and after 5min the temperature was increased 10 °C per min. GC yields were calculated by GC analyses with internal standards such as *n*-tetradecane using calibration lines derived from commercial or isolated compounds with the internal standards. UV/Vis measurement was performed on JASCO V730 with a disposable cuvette made of PMMA (manufactured by JASCO Co., optical path length 1.0 cm). Mass spectra were obtained on Thermo Fisher Scientific EXACTIVE plus (ESI and APCI), JEOL JMS-T100CS (ESI), JEOL JMSSX102A (EI), and JEOL JMS-700 (EI). Merck pre-coated silica gel F254 plates (thickness 0.25 mm) were used for TLC analyses. Flash chromatography was carried out on a silica gel (Kanto Chem. Co., Silica Gel N, spherical, neutral, 40–100 μm). Preparative GPC was carried out on Japan Analytical Industry LC-918 equipped with JAIGEL-1H and 2H using $CHCl_3$ as an eluent. All batch reactions were carried out in a flame-dried glassware under argon atmosphere unless otherwise noted.

Flow Synthesis. Stainless steel (SUS304) T- and V-shaped micromixers with inner diameter of 250 and 500 μm were manufactured by Sanko Seiki Co., Inc. Stainless steel (SUS316) microtube reactors with 1000, 500, and 250 μm inner diameter and PTFE tube with inner diameter of 1000 μm were purchased from GLSciences. The syringe pumps (Harvard Model PHD ULTRA) equipped with gastight syringes (purchased from SGE) were used for introduction of the solutions into the micromixer systems via stainless steel fittings (GL Sciences, 1/16 OUW). Flow microreactor system is composed with stainless steel pre-cooling units (**P1**, **P2**, etc.), stainless steel microtube reactors (**R1**, **R2**, etc.), T- or V-shaped micromixers (**M1**, **M2**, etc.), and, if necessary, PTFE tube with inner diameter of 1000 μm. Unless otherwise noted, the inner diameter of the stainless and PTFE tubes is 1000 μm, and the length of the pre-cooling units is 100 cm. Unless otherwise noted, the inner diameter of micromixers is 250 μm. The solution of *n*-butyllithium was prepared by dilution of the commercial solution with dehydrated *n*-hexane.

Materials. Dehydrated THF, diethyl ether, dichloromethane and *n*-hexane were purchased from FUJIFILM Wako Pure Chemical Corporation and Kanto Chemical Co., Inc., and were used without further purification. A solution of *n*-butyllithium (in *n*-hexane, 1.6 M) was purchased from Kanto Chemical Co., Inc. and stored at −20 °C. CD_2Cl_2 was purchased from FUJIFILM Wako Pure Chemical Corporation, and dried over molecular sieves 4A before use. 2-Pyrrolidone, 2-piperidone, TfOH, allyltrimethylsilane, 1-trimethylsiloxy-1-cyclohexene, 1-methoxy-1-trimethylsilyloxypropene, 2-trimethylsiloxypropene, 1-phenyl-1-trimethylsiloxyethylene, 3,4-dihydro-2*H*-pyran (**1c**), 4-bromobenzotrifluoride, 1-bromo-4-fluorobenzene, 4-bromobenzonitrile, 1,4-dibromobenzene, 4-bromoanisole, 1-bromo-3-methoxybenzene, 1-bromo-2-methoxybenzene, 1-hexyne, methyl propiolate, glycidyl propargyl ether, 4-ethynylbenzonitrile, 3-ethynylthiophene, trimethylsilylacetylene, phenylacetylene, *n*-butylmagnesium chloride, and TBAF (in THF, 1.0 M) were purchased from commercial suppliers, and were used without further purification. Methyl 2,3-dihydropyrrole-1-carboxylate (**1a**),[1] methyl 3,4-dihydropyridine-2*H*-carboxylate (**1b**),[2] *tert*-butyl 2,3-dihydropyrrole-1-carboxylate (**1d**),[3] and allyl 2,3-dihydropyrrole-1-carboxylate (**1e**)[4] were prepared by protection of 2-pyrrolidone and 2-poperidone,[5] followed by DIBAL reduction and acidic β-elimination.[6] *n*-Butylzinc chloride was synthesized[7] and titrated[8] according to the literature.

2. Synthetic procedures

2.1 Generation and reaction of *N*-acyliminium ion 2a in a batch reactor (Method A–D)

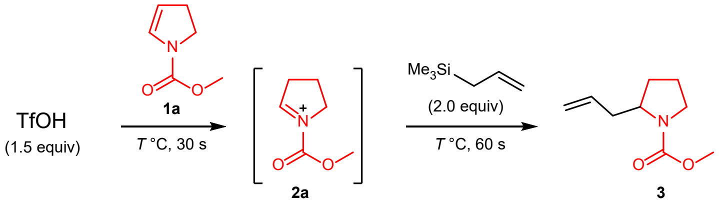

Method A. To a CH_2Cl_2 solution (6.0 mL) of TfOH (0.30 mmol) cooled at *T* °C, a solution of **1a** (0.200 mmol, 4.0 mL) was added for 30 s by hand. After completion of the addition, a CH_2Cl_2 solution of allyltrimethylsilane (0.40 mmol) was added immediately. The reaction mixture was stirred for 60 s, and a solution of TBAF (1.0 M in THF, 0.30 mL) was added for quenching. To the mixture, Et_3N (2.0 mL), brine (2.0 mL) and internal standard were added for GC analyses. The yields are summarized in Supplementary Table 1.

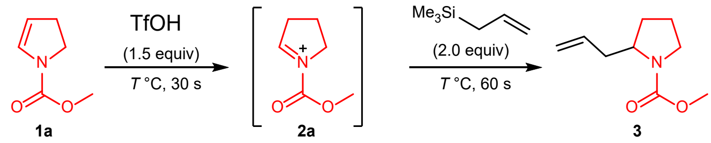

Method B. To a CH_2Cl_2 solution (4.0 mL) of **1a** (0.200 mmol) cooled at *T* °C, a CH_2Cl_2 solution (6.0 mL) of TfOH (0.30 mmol) was added for 30 s by hand. After completion of the addition, a CH_2Cl_2 solution (2.0 mL) of allyltrimethylsilane (0.40 mmol) was added immediately. The reaction mixture was stirred for 60 s, and a solution of TBAF (1.0 M in THF, 0.30 ml) was added for quenching. To the mixture, Et_3N (2.0 mL), brine (2.0 mL) and internal standard were added for GC analyses. The yields are summarized in Supplementary Table 1.

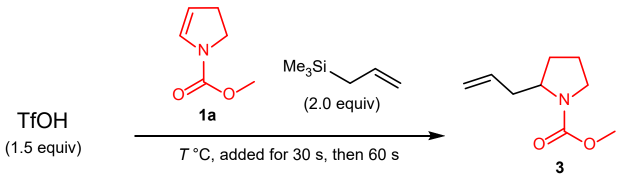

Method C. To a CH_2Cl_2 solution (6.0 mL) of TfOH (0.30 mmol) cooled at *T* °C, a CH_2Cl_2 solution (4 mL) of **1a** (0.200 mmol) and allyltrimethylsilane (0.40 mmol) was added for 30 s by hand. The reaction mixture was stirred for 60 s, and a solution of TBAF (1.0 M in THF, 0.30 mL) was added for quenching. To the mixture, Et_3N (2.0 mL), brine (2.0 mL) and internal standard were added for GC analyses. The yields are summarized in Supplementary Table 1.

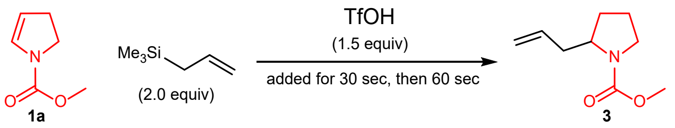

Method D. To a CH_2Cl_2 solution (4.0 mL) of **1a** (0.200 mmol) and allyltrimethylsilane (0.40 mmol), a CH_2Cl_2 solution (6.0 mL) of TfOH (0.30 mmol) was added for 30 s by hand. The reaction mixture was stirred for 60 s, and a solution of TBAF (1.0 M in THF, 0.30 mL) was added for quenching. To the mixture, Et_3N (2.0 mL), brine (2.0 mL) and internal standard were added for GC analyses. The yields are summarized in Supplementary Table 1.

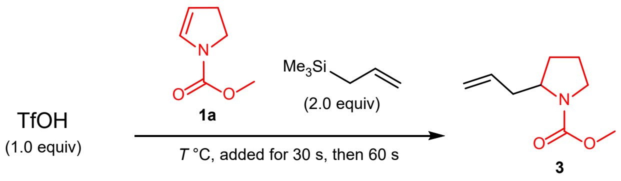

Method C' (TfOH 1 eq). To a CH_2Cl_2 solution (6.0 mL) of TfOH (0.20 mmol) cooled at *T* °C, a CH_2Cl_2 solution (4 mL) of **1a** (0.200 mmol) and allyltrimethylsilane (0.40 mmol) was added for 30 s by hand. The reaction mixture was stirred for 60 s, and a solution of TBAF (1.0 M in THF, 0.30 mL) was added for quenching. To the mixture, Et_3N (2.0 mL), brine (2.0 mL) and internal standard were added for GC analyses. The yields are summarized in Supplementary Table 1.

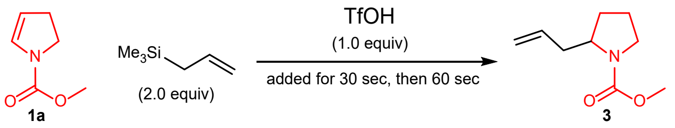

Method D' (TfOH 1 eq). To a CH_2Cl_2 solution (4.0 mL) of **1a** (0.200 mmol) and allyltrimethylsilane (0.40 mmol), a CH_2Cl_2 solution (6.0 mL) of TfOH (0.20 mmol) was added for 30 s by hand. The reaction mixture was stirred for 60 s, and a solution of TBAF (1.0 M in THF, 0.30 mL) was added for quenching. To the mixture, Et_3N (2.0 mL), brine (2.0 mL) and internal standard were added for GC analyses. The yields are summarized in Supplementary Table 1.

Supplementary Table 1. Results of batch reactions[a]

Method	TfOH (eq)	*T* (°C)	conversion (%)	yield of **3** (%)
A	1.5	0	100	3
A	1.5	−20	100	28
A	1.5	−40	100	29
A	1.5	−78	100	35
B	1.5	0	100	0
B	1.5	−20	100	9
B	1.5	−40	100	0
B	1.5	−78	100	12
C	1.5	0	100	44
C	1.5	−20	100	39
C	1.5	−40	100	45
C	1.5	−78	100	35
C′	1.0	0	100	7
C′	1.0	−20	100	7
C′	1.0	−40	100	8
C′	1.0	−78	100	10
D	1.5	0	100	21
D	1.5	−20	100	21
D	1.5	−40	100	18
D	1.5	−78	100	20
D′	1.0	0	100	2
D′	1.0	−20	100	3
D′	1.0	−40	100	2
D′	1.0	−78	100	0

[a]Yields and conversions were determined by GC using an internal standard. Retention time of **3**: 15.7 min

Isolation and quantification of the dimer

After extraction, the crude mixture was purified by flash chromatography (hexane/EtOAc = 4/1) and GPC to afford the dimer (**4**). The yield of is summarized in Supplementary Table 2.

Supplementary Table 2. Yields of dimer **4**[a]

condition	yield (%)	condition	yield (%)
Method A, 0 °C	64	Method B, 0 °C	64
Method A, −20 °C	54	Method B, −20 °C	62
Method A, −40 °C	58	Method B, −40 °C	64
Method A, −78 °C	66	Method B, −78 °C	48
Method C, 0 °C	50	Method D, 0 °C	42
Method C, −20 °C	48	Method D, −20 °C	44
Method C, −40 °C	48	Method D, −40 °C	42
Method C, −78 °C	48	Method D, −78 °C	38
Method C′, 0 °C	78	Method D′, 0 °C	74
Method C′, −20 °C	64	Method D′, −20 °C	60
Method C′, −40 °C	72	Method D′, −40 °C	76
Method C′, −78 °C	80	Method D′, −78 °C	42
Flow, 2.5 mL/min	40	Flow, 15 mL/min	32
Flow, 5.0 mL/min	30	Flow, 20 mL/min	26
Flow, 10 mL/min	28		

[a]Yields were determined by GC. Flow reactions were carried out using V-250 mixer at 0 °C.

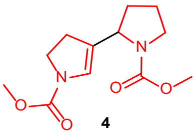
4

Methyl 4-(*N*-methoxycarbonylpyrrolidine-2-yl)-2,3-dihydropyrrole-1-carboxylate (4).

GC retention time: 25.4 min. ^{1}H NMR (400 MHz, $CDCl_3$, rotamer) δ 1.70–1.96 (m, 4 H), 2.40–2.68 (br, 2 H), 3.28–3.48 (br, 2 H), 3.64 (s, 3 H), 3.67 (s, 3 H), 3.69–3.82 (m, 2 H), 4.34-4.50 (br, 1 H), 6.17–6.39 (br, 1 H); ^{13}C NMR (100 MHz, $CDCl_3$, rotamer) δ 23.4 and 24.0, 29.1, 30.3 and 31.1, 45.9 and 46.1, 46.6, 52.6, 52.7, 55.7 and 56.2, 123.91, 124.7 and 125.3, 152.9 and 153.6, 155.9; HRMS (ESI) calcd for $C_{12}H_{18}N_2O_4Na$ $[M+Na]^+$: 277.1159, found: 277.1152.

2.2 Effect of flow rate and mixer for generation and reaction of *N*-acyliminium ion 2a in a flow microreactor (Method E)

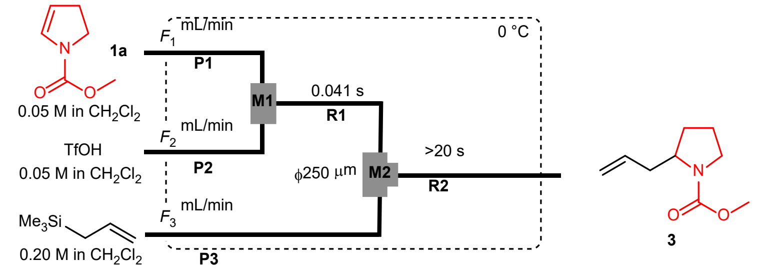

A flow microreactor system consisting of a micromixer (**M1**, V-shaped or T-shaped, ϕ = 250, or 500 μm) and a T-shaped micromixer (**M2**, ϕ = 250 μm), two microtube reactors (**R1** and **R2**), and three pre-cooling units (**P1**–**P3**) was used. The flow microreactor system was dipped in a cooling bath (0 °C). A solution of **1a** (0.0500 M in CH_2Cl_2, flow rate: F_1 mL/min) and a solution of TfOH (0.050 M in CH_2Cl_2, flow rate: F_2 mL/min) were introduced into **M1** using syringe pumps. The mixed solution was passed through **R1** (ϕ^{R1} μm, L^{R1} cm, 0.041 s), and was mixed with a solution of allyltrimethylsilane (0.20 M in CH_2Cl_2, flow rate: F_3 mL/min) in **M2.** The resulting solution was passed through **R2** (L^{R2}= 1000 cm). After a steady state was reached, an aliquot of the product solution was collected and was treated with TBAF, Et_3N and brine. The reaction mixture was analyzed by GC. The results are summarized in Supplementary Table 3.

Supplementary Table 3. Flow rate and mixer dependency of the generation and reaction of **2a**[a]

entry	F_1 (mL/min)	F_2 (mL/min)	F_3 (mL/min)	mixer **M1** shape	mixer **M1** φ^{M1} (μm)	tube **R1** φ^{R1} (μm)	tube **R1** L^{R1} (cm)	yield of **3** (%)
1	8	12	4	V	250	500	7.0	65
2	6	9	3	V	250	500	5.25	65
3	4	6	2	V	250	500	3.5	67
4	2	3	1	V	250	250	7.0	46
5	1	1.5	0.5	V	250	250	3.5	32
6	8	12	4	T	250	500	7.0	51
7	8	12	4	T	500	500	7.0	27

[a]Yields were determined by GC using an internal standard. Retention time: 15.7 min

2.3 Mass transfer characterization

Villermaux–Dushman protocol for characterization of micromixers (different flow rate)

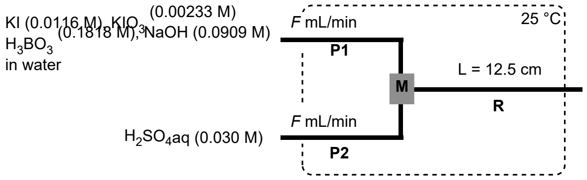

The set of solution concentration was based on the reported condition.[9] A flow microreactor system consisting of micromixer **M** (V- or T-shaped, ϕ = 250 or 500 μm), microtube reactor **R**, and two pre-cooling units (**P1** and **P2**) was used. The flow microreactor system was dipped in a water bath (25 °C). An aqueous solution containing KI (0.0116 M), KIO_3 (0.00233 M), H_3BO_3 (0.1818 M) and NaOH (0.0909 M) was introduced in **M** using a syringe pump, whereas an aqueous solution of H_2SO_4 (0.030 M) was also introduced into **M**. The flow rates of those solutions are the same (*F* mL/min). The solution mixed in **M** was passed through **R** (L = 12.5 cm). After a steady state was reached, an aliquot of the emitting solution was collected and its absorbance at 353 nm was recorded. According to the literature,[10] the mixing time of micromixers can be calculated as below;

$$t_m = 0.33 \times Abs \times [\mathrm{H}^+]^{-4.55} \times [\mathrm{KI}]^{-1.5} \times [\mathrm{KIO_3}]^{5.8} \times [\mathrm{NaOH}]^{-2} \times [\mathrm{H_3BO_3}]^{-2}$$

where, t_m is mixing time (second), *Abs* is the absorbance at 353 nm of the mixture, and [*chemical*] is the concentration of the chemical before being mixed. The results of the Villermaux–Dushman protocol is summarized in Supplementary Table 4. The small differences observed in the results without that of 2.5 mL/min are attributed to having reached the limit of detection. Since dichloromethane (the reaction solvent) has lower viscosity and higher density than water, the net mixing time should be shorter than this table. The relation of the mixing time with the yields for the reaction of iminium ion **2a** with allyltrimethylsilane is summarized in Supplementary Figure 1.

Supplementary Figure 1. Yields and mixing time of micromixers[a]

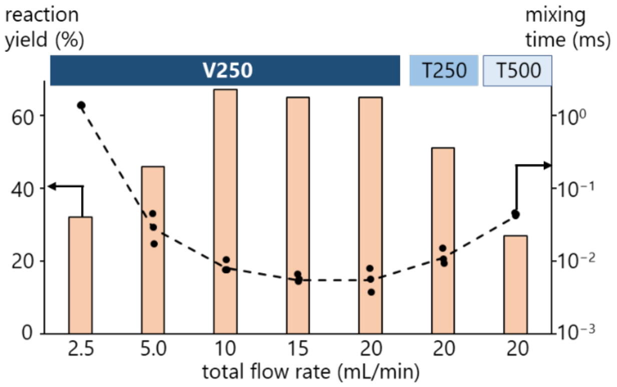

[a]Bars indicate yield of **3**. Dots indicate mixing time. Total flow rate means the flow rate introducing to the mixer. Thus, the sum of F_1 and F_2 for reaction yield.

Supplementary Table 4. Villermaux–Dushman protocol for micromixers[a]

total flow rate (mL/min)	micromixer[b]	absorbance at 353 nm	t_m (ms)	average of t_m (ms)
20	V-250	0.008	0.0035	
		0.012	0.0053	0.0054
		0.017	0.0075	
15	V-250	0.012	0.0053	
		0011	0.0049	0.0054
		0.014	0.0062	
10	V-250	0.016	0.0071	
		0.022	0.0097	0.0080
		0.016	0.0071	
5.0	V-250	0.096	0.042	
		0.060	0.027	0.028
		0.037	0.016	
2.5	V-250	2.90	1.28	
		2.96	1.31	1.29
		2.89	1.27	
20	T-250	0.031	0.014	
		0.023	0.010	0.011
		0.020	0.0088	
20	T-500	0.097	0.043	
		0.088	0.039	0.041
		0.092	0.041	

[a]Total flow rate means sum of the velocity of the solutions introducing micromixer M. Thus, it can be described as 2×*F*. [b]Alphabet means its shape (V-shape or T-shape), and the number means the inner diameter (μm).

Reynold's Number

The Reynold's number can be calculated according to the following equation;

$$Re = \frac{d \times v \times D_H}{\mu}$$

where, d is density (g/mL, dichloromethane at 0 °C: 1.363),[11] v is velocity (m/s), D_H is hydraulic diameter (m), and μ is viscosity (Pa·s, dichloromethane at 0 °C: 0.5328).[11] Thus, with the best

condition (total flow rate: 20 mL/min, temperature: 0 °C, and tube diameter: 500 µm, *vide infra*) can be calculated as 2200, which indicates the condition is in a transition to a turbulent flow regime.

Damköhler Number

The Damköhler number can be defined as follows;[12]

$$Da = \frac{reaction\ rate}{mixing\ rate}$$

$$\sim \frac{mixing\ time\ scale}{reaction\ time\ scale}$$

Although the value should have a margin of error, the scale of both the reaction time and the mixing time of the cation generation reaction can be estimated. Since Supplementary Table 4 (*vide infra*) indicates the reaction at 0 °C reached the highest yield with 82 ms of the residence time, reaction time is in 10 ms scale. Whereas, above-mentioned Villermaux–Dushman protocol indicates the mixing time for high-yielding condition (total flow rate is higher than 2.5 mL/min) must be smaller than 1 ms. Thus, Da can be determined as smaller than 0.1, which is much smaller than 1.

2.4 Reactions of enamines with TfOH followed by reaction with allyltrimethylsilane with varying residence time and temperature

Reactions of 1a (General procedure for making the contour map)

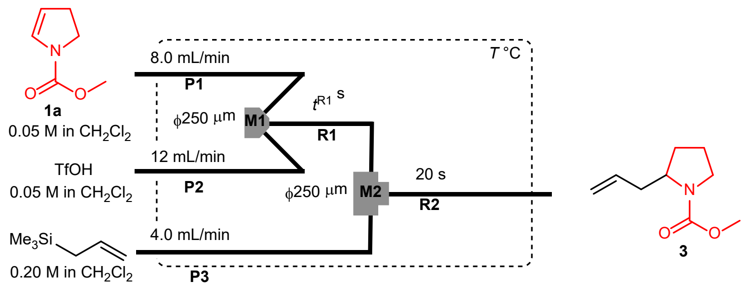

A flow microreactor system consisting of a V-shaped micromixer (**M1**, ϕ = 250 µm) and a T-shaped micromixer (**M2**, ϕ = 250 µm), two microtube reactors (**R1** and **R2**), and three pre-cooling units (**P1**–**P3**) was used. The flow microreactor system was dipped in a cooling bath (*T* °C). A solution of **1a** (0.0500 M in CH_2Cl_2, flow rate: 8.0 mL/min) and a solution of TfOH (0.050 M in CH_2Cl_2, flow rate: 12.0 mL/min) were introduced into **M1** using syringe pumps. The mixed solution was passed through **R1** (ϕ^{R1} µm, L^{R1} cm, t^{R1} s), and was mixed with a solution of allyltrimethylsilane (0.20 M in CH_2Cl_2,

flow rate: 4.0 mL/min) in **M2.** The resulting solution was passed through **R2** (L^{R2}= 1000 cm, t^{R2} = 20 s). After a steady state was reached, an aliquot of the reacting solution was collected and was treated with TBAF, Et_3N and brine. The yield of **3** was analyzed by GC, and was summarized in Supplementary Table 5.

Supplementary Table 5. Yields of **3** with varying temperature (*T*) and residence time in **R1** (t^{R1})[a]

T (°C)	φ^{R1} (μm)	L^{R1} (cm)	t^{R1} (s)	yield of **3** (%)
30	500	3.5	0.021	61
	1000	3.5	0.082	55
	1000	25	0.59	43
	1000	150	3.5	18
	1000	300	7.1	13
15	500	3.5	0.021	78
	1000	3.5	0.082	65
	1000	25	0.59	46
	1000	150	3.5	31
	1000	300	7.1	25
0	500	3.5	0.021	64
	1000	3.5	0.082	72
	1000	25	0.59	62
	1000	150	3.5	42
	1000	300	7.1	32
-15	500	3.5	0.021	63
	1000	3.5	0.082	75
	1000	25	0.59	58
	1000	150	3.5	52
	1000	300	7.1	42

[a]Yields were determined by GC using an internal standard. Retention time: 15.7 min

Supplementary Table 6. Recovery of **1a** and yield of dimer **4** with varying temperature (T) and residence time in **R1** (t^{R1})[a]

T (°C)	φ^{R1} (μm)	L^{R1} (cm)	t^{R1} (s)	recovery of **1a** (%)	yield of dimer **4** (%)
45	500	3.5	0.021	0	27
30	500	3.5	0.021	0	24
15	500	3.5	0.021	0	23
	1000	25	0.59	0	24
	1000	150	3.5	0	42
	1000	300	7.1	0	44

[a]Yields were determined by GC using an internal standard. Retention time of **1a**: 15.7 min. Retention time of **4**: 25.4 min.

Reactions of 1b

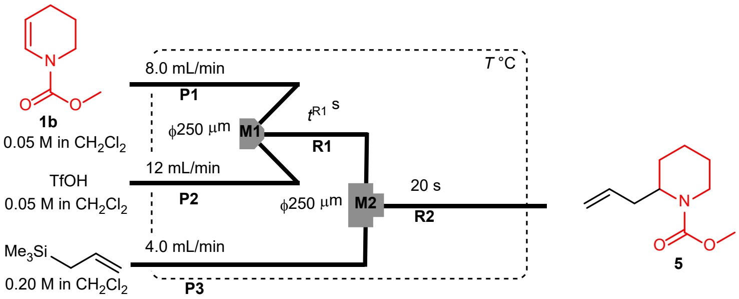

The similar flow microreactor system for **1a** using a solution of **1b** (0.0500 M in CH_2Cl_2, flow rate: 8.0 mL/min), TfOH (0.050 M in CH_2Cl_2, flow rate: 12.0 mL/min), and allyltrimethylsilane (0.20 M in CH_2Cl_2, flow rate: F_3 mL/min) was used. The yield of desired product **5** (methyl 2-allylpiperidine-1-carboxylate) was analyzed by GC. The results are summarized in Supplementary Table 7.

Supplementary Table 7. Yields of **5** with varying temperature (*T*) and residence time t^{R1} [a]

T (°C)	φ^{R1} (μm)	L^{R1} (cm)	t^{R1} (s)	yield of **5** (%)
30	500	3.5	0.021	29
	1000	3.5	0.082	28
	1000	25	0.59	20
	1000	150	3.5	10
	1000	300	7.1	7
15	500	3.5	0.021	46
	1000	3.5	0.082	43
	1000	25	0.59	36
	1000	150	3.5	24
	1000	300	7.1	20
0	500	3.5	0.021	53
	1000	3.5	0.082	50
	1000	25	0.59	43
	1000	150	3.5	35
	1000	300	7.1	27
-15	500	3.5	0.021	68
	1000	3.5	0.082	72
	1000	25	0.59	62
	1000	150	3.5	51
	1000	300	7.1	43

[a]Yields were determined by GC using an internal standard. Retention time: 15.7 min

2.5 Reactions of enamines with other acids

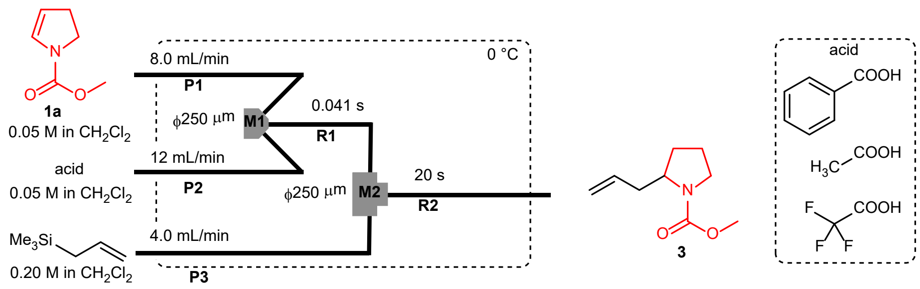

A flow microreactor system consisting of a V-shaped micromixer (**M1**, ϕ = 250 μm) and a T-shaped micromixer (**M2**, ϕ = 250 μm), two microtube reactors (**R1** and **R2**), and three pre-cooling units (**P1**–**P3**) was used. The flow microreactor system was dipped in a cooling bath (0 °C). A solution of **1a** (0.0500 M in CH_2Cl_2, flow rate: 8.0 mL/min) and a solution of acid (0.050 M in CH_2Cl_2, flow rate: 12.0 mL/min) were introduced into **M1** using syringe pumps. The mixed solution was passed through **R1** (ϕ = 500 μm, L^{R1} cm, t^{R1} s), and was mixed with a solution of allyltrimethylsilane (0.20 M in CH_2Cl_2, flow rate: 4.0 mL/min) in **M2.** The resulting solution was passed through **R2** (L^{R2}= 1000 cm, t^{R2} = 20 s). After a steady state was reached, an aliquot of the reacting solution was collected and was treated with TBAF, Et_3N and brine. The reaction was analyzed by GC, revealing that the reactions using benzoic acid, acetic acid, and trifluoroacetic acid did not afford desired product **3**.

2.6 Generation of carbocationic species and reactions with nucleophiles

Generation and reactions of *N*-acyliminium ions with neutral nucleophiles

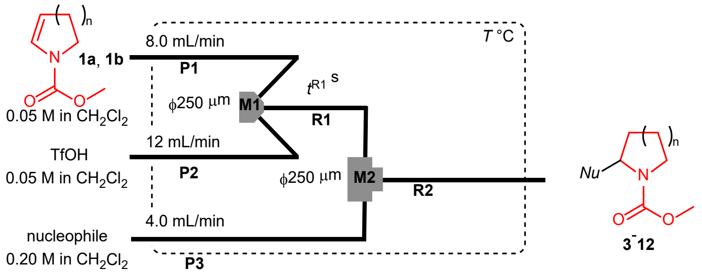

A flow microreactor system consisting of a V-shaped micromixer (**M1**, ϕ = 250 μm) and a T-shaped micromixer (**M2**, ϕ = 250 μm), two microtube reactors (**R1** and **R2**), and three pre-cooling units (**P1**–**P3**) was used. The flow microreactor system was dipped in a cooling bath (T = 15 °C). A solution of the cation precursor (0.0500 M in CH_2Cl_2, flow rate: 8.0 mL/min) and a solution of TfOH (0.050 M in CH_2Cl_2, flow rate: 12.0 mL/min) were introduced into **M1** using syringe pumps. The mixed solution was passed through **R1** ($ϕ^{R1}$ = 500 μm, L^{R1} = 3.5 cm, t^{R1} = 0.021 s), and was mixed with a solution of neutral nucleophile (0.20 M in CH_2Cl_2, flow rate: 4.0 mL/min) in **M2.** The resulting solution was passed through **R2** (L^{R2} = 1000 cm, t^{R2} = 20 s). After a steady state was reached, an aliquot of the product solution was collected, and was treated with TBAF, Et_3N and brine.

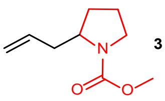
3

Methyl 2-allylpyrrolidine-1-carboxylate (3).

Obtained from **1a** with allyltrimethylsilane in 78% yield determined by GC yield (retention time 15.7 min). After extraction, the crude product was purified by flash chromatography (hexane/EtOAc = 5/2) to afford **3**. The spectral data were identical to those of reported in the literature.[1]

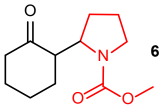

6

Methyl 2-(2-oxocyclohexyl)pyrrolidine-1-carboxylate (6).

Obtained from **1a** with 1-trimethylsiloxy-1-cyclohexene in 60% yield determined by GC (retention time 22.7 min). After extraction, the crude mixture was purified by flash chromatography (hexane/EtOAc = 5/2) to afford **6**. The spectral data were identical to those of reported in the literature.[13]

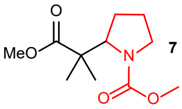

7

Methyl 2-(1-methoxy-2-methyl-1-oxopropan-2-yl)pyrrolidine-1-carboxylate (7)

Obtained from **1a** with 1-methoxy-1-trimethylsilyloxypropene in 70% yield determined by GC (retention time 22.7 min). After extraction, the crude mixture was purified by flash chromatography (hexane/EtOAc = 5/2) to afford **7**. ^{1}H NMR (400 MHz, $CDCl_3$, rotamer) δ 1.13 (d, J = 10.8 Hz, 6 H) , 1.69–1.83 (m, 4 H) , 1.93–1.98 (m, 1 H) , 3.16–3.22 (m, 1 H), 3.63 (s, 3 H) , 3.66 (s, 3 H), 4.26–4.28 (m, 1 H); ^{13}C NMR (100MHz, $CDCl_3$, rotamers) δ 21.8, 22.3, 24.2, 27.3, 47.2, 47.9, 51.8, 52.4, 63.5, 157.6, 177.2; HRMS (EI) calcd for $C_{12}H_{19}NO_3$ $[M]^+$: 225.1365, found 225.1364.

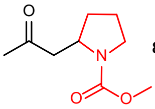

8

Methyl 2-(2-oxopropyl)pyrrolidine-1-carboxylate (8)

Obtained from **1a** with 2-trimethylsiloxypropene in 83% yield determined by GC (retention time: 18.9 min). After extraction, the crude mixture was purified by flash chromatography (hexane/EtOAc = 5/2) to afford **8**. The spectral data were identical to those of reported in the literature.[13]

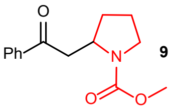

9

Methyl 2-(2-oxo-2-phenylethyl)pyrrolidine-1-carboxylate (9)

Obtained from **1a** with 1-phenyl-1-trimethylsiloxyethylene (T = 0 °C) in 82% yield determined by GC (retention time 26.7 min). After extraction, the crude mixture was purified by flash chromatography (hexane/EtOAc = 5/2) to afford **8**. The spectral data were identical to those of reported in the literature.[13]

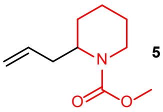

5

Methyl 2-allylpiperidine-1-carboxylate (5)

Obtained from **1b** with allyltrimethylsilane (T = −15 °C, ϕ^{R1} = 1000 μm, L^{R1} = 3.5 cm, t^{R1} = 0.082 s) in 72% yield determined by GC (retention time 16.1 min). After extraction, the crude mixture was purified by flash chromatography (hexane/EtOAc = 5/1) to afford **5**. The spectral data were identical to those of reported in the literature.[13]

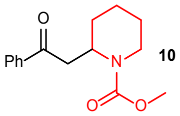

Methyl 2-(2-oxo-2-phenylethyl)piperidine-1-carboxylate (10)

Obtained from **1b** with 1-phenyl-1-trimethylsiloxyethylene ($T = -15$ °C, ϕ^{R1} = 1000 μm, L^{R1} = 3.5 cm, t^{R1} = 0.082 s, quenched by Et_3N) in 81% yield determined by GC. After extraction, the crude mixture was purified by flash chromatography (hexane/EtOAc = 3/1) to afford **10**. The spectral data were identical to those of reported in the literature.[14]

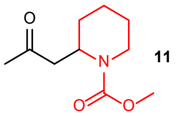

Methyl 2-(2-oxopropyl)piperidine-1-carboxylate (11)

The reaction solution derived from **1b** with 2-trimethylsiloxypropene (T = −15 °C, ϕ^{R1} = 1000 μm, L^{R1} = 3.5 cm, t^{R1} = 0.082 s) was collected for 3.0 min. After working-up, the crude mixture was purified by flash chromatography (hexane/EtOAc = 2/1) to afford **11** in 82% yield (196.8 mg). The spectral data were identical to those of reported in the literature.[14]

Reactions of oxocarbenium ion 2c

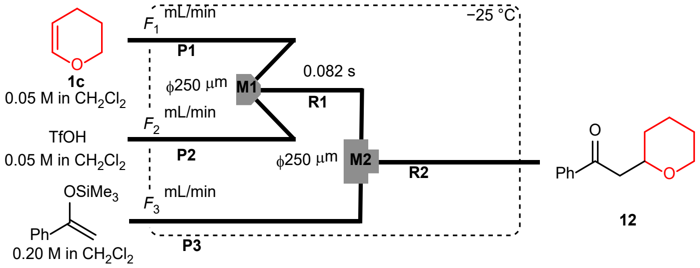

A flow microreactor system consisting of a V-shaped micromixer (**M1**, ϕ = 250 μm) and a T-shaped micromixer (**M2**, ϕ = 250 μm), two microtube reactors (**R1** and **R2**), and three pre-cooling units (**P1**–**P3**) was used. The flow microreactor system was dipped in a cooling bath ($T = -25$ °C). A solution of **1c** (0.0500 M in CH_2Cl_2, flow rate: F_1 mL/min) and a solution of TfOH (0.050 M in CH_2Cl_2, flow rate: F_2 mL/min) were introduced into **M1** using syringe pumps (the sum of F_1 with F_2 is approx. 20 mL/min). The mixed solution was passed through **R1** (L^{R1} = 3.5 cm, t^{R1} = 0.082 s), and was mixed with a solution of 1-phenyl-1-trimethylsiloxyethylene (0.20 M in CH_2Cl_2, flow rate: F_3 mL/min) in **M2.** The resulting solution was passed through **R2** (L^{R2} = 1000 cm, t^{R2} ~ 20 s). After a steady state was reached, an aliquot of the product solution was collected, and was treated with TBAF, Et_3N and brine. The reaction mixture was analyzed by GC with an internal standard (retention time 20.4 min). The results are summarized in Supplementary Table 8. After extraction, the crude mixture was purified by flash chromatography (hexane/EtOAc = 20/1) to afford **2-(2-oxo-2-phenylethyl)tetrahydro-2*H*-pyran** (**12**). The spectral data were identical to those of reported in the literature.[15]

Supplementary Table 8. Yields of **12** with varying equivalent amount of TfOH[a]

F_1 (mL/min)	F_2 (mL/min)	F_3 (mL/min)	equiv. of TfOH	yield of **12** (%)
4	16	4	4.0	54
5	15	3	3.0	48
7	14	2	2.0	34
8	12	1	1.5	10

[a]Yields were determined by GC using an internal standard

Reactions of *N*-acyliminium ion 1a with alkylmetal reagents

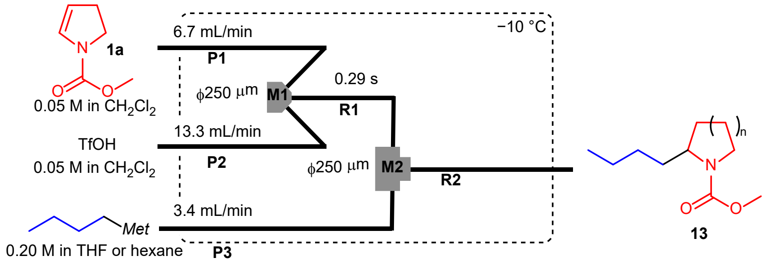

A flow microreactor system consisting of a V-shaped micromixer (**M1**, ϕ = 250 μm) and a T-shaped micromixer (**M2**, ϕ = 250 μm), two microtube reactors (**R1** and **R2**), and three pre-cooling units (**P1**–**P3**) was used. The flow microreactor system was dipped in a cooling bath ($T = -10$ °C). A solution of **1a** (0.0500 M in CH_2Cl_2, flow rate: 6.7 mL/min) and a solution of TfOH (0.050 M in CH_2Cl_2, flow rate: 13.3 mL/min) were introduced into **M1** using syringe pump. The mixed solution was passed through **R1** (L^{R1} = 12.5 cm, t^{R1} = 0.29 s), and was mixed with a solution of alkylmetal reagent (*n*-butyllithium in *n*-hexane, *n*-butylmagnesium chloride in THF, or *n*-butylzinc chloride in THF, flow rate: 3.4 mL/min, concentration: 0.20 M) in **M2.** The resulting solution was passed through **R2** (ϕ^{R2} μm, L^{R2} cm, t^{R2} s). After a steady state was reached, an aliquot of the reacting solution was collected, and was treated with brine. The reaction mixture was analyzed by GC with an internal standard (retention time 15.6 min). The results are summarized in Supplementary Table 9. After extraction, the crude mixture was purified by flash chromatography (hexane/EtOAc = 20/1) to afford **methyl 2-butylpyrrolidine-1-carboxylate** (**13**). The spectral data were identical to those of reported in the literature.[16]

Supplementary Table 9. Yields of **13** from varying counter ion and reaction time[a]

Met	φ^{R2} (μm)	L^{R2} (cm)	t^{R2} (s)	Yield of **13** (%)
Li	500	3.5	0.017	64
	1000	3.5	0.069	72
	1000	12.5	0.25	73
	1000	50	0.98	72
	1000	200	3.9	69
MgCl	500	3.5	0.017	35
	1000	3.5	0.069	40
	1000	12.5	0.25	60
	1000	50	0.98	65
	1000	200	3.9	62
ZnCl	500	3.5	0.017	0
	1000	3.5	0.069	24
	1000	12.5	0.25	19
	1000	50	0.98	33
	1000	200	3.9	32

[a]Yields were determined by GC using an internal standard

2.7 Low-temperature NMR analysis of *N*-acyliminium ion 2a

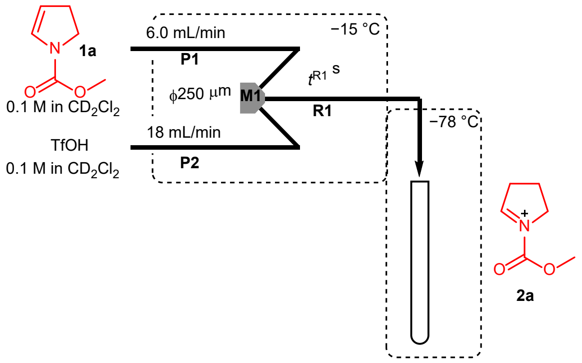

A flow microreactor system consisting of a V-shaped micromixer (**M1**, ϕ = 250 μm), one microtube reactor (**R1**), and two pre-cooling units (**P1**–**P3**) was used. The flow microreactor system was dipped in two cooling baths (−15 and −78 °C). A solution of **1a** (0.10 M in CD_2Cl_2, flow rate: 6.0 mL/min) and a solution of TfOH (0.10 M in CD_2Cl_2, flow rate: 18 mL/min) were introduced into **M1** using syringe pumps. The mixed solution was passed through **R1** (L^{R1} = 10 cm, t^{R1} = 0.20 s), and was introduced into an NMR test tube cooled at −78 °C. Immediately the tube was moved to NMR probe

cooled at −78 °C, and its ^{1}H NMR was measured at −78 °C. ^{1}H NMR (500 MHz, CD_2Cl_2, −78 °C, rotamers) δ 2.48 (t, *J* = 7.0 Hz, 2 H) , 3.49–3.61 (br, 2 H) , 4.10 (s, 3 H), 4.38–4.56 (br, 2 H), 9.49 (s, 1 H).

Oxonium ion 2c

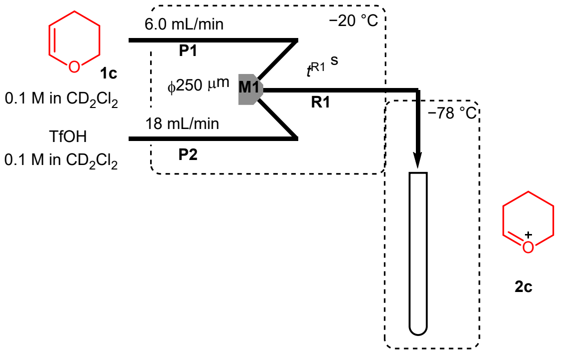

A flow microreactor system consisting of a V-shaped micromixer (**M1**, ϕ = 250 μm), one microtube reactor (**R1**), and two pre-cooling units (**P1**–**P3**) was used. The flow microreactor system was dipped in two cooling baths (−15 and −78 °C). A solution of **1c** (0.10 M in CD_2Cl_2, flow rate: 6.0 mL/min) and a solution of TfOH (0.10 M in CD_2Cl_2, flow rate: 18 mL/min) were introduced into **M1** using syringe pumps. The mixed solution was passed through **R1** (L^{R1} = 10 cm, t^{R1} = 0.20 s), and was introduced into an NMR test tube cooled at −78 °C. Immediately the tube was moved to NMR probe cooled at −78 °C, and its ^{1}H NMR was measured at −78 °C. ^{1}H NMR (500 MHz, CD_2Cl_2) δ 1.93–1.99 (m, 2 H), 2.20–2.26 (m, 2 H), 3.62 (t, *J* = 5.0 Hz, 2 H), 5.39 (t, *J* = 5.0 Hz, 2 H), 10.07 (s, 1 H).

2.8 Reaction of carbocationic species with sp2-carbanions

General procedure

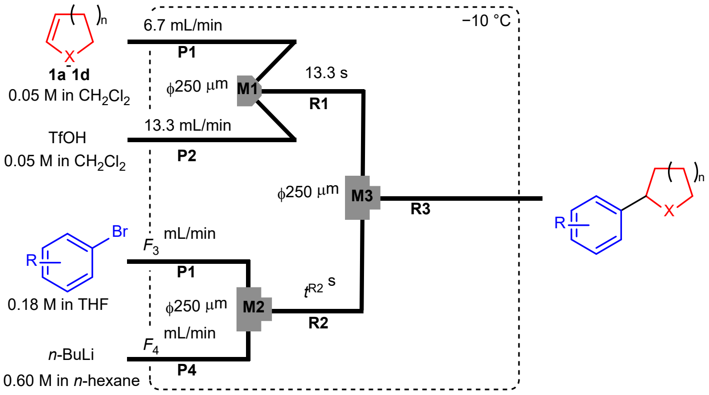

A flow microreactor system consisting of a V-shaped micromixer (**M1**, ϕ = 250 μm), two T-shaped micromixers (**M2** and **M3**, ϕ = 250 μm), three microtube reactors (**R1**–**R3**), and four pre-cooling units

(**P1–P4**) was used. The flow microreactor system was dipped in a cooling bath (T = −10 °C). A solution of the cation precursor (**1a–1d**, 0.0500 M in CH_2Cl_2, flow rate: 6.7 mL/min) and a solution of TfOH (0.050 M in CH_2Cl_2, flow rate: 13.3 mL/min) were introduced into **M1** using syringe pumps. The mixed solution was passed through **R1** (L^{R1} = 12.5 cm, t^{R1} = 0.29 s) to **M3**. Whereas, a solution of aryl halides (0.18 M in THF, flow rate: F_3 = 10 mL/min) and a solution of n-BuLi (0.60 M in n-hexane, flow rate: F_4 = 2.5 mL/min) were introduced into **M2** using syringe pumps, and the mixed solution was passed through **R2** (L^{R2} = 100 cm, t^{R2} = 3.8 s) to **M3**. Those solutions are mixed in **M3**, and the resulting solution was passed through **R3** (L^{R3} = 100 cm, t^{R3} = 1.4 s). After a steady state was reached, an aliquot of the product solution was collected, and was treated with brine. The reaction mixture was analyzed by GC or ^{1}H NMR with an internal standard.

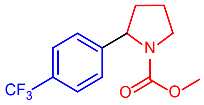

14

Methyl 2-(4-trifluoromethylphenyl)pyrrolidine-1-carboxylate (14)

Obtained from **1a** with 4-bromobenzotrifluoride in 89% yield determined by GC (retention time 20.0 min). After extraction, the crude mixture was purified by flash chromatography (hexane/EtOAc = 4/1) to afford **14**. ^{1}H NMR (400 MHz, $CDCl_3$, rotamers) δ 1.79–1.96 (m, 3 H), 2.28–2.41 (m, 1 H), 3.52–3.73 (m, 5 H), 4.92–5.04 (m, 1 H), 7.24–7.33 (m, 2 H), 7.56 (d, J = 8.0 Hz, 2 H); ^{13}C NMR (100 MHz, $CDCl_3$, rotamers) δ 22.7, 23.7, 34.8, 36.7, 42.3, 47.7, 52.4, 60.6, 61.2, 124.4 (q, J = 271.2 Hz), 125.4, 125.9, 129.0 (q, J = 31.6 Hz), 148.0, 148.4, 155.7; ^{19}F NMR (376 MHz, $CDCl_3$, rotamers) δ −62.3, −62.2; HRMS (ESI) calcd for $C_{13}H_{14}F_3NO_2Na$ $[M+Na]^+$: 296.0857, found: 296.0863.

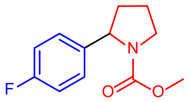

15

Methyl 2-(4-fluorophenyl)pyrrolidine-1-carboxylate (15)

Obtained from **1a** with 1-bromo-4-fluorobenzene (F_3 = 8.0 mL/min, F_4 = 2.0 mL/min, t^{R2} = 4.7 s, t^{R3} = 1.6 s) in 92% yield determined by GC (retention time 19.9 min). After extraction, the crude mixture was purified by flash chromatography (hexane/EtOAc =4/1) to afford **15**. The spectral data were identical to those of reported in the literature.[17]

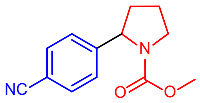

16

Methyl 2-(4-cyanophenyl)pyrrolidine-1-carboxylate (16)

Obtained from **1a** with 4-bromobenzonitrile (F_3 = 8.0 mL/min, F_4 = 2.0 mL/min, L^{R2} = 3.5 cm, t^{R2} = 0.16 s, t^{R3} = 1.6 s) in 66% yield determined by ^{1}H NMR using 1,1,2,2-tetrachloroethane as an internal standard. After extraction, the crude product was purified by flash chromatography (hexane/EtOAc = 3/2) to afford **16**. ^{1}H NMR (400 MHz, $CDCl_3$, rotamers)

δ 1.72–1.92 (m, 1 H), 2.26–2.40 (m, 3 H), 3.47–3.69 (m, 5 H), 4.86–4.97 (m, 1 H), 7.20–7.30 (m, 2 H), 7.56 (d, J = 8.0 Hz, 2 H); ^{13}C NMR (100 MHz, $CDCl_3$, rotamers) δ 22.9, 23.9, 34.8, 35.8, 47.4, 47.8, 52.7, 60.8, 61.3, 77.6, 110.7, 119.1, 126.3, 126.5, 132.5, 149.4, 149.9, 155.7; HRMS (ESI) calcd for $C_{13}H_{14}N_2O_2$ Na [M+Na]$^+$: 253.0942, found: 235.0942.

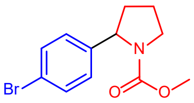

17

Methyl 2-(4-bromophenyl)pyrrolidine-1-carboxylate (17)

Obtained from **1a** with 1,4-dibromobenzene (F_3 = 8.0 mL/min, F_4 = 2.0 mL/min, t^{R2} = 4.7 s, t^{R3} = 1.6 s) in 76% yield determined by GC (retention taime 22.7 min). After extraction, the crude mixture was purified by flash chromatography (hexane/EtOAc = 4/1) to afford **17**. ^{1}H NMR (400 MHz, $CDCl_3$, rotamers) δ 1.70–1.94 (m, 3 H), 2.23–2.37 (m, 1 H), 3.50–3.74 (m, 5 H), 4.83–4.95 (m, 1 H), 6.99–7.11 (m, 2 H), 7.42 (d, J = 8.0 Hz, 2 H); ^{13}C NMR (100 MHz, $CDCl_3$, rotamers) δ 22.8, 23.9, 34.9, 35.8, 47.2, 47.7, 52.6, 60.5, 61.0, 120.5, 120.6, 127.3, 127.5, 131.6, 142.9, 143.4, 155.7, 155.9; HRMS (ESI) calcd for $C_{12}H_{14}BrNO_2Na$ [M+Na]$^+$: 306.0093, found: 306.0093.

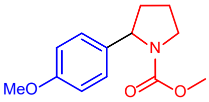

18

Methyl 2-(4-methoxyphenyl)pyrrolidine-1-carboxylate (18)

Obtained from **1a** with 4-bromoanisole (F_3 = 8.0 mL/min, F_4 = 2.0 mL/min, t^{R2} = 4.7 s, t^{R3} = 1.6 s) in 77% yield determined by GC (retention time 22.3 min). After extraction, the crude mixture was purified by flash chromatography (hexane/EtOAc = 3/2) to afford **18**. ^{1}H NMR (400 MHz, $CDCl_3$, rotamers) δ 1.78–2.02 (m, 3 H), 2.18–2.36 (m, 1 H), 3.49–3.73 (m, 5 H), 3.79 (s, 3 H), 4.83–4.96 (m, 1 H), 6.82–6.89 (m, 2 H), 7.03–7.18 (m, 2 H); ^{13}C NMR (100 MHz, $CDCl_3$, rotamers) δ 22.4, 23.6, 34.6, 35.6, 46.9, 47.3, 52.2, 55.1, 60.1, 60.6, 113.6, 126.3, 126.5, 135.6, 136.0, 155.4, 155.8, 158.3; HRMS (EI) calcd for $C_{13}H_{17}NO_3$ [M]$^+$: 235.1208, found 235.1208.

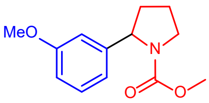

19

Methyl 2-(3-methoxyphenyl)pyrrolidine-1-carboxylate (19)

Obtained from **1a** with 3-bromoanisole in 83% yield determined by GC (retention time 21.9 min). After extraction, the crude mixture was purified by flash chromatography (hexane/EtOAc = 3/2) to afford **19**. ^{1}H NMR (400 MHz, $CDCl_3$, rotamers) δ 1.80–2.00 (m, 3 H), 2.20–2.37 (m, 1 H), 3.49–3.73 (m, 5 H), 3.79 (s, 3 H), 4.85–4.99 (m, 1 H), 6.66–6.81 (m, 3 H),

7.18–7.25 (m 1 H); ^{13}C NMR (100 MHz, $CDCl_3$, rotamers) δ 22.8, 23.8, 34.9, 35.8, 47.3, 47.7, 52.6, 55.4, 60.9, 61.4, 111.5, 111.8, 117.9, 118.1, 129.6, 145.6, 146.1, 155.8, 156.1, 159.8; HRMS (ESI) calcd for $C_{13}H_{17}NO_3Na$ $[M+Na]^+$: 258.1101, found: 258.1093.

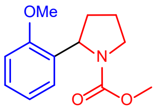

20

Methyl 2-(2-methoxyphenyl)pyrrolidine-1-carboxylate (20)

Obtained from **1a** with 2-bromoanisole (F_3 = 8.0 mL/min, F_4 = 2.0 mL/min, t^{R2} = 4.7 s, t^{R3} = 1.6 s) in 72% yield determined by GC (retention time 21.3 min). After extraction, the crude mixture was purified by flash chromatography (hexane/EtOAc = 3/2) to afford **20**. ^{1}H NMR (400 MHz, $CDCl_3$, rotamers) δ 1.72–1.95 (m, 3 H), 2.16–2.33 (m, 1 H), 3.46–3.78 (m, 5 H), 3.82–3.98 (m, 3H), 5.14–5.34 (m, 1 H), 6.82–7.13 (m, 3 H), 7.18–7.28 (m, 1 H); ^{13}C NMR (100 MHz, $CDCl_3$, rotamers) δ 22.5, 23.5, 32.9, 33.7, 47.1, 47.6, 52.5, 55.3, 56.4, 56.8, 110.3, 110.5, 120.3, 125.5, 125.6, 127.8, 131.4, 131.9, 155.4, 156.0, 156.2; HRMS (ESI) calcd for $C_{13}H_{17}NO_3Na$ $[M+Na]^+$: 258.1101, found: 258.1093.

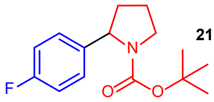

21

***tert*-Butyl 2-(4-fluorophenyl)pyrrolidine-1-carboxylate (21)**

Obtained from **1d** with 1-bromo-4-fluorobenzene (F_3 = 20 mL/min, F_4 = 5.0 mL/min, t^{R2} = 1.9 s, t^{R3} = 1.0 s) in 80% yield determined by GC (retention time 20.1 min). After extraction, the crude mixture was purified by flash chromatography (hexane/EtOAc = 4/1) to afford **21**. The spectral data were identical to those of reported in the literature.[18]

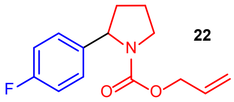

22

Allyl 2-(4-fluorophenyl)pyrrolidine-1-carboxylate (22)

Obtained from **1e** with 1-bromo-4-fluorobenzene (F_3 = 8.0 mL/min, F_4 = 2.0 mL/min, t^{R2} = 4.7 s, t^{R3} = 1.6 s) in 85% yield determined by GC (retention time 21.0 min). After extraction, the crude mixture was purified by flash chromatography (hexane/EtOAc = 4/1) to afford **22**. ^{1}H NMR (400 MHz, $CDCl_3$) (mixture of rotamers) δ 1.78–1.98 (m, 3 H), 2.23–2.38 (m, 1 H), 3.56–3.71 (m, 2 H), 4.39–4.64 (m, 2 H), 4.88–5.03 (m, 2 H), 5.18–5.36 (m, 1 H), 5.61–6.01 (m, 1 H), 7.00 (t, J = 8.4 Hz, 2 H), 7.10–7.20 (m, 2 H); ^{13}C NMR (100 MHz, $CDCl_3$, rotamers) δ 23.0, 23.9, 35.1, 36.1, 47.3, 47.8, 60.6, 60.9, 65.7, 66.0, 115.2, 115.3, 115.5, 115.5, 116.7, 117.5, 127.2, 133.2 (d, J = 37.4 Hz), 139.8 (d, J = 60.3 Hz), 155.0, 161.9 (d, J = 243.4 Hz); ^{19}F NMR (376 MHz, $CDCl_3$) δ −116.4; HRMS (ESI)

calcd for $C_{14}H_{16}FNO_2Na$ $[M+Na]^+$: 272.1067, found: 272.1053.

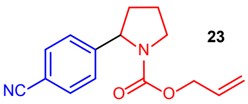

23

Allyl 2-(4-cyanophenyl)pyrrolidine-1-carboxylate (23)

Obtained from **1e** with 4-bromobenzonitrile (F_3 = 16 mL/min, F_4 = 4.0 mL/min, L^{R2} = 3.5 cm, t^{R2} = 0.082 s, L^{R3} = 30 cm, t^{R3} = 0.35 s) in 70% yield determined by GC (retention time 25.7 min). After extraction, the crude mixture was purified by flash chromatography (hexane/EtOAc = 4/1) to afford **23**. ^{1}H NMR (400 MHz, $CDCl_3$, rotamers) δ 1.78–1.99 (m, 3 H), 2.30–2.45 (m, 1 H), 3.60–3.74 (m, 2 H), 4.38–4.64 (m, 2 H), 4.87–5.06 (m, 2 H), 5.17–5.37 (m, 1 H), 5.58–6.00 (m, 1 H), 7.23–7.34 (m, 2 H), 7.61 (d, J = 8.0 Hz, 2 H); ^{13}C NMR (100 MHz, $CDCl_3$, rotamers) δ 23.1, 24.0, 34.9, 35.9, 47.4, 47.9, 61.0, 61.4, 65.8, 66.1, 110.8, 117.0, 117.7, 119.1, 126.4, 126.5, 132.5, 132.7, 133.1, 149.4, 150.0, 154.9; HRMS (ESI) calcd for $C_{15}H_{16}N_2O_2Na$ $[M+Na]^+$: 279.1104, found: 279.1102.

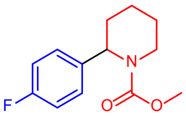

24

Methyl 2-(4-fluorophenyl)piperidine-1-carboxylate (24)

Obtained from **1b** with 1-bromo-4-fluorobenzene (F_3 = 8.0 mL/min, F_4 = 2.0 mL/min, ϕ^{R1} = 500 μm, L^{R1} = 3.5 cm, t^{R1} = 0.021 s, t^{R2} = 4.7 s, t^{R3} = 1.6 s) in 73% yield determined by GC (retention time 21.0 min). After extraction, the crude mixture was purified by flash chromatography (hexane/EtOAc = 4/1) to afford **24**. ^{1}H NMR (400 MHz, $CDCl_3$, rotamers) δ 1.35–1.67 (m, 4 H), 1.84–1.95 (m, 1 H), 2.25–2.32 (m, 1 H), 2.73–2.82 (m, 1 H), 3.74 (s, 3 H), 4.03–4.12 (m, 1 H), 5.40–5.48 (m, 1 H), 7.03 (t, J = 8.2 Hz, 2 H), 7.16–7.21 (m, 2 H); ^{13}C NMR (100 MHz, $CDCl_3$, rotamers) δ 19.4, 25.6, 28.3, 40.5, 53.0, 53.1, 115.5, 115.7, 128.3, 128.4, 135.6, 156.9, 161.8 (d, J = 243.4 Hz); ^{19}F NMR (376 MHz, $CDCl_3$, rotamers) δ −116.7; HRMS (ESI) calcd for $C_{13}H_{16}FNO_2Na$ $[M+Na]^+$: 260.1057, found: 260.1054.

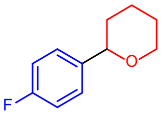

25

2-(4-Fluorophenyl)tetrahydro-2*H*-pyran (25)

Obtained from **1c** and 1-bromo-4-fluorobenzene (F_3 = 16 mL/min, F_4 = 4.0 mL/min, L^{R1} = 25 cm, t^{R1} = 0.59 s, t^{R2} = 2.4 s, t^{R3} = 1.2 s) in 48% yield determined by GC (retention time 14.8 min). After extraction, the crude mixture was purified by flash chromatography (hexane/EtOAc = 4/1) to afford **23**. The spectral data were identical to those of reported in the

literature.[19]

2.9 Reaction of carbocationic species with sp-carbanions

General procedure

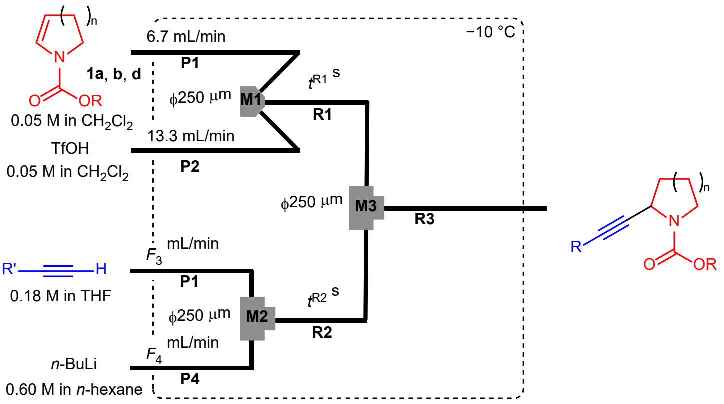

A flow microreactor system consisting of a V-shaped micromixer (**M1**, ϕ = 250 μm), two T-shaped micromixers (**M2** and **M3**, ϕ = 250 μm), three microtube reactors (**R1**–**R3**), and four pre-cooling units (**P1**–**P4**) was used. The flow microreactor system was dipped in a cooling bath (T = −10 °C). A solution of the cation precursor (**1a**, **b**, and **d**, 0.0500 M in CH_2Cl_2, flow rate: 6.7 mL/min) and a solution of TfOH (0.050 M in CH_2Cl_2, flow rate: 13.3 mL/min) were introduced into **M1** using syringe pumps. The mixed solution was passed through **R1** (ϕ^{R1} = 1000 μm, L^{R1} = 12.5 cm, t^{R1} = 0.29) to **M3**. Whereas, a solution of aryl halides (0.18 M in THF, flow rate: 8.0 mL/min) and a solution of *n*-BuLi (0.60 M in *n*-hexane, flow rate: 2.0 mL/min) were introduced into **M2** using syringe pumps, and the mixed solution was passed through **R2** (L^{R2} = 12.5 cm, t^{R2} = 0.59 s) to **M3**. Those solutions are mixed in **M3**, and the resulting solution was passed through **R3** (L^{R3} = 300 cm, t^{R3} = 4.7 s). After a steady state was reached, an aliquot of the product solution was collected, and was treated with brine. The reaction mixture was analyzed by GC using an internal standard.

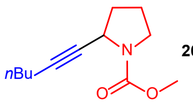

26

Methyl 2-(1-hexyn-1-yl)pyrrolidine-1-carboxylate (26)

Obtained from **1a** with 1-hexyne in 96% yield determined by GC (retention time 18.5 min). After extraction, the crude mixture was purified by flash chromatography (hexane/EtOAc = 4/1) to afford **26**. ^{1}H NMR (400 MHz, $CDCl_3$, rotamers) δ 0.87 (t, J = 6.8 Hz, 3 H), 1.29–1.48 (m, 4 H), 1.83–2.18 (m, 6 H), 3.21–3.39 (m, 1 H), 3.40–3.54 (m, 1 H), 3.70 (s, 3 H), 4.39–4.58 (m, 1 H); ^{13}C NMR (100 MHz, $CDCl_3$, rotamers) δ 13.9, 18.6, 22.1, 23.8, 24.7, 31.1, 33.7, 34.5, 45.9, 46.3, 48.6, 49.0, 52.6, 80.1, 80.4, 82.7, 155.6; HRMS (ESI) calcd for $C_{12}H_{19}NO_2Na$ [M+Na]$^+$: 232.1305, found:

232.1303.

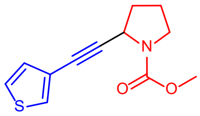

30

Methyl 2-(thiophen-3-ylethynyl)pyrrolidine-1-carboxylate (30)

Obtained from **1a** with 3-ethynylthiophene (F_3 = 16 mL/min, F_4 = 4.0 mL/min, L^{R2} = 100 cm, t^{R2} = 2.4 s, L^{R3} = 200 cm, t^{R3} = 2.4 s) in 84% yield determined by GC (retention time 22.3 min). After extraction, the crude mixture was purified by flash chromatography (hexane/EtOAc = 3/1) to afford **30**. ^{1}H NMR (400 MHz, $CDCl_3$, rotamers) δ 1.90–2.02 (m, 1 H), 2.06–2.24 (m, 3 H), 3.30–3.62 (m, 2 H), 3.70–3.80 (m, 3 H), 4.64–4.83 (m, 1 H), 7.08 (br, 1 H), 7.23 (br, 1 H), 7.40 (br, 1 H); ^{13}C NMR (100 MHz, $CDCl_3$, rotamers) δ 23.8, 24.7, 33.3, 34.1, 45.9, 46.2, 48.8, 49.2, 52.6, 52.7, 77.3, 77.6, 88.9, 89.0, 122.0, 122.1, 125.2, 125.4, 128.7, 128.8, 130.0, 130.2, 155.2, 155.3; HRMS (ESI) calcd for $C_{12}H_{13}NO_2SNa$ $[M+Na]^+$: 258.0559, found: 258.0553.

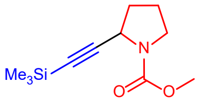

31

Methyl 2-(trimethylsilylethynyl)pyrrolidine-1-carboxylate (31)

Obtained from **1a** with trimethylsilylacetylene (F_3 = 12 mL/min, F_4 = 3.0 mL/min, L^{R2} = 100 cm, t^{R2} = 3.1 s, L^{R3} = 100 cm, t^{R3} = 1.3 s) in 89% yield determined by GC (retention time 16.4 min). After extraction, the crude mixture was purified by flash chromatography (hexane/EtOAc = 9/1) to afford **31**. ^{1}H NMR (400 MHz, $CDCl_3$, rotamers) δ 0.09 (s, 9 H), 1.77–1.92 (m, 1 H) 1.93–2.14 (m, 3 H), 3.20–3.53 (m, 2 H), 3.68 (s, 3 H), 4.37–4.60 (m, 1 H); ^{13}C NMR (100 MHz, $CDCl_3$, rotamers) δ 0.20, 23.8, 24.7, 33.6, 34.2, 45.9, 46.3, 48.9, 49.3, 52.6, 86.4, 86.5, 105.9, 106.0, 155.5; HRMS (ESI) calcd for $C_{11}H_{19}NO_2SiNa$ $[M+Na]^+$: 248.1077, found: 248.1072.

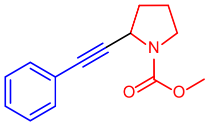

32

Methyl 2-(phenylethynyl)pyrrolidine-1-carboxylate (32)

Obtained from **1a** with phenylacetylene (F_3 = 16 mL/min, F_4 = 4.0 mL/min, L^{R2} = 100 cm, t^{R2} = 2.4 s, L^{R3} = 100 cm, t^{R3} = 1.2 s) in 92% yield determined by GC (retention time 22.2 min). After extraction, the crude mixture was purified by flash chromatography (hexane/EtOAc = 5/1) to afford **32**. The spectral data were identical to those of reported in the literature.[20]

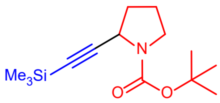

33

***tert*-Butyl 2-(trimethylsilylethynyl)pyrrolidine-1-carboxylate (33)**

Obtained from **1d** with trimethylsilylacetylene (F_3 = 16 mL/min, F_4 = 4.0 mL/min, L^{R2} = 100 cm, t^{R2} = 2.4 s, L^{R3} = 100 cm, t^{R3} = 1.2 s) in 92% yield determined by GC (retention time 17.1 min). After extraction, the crude mixture was purified by flash chromatography (hexane/EtOAc = 9/1) to afford **33**. The spectral data were identical to those of reported in the literature.[21]

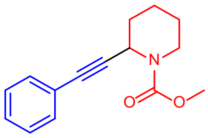

34

Methyl 2-(phenylethynyl)piperidine-1-carboxylate (34)

Obtained from **1b** and phenylacetylene (F_3 = 12 mL/min, F_4 = 3.0 mL/min, ϕ^{R1} = 500 μm, L^{R1} = 3.5 cm, t^{R1} = 0.021 s, L^{R2} = 100 cm, t^{R2} = 3.1 s, L^{R3} = 100 cm, t^{R3} = 1.3 s) in 75% yield determined by GC (retention time 22.9 min). After extraction, the crude mixture was purified by flash chromatography (hexane/EtOAc = 9/1) to afford **34**. The spectral data were identical to those of reported in the literature.[20]

Procedure for different reaction temperatures

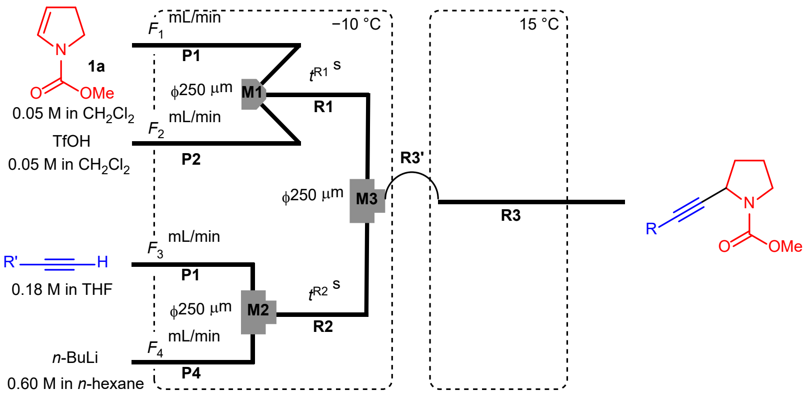

A flow microreactor system consisting of a V-shaped micromixer (**M1**, ϕ = 250 μm) and two T-shaped micromixers (**M2** and **M3**, ϕ = 250 μm), three microtube reactors (**R1**–**R3**), a PTFE tube (**R3'**), and four pre-cooling units (**P1**–**P4**) was used. The former part of the flow microreactor system (from the pre-cooling units to **M3**) was dipped in a cooling bath at −10 °C, whereas **R3** is dipped in a water bath at 15 °C. The tubes in different baths were connected by **R3'**. A solution of the cation precursor (**1a**, 0.0500 M in CH_2Cl_2, flow rate: 6.7 mL/min) and a solution of TfOH (0.050 M in CH_2Cl_2, flow rate: 13.3 mL/min) were introduced into **M1** using syringe pumps. The mixed solution was passed through **R1** (L^{R1} = 12.5 cm, t^{R1} = 0.29 s) to **M3**. Whereas, a solution of aryl halides (0.18 M in THF, flow rate: F_3 =8.0 mL/min) and a solution of *n*-BuLi (0.60 M in *n*-hexane, flow rate: F_4 = 2.0 mL/min) were

introduced into **M2** using syringe pumps, and the mixed solution was passed through **R2** (L^{R2} = 12.5 cm, t^{R2} = 0.59 s) to **M3**. Those solutions are mixed in **M2**, and the resulting solution was passed through **R3'** ($L^{R3'}$ = 10 cm, $t^{R3'}$ = 0.16 s) and **R3** (L^{R3} = 200 cm, t^{R3} = 3.1 s). After a steady state was reached, an aliquot of the product solution was collected, and was treated with brine. The reaction mixture was analyzed by GC using an internal standard.

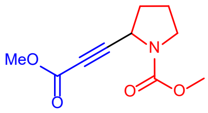

27

Methyl 2-(3-methoxy-3-oxopropynyl)pyrrolidine-1-carboxylate (27)
Obtained from **1a** with methyl propiolate in 80% yield determined by GC (retention time 20.4 min). After extraction, the crude mixture was purified by flash chromatography (hexane/EtOAc = 3/1) to afford **27**. ^{1}H NMR (400 MHz, $CDCl_3$, rotamers) δ 1.91–2.02 (m, 1 H), 2.03–2.21 (m, 3 H), 3.29–3.59 (m, 2 H), 3.70–3.79 (m, 6 H), 4.56–4.71 (m, 1 H); ^{13}C NMR (100 MHz, $CDCl_3$, rotamers) δ 23.8, 24.8, 32.4, 33.3, 45.9, 46.3, 48.0, 52.8, 52.8, 73.8, 87.4, 153.9, 155.0, 155.1; HRMS (ESI) calcd for $C_{10}H_{13}NO_4Na$ $[M+Na]^+$: 234.0737, found: 234.0732.

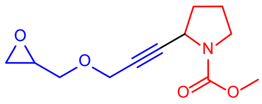

28

Methyl 2-(3-glycidyloxy-1-propynyl)pyrrolidine-1-carboxylate (28)
Obtained in from **1a** with glycidyl propargyl ether (F_3 = 16 mL/min, F_4 = 8.0 mL/min, t^{R2} = 0.29 s, $t^{R3'}$ = 0.12 s t^{R3} = 2.4 s) in 70% yield determined by GC (retention time 22.7 min). After extraction, the crude mixture was purified by flash chromatography (hexane/EtOAc = 3/2) to afford **28**. ^{1}H NMR (400 MHz, $CDCl_3$, rotamers) δ 1.87–1.97 (m, 1 H), 1.98–2.16 (m, 3 H), 2.63 (dd, J = 5.2 Hz, 2.4 Hz, 1 H), 2.81 (t, J = 4.8 Hz, 1 H), 3.14–3.19 (m, 1 H), 3.42–3.56 (m, 3 H), 3.67–3.81 (m, 4 H), 4.15–4.27 (m, 2 H), 4.49–4.65 (m, 1 H); ^{13}C NMR (100 MHz, $CDCl_3$, rotamers) δ 23.7, 24.6, 33.1, 33.9, 44.4, 45.8, 46.1, 50.5, 52.5, 58.9, 70.3, 77.5, 86.7, 86.9, 155.2; HRMS (ESI) calcd for $C_{12}H_{17}NO_4Na$ $[M+Na]^+$: 262.1050, found: 262.1046.

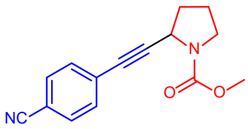

29

Methyl 2-(4-cyanophenylethynyl)pyrrolidine-1-carboxylate (29)
Obtained from **1a** with 4-ethynylbenzonitrile in 90% yield determined by GC (retention time 26.6 min). After extraction, the crude mixture was purified by flash chromatography (hexane/EtOAc = 3/1) to afford **29**. ^{1}H NMR (400 MHz, $CDCl_3$, rotamers) δ 1.94–2.04 (m, 1 H), 2.09–2.23 (m, 3 H), 3.32–3.62 (m, 2 H), 3.72–3.78 (m, 3 H), 4.68–4.85 (m, 1 H), 7.45–7.51 (m, 2 H), 7.55–7.61 (m, 2 H); ^{13}C NMR (100 MHz, $CDCl_3$,

rotamers) δ 23.8, 24.7, 33.1, 33.9, 45.9, 46.2, 48.6, 49.1, 52.6, 52.7, 77.6, 80.6, 94.0, 111.4, 118.5, 128.0, 132.0, 132.4, 155.1; HRMS (ESI) calcd for $C_{15}H_{14}N_2O_2Na$ $[M+Na]^+$: 277.0945, found: 277.0942.

Procedure for thionium ion

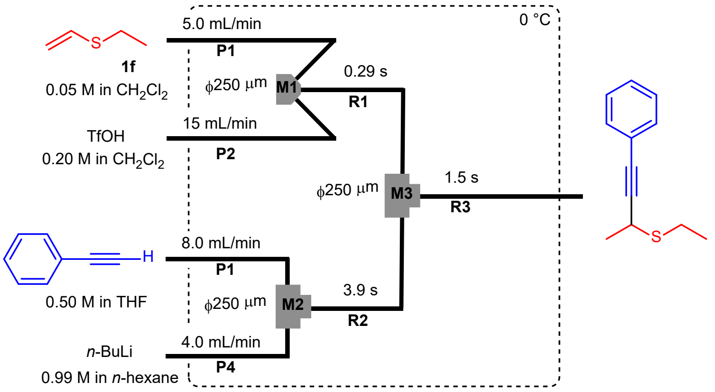

A flow microreactor system consisting of a V-shaped micromixer (**M1**, ϕ = 250 μm) and two T-shaped micromixers (**M2** and **M3**, ϕ = 250 μm), three microtube reactors (**R1**–**R3**), and four pre-cooling units (**P1**–**P4**) was used. The flow microreactor system was dipped in a cooling bath (T = 0 °C). A solution of the cation precursor (**1f**, 0.0500 M in CH_2Cl_2, flow rate: 5.0 mL/min) and a solution of TfOH (0.20 M in CH_2Cl_2, flow rate: 15 mL/min) were introduced into **M1** using syringe pumps. The mixed solution was passed through **R1** (ϕ^{R1} = 1000 μm, L^{R1} = 12.5 cm, t^{R1} = 0.29) to **M3**. Whereas, a solution of phenylacetylene (0.50 M in THF, flow rate: 8.0 mL/min) and a solution of *n*-BuLi (0.99 M in *n*-hexane, flow rate: 4.0 mL/min) were introduced into **M2** using syringe pumps, and the mixed solution was passed through **R2** (L^{R2} = 100 cm, t^{R2} = 3.9 s) to **M3**. Those solutions are mixed in **M3**, and the resulting solution was passed through **R3** (L^{R3} = 100 cm, t^{R3} = 1.5 s). After a steady state was reached, an aliquot of the product solution was collected, and was treated with brine. The reaction mixture was analyzed by GC using an internal standard.

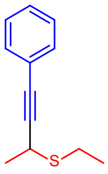

35

Ethyl 4-phenyl-3-butyn-2-yl sulfide (35)

Obtained from ethyl vinyl sulfide (**1f**) with phenylacetylene in 61% yield determined by GC (retention time 16.6 min). After extraction, the crude mixture was purified by flash chromatography and GPC to afford **35**. ^{1}H NMR (400 MHz, $CDCl_3$, rotamers) δ 1.33 (t, J = 7.6 Hz, 3 H), 1.58 (d, J = 7.2 Hz, 3 H), 2.67–2.89 (m, 2 H), 3.88 (q, J = 7.2 Hz, 1 H), 7.28–7.31 (m, 3 H), 7.41–7.43 (m, 2 H); ^{13}C NMR (100 MHz, $CDCl_3$) δ 15.0, 22.1, 25.8, 29.8, 83.2, 90.5, 123.6, 128.4, 128.6, 132.0; HRMS (APCI) calcd for

$C_{12}H_{15}S$ [M]$^+$: 191.0889, found: 191.0888.

2.10 Twice direct cross-coupling reaction

General procedure

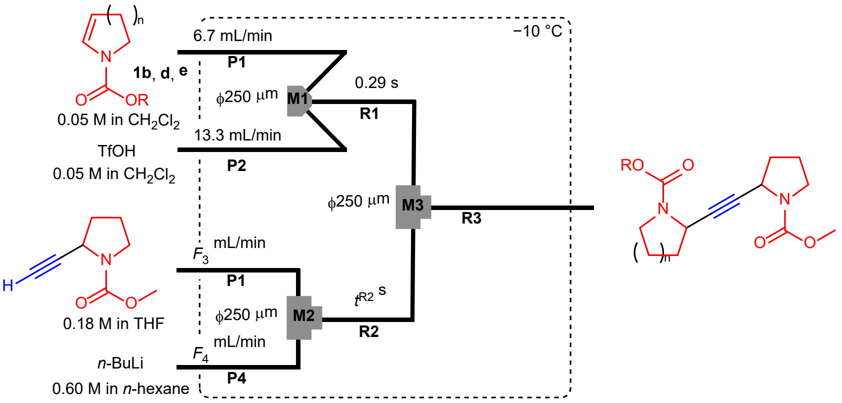

A flow microreactor system consisting of a V-shaped micromixer (**M1**, ϕ = 250 μm) and two T-shaped micromixers (**M2** and **M3**, ϕ = 250 μm), three microtube reactors (**R1**–**R3**), and four pre-cooling units (**P1**–**P4**) was used. The flow microreactor system was dipped in a cooling bath (T = −10 °C). A solution of the cation precursor (**1b**, **d**, and **e**, 0.0500 M in CH_2Cl_2, flow rate: 6.7 mL/min) and a solution of TfOH (0.050 M in CH_2Cl_2, flow rate: 13.3 mL/min) were introduced into **M1** using syringe pumps. The mixed solution was passed through **R1** (L^{R1} = 12.5 cm, t^{R1} = 0.29 s) to **M3**. Whereas, a solution of alkynyl pyrrolidine (0.18 M in THF, flow rate: F_3 = 16 mL/min) and a solution of n-BuLi (0.60 M in n-hexane, flow rate: F_4 = 4.0 mL/min) were introduced into **M2** using syringe pumps, and the mixed solution was passed through **R2** (L^{R2} = 25 cm, t^{R2} = 0.59 s) to **M3**. Those solutions are mixed in **M2**, and the resulting solution was passed through **R3** (L^{R3} = 100 cm, t^{R3} = 1.2 s). After a steady state was reached, an aliquot of the product solution was collected, and was treated with brine. The reaction mixture was analyzed by GC using an internal standard.

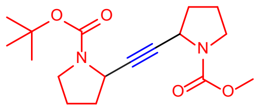

36

***tert*-Butyl 2-((1-(methoxycarbonyl)pyrrolidin-2-yl)ethynyl)pyrrolidine-1-carboxylate (36)**

Obtained from **1d** (F_3 = 8.0 mL/min, F_4 = 2.0 mL/min, t^{R2} 1.2 s, t^{R3} 1.6 s) in 62% yield determined by GC (retention time 25.1 and 25.3 min), indicating diastereomixture (1:1 ratio). After extraction, the crude mixture was purified by flash chromatography (hexane/EtOAc = 1/1) to afford **36**. ^{1}H NMR (400 MHz, $CDCl_3$, rotamer, diasteromixture) δ 1.46 (s, 9 H), 1.79–2.16 (m, 8 H), 3.19–3.54 (m, 4 H), 3.71 (s, 3 H),

4.35–4.61 (m, 2 H); ^{13}C NMR (100 MHz, $CDCl_3$, rotamer, diastereomixture) δ 23.8, 23.9, 24.7, 28.7, 33.5, 34.1, 34.3, 45.8, 46.5, 48.4, 48.8, 52.6, 77.6, 79.7, 81.4, 82.7, 154.3, 155.4; HRMS (ESI) calcd for $C_{16}H_{22}N_2O_4Na$ $[M+Na]^+$: 329.1472, found: 329.1469.

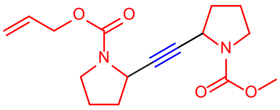

37

Allyl 2-((1-(methoxycarbonyl)pyrrolidin-2-yl)ethynyl)pyrrolidine-1-carboxylate (37)

Obtained from **1e** (t^{R2} 0.59 s, t^{R3} 1.6 s) in 75% yield determined by GC (retention time 25.1 and 25.3 min), indicating diastereomixture (1;1 ratio). After extraction, the crude mixture was purified by flash chromatography (hexane/EtOAc = 1/1) to afford **37**. ^{1}H NMR (400 MHz, $CDCl_3$, rotamer, diasteromixture) δ 1.76–2.15 (m, 8 H), 3.23–3.56 (m, 4 H), 3.71 (s, 3 H), 4.41–4.76 (m, 4 H), 5.14–5.26 (m, 1 H), 5.27–5.35 (m, 1 H), 5.86–6.01 (m, 1 H); ^{13}C NMR (100 MHz, $CDCl_3$, rotamer, diastereomixture) δ 23.8, 24.6, 33.4, 34.2, 45.9, 46.2, 48.3, 48.8, 52.6, 65.8, 65.8, 77.6, 82.0, 116.9, 117.4, 133.3, 154.6, 155.2; HRMS (ESI) calcd for $C_{17}H_{26}N_2O_4Na$ $[M+Na]^+$: 345.1785, found: 345.1781.

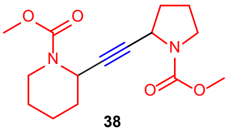

38

Methyl 2-((1-(methoxycarbonyl)pyrrolidin-2-yl)ethynyl)piperidine-1-carboxylate (38)

Obtained from **1b** (t^{R2} 0.59 s, t^{R3} 1.6 s) in 80% yield determined by GC (retention time 25.7 and 25.9 min), indicating diastereomixture (1:1 ratio). After extraction, the crude mixture was purified by flash chromatography (hexane/EtOAc = 1/1) to afford **35**. ^{1}H NMR (400 MHz, $CDCl_3$, rotamers, diastereomixture) δ 1.26–1.46 (m, 1 H), 1.48–1.80 (m, 5 H), 1.83–2.20 (m, 4 H), 2.90–3.13 (m, 1 H), 3.21–3.57 (m, 2 H), 3.65 (s, 3 H), 3.67 (s, 3 H), 3.81–4.08 (m, 1 H), 4.38–4.65 (m, 1 H), 4.88–5.28 (m, 1 H); ^{13}C NMR (100 MHz, $CDCl_3$, rotamer. diastereomixture) δ 20.1, 23.9, 24.8, 25.5, 31.0, 33.5, 34.2, 40.7, 44.6, 45.9, 46.3, 48.4, 48.8, 52.6, 52.9, 80.0, 84.8, 155.7, 155.4; HRMS (ESI) calcd for $C_{15}H_{22}N_2O_4Na$ $[M+Na]^+$: 317.1472, found: 317.1468.

Deprotection of trimethylsilyl group of 31

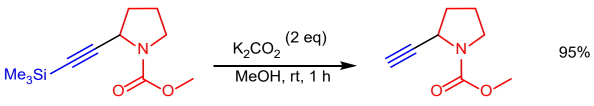

31

To a solution of **31** (1.87 g, 8.30 mmol) in MeOH (40 mL), was added K_2CO_3 (2.30 g, 16.6 mmol). After the mixture was stirred at room temperature for 1 h, it was treated with a saturated aqueous NH_4Cl. The organic layer was separated, and the aqueous layer was extracted with EtOAc three times. After the combined organic extracts were dried over Na_2SO_4, the solid was filtered off and the solvent was evaporated under a reduced pressure. The crude product was purified by flash chromatography using hexane/EtOAc (5/1) as an eluent to afford **methyl 2-ethynylpyrrolidine-1-carboxylate** as a colorless oil (1.21 g, 95%). The spectral data were identical to those of reported in the literature.[22]

3. ^{1}H, ^{13}C, and ^{19}F NMR spectra

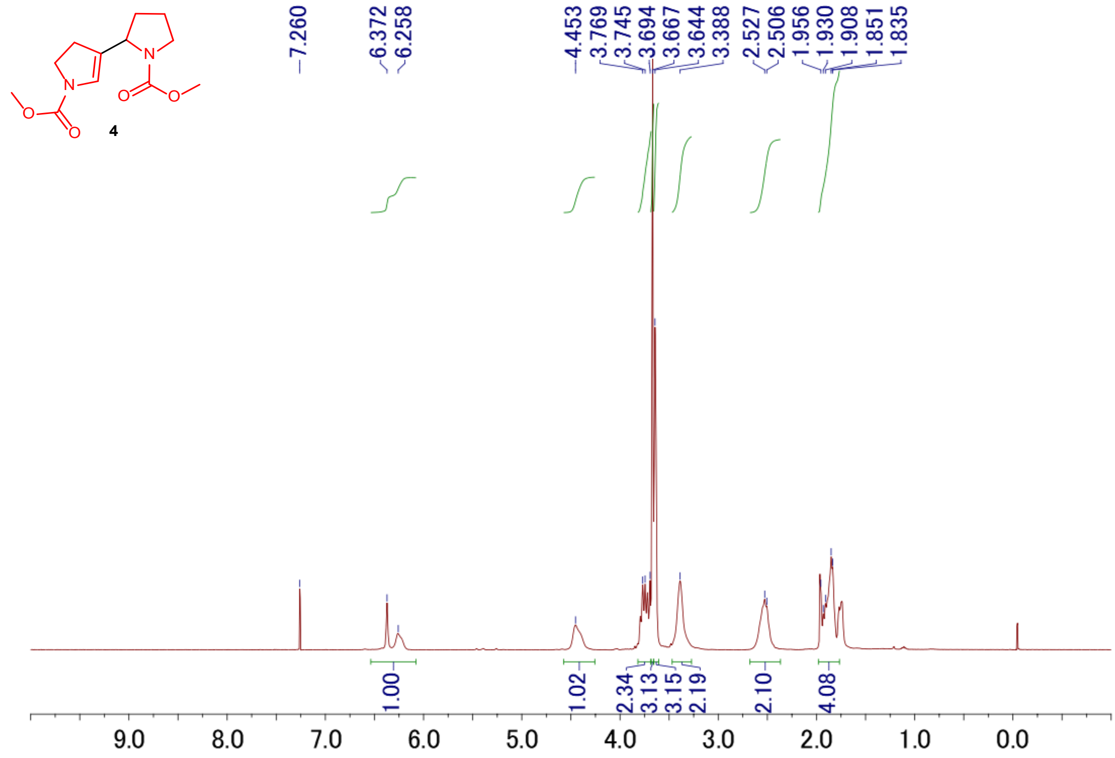

^{1}H NMR spectrum of methyl 4-(*N*-methoxycarbonylpyrrolidine-2-yl)-2,3-dihydropyrrole-1-carboxylate (4) (400 MHz, $CDCl_3$)

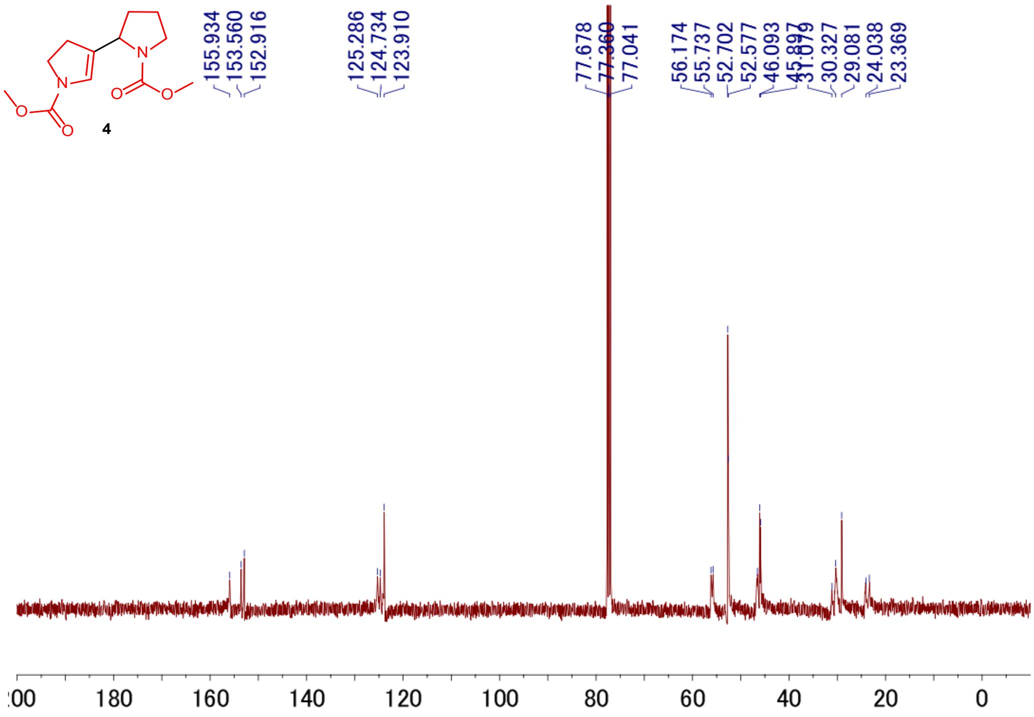

^{13}C NMR spectrum of methyl 4-(*N*-methoxycarbonylpyrrolidine-2-yl)-2,3-dihydropyrrole-1-carboxylate (4) (100 MHz, $CDCl_3$)

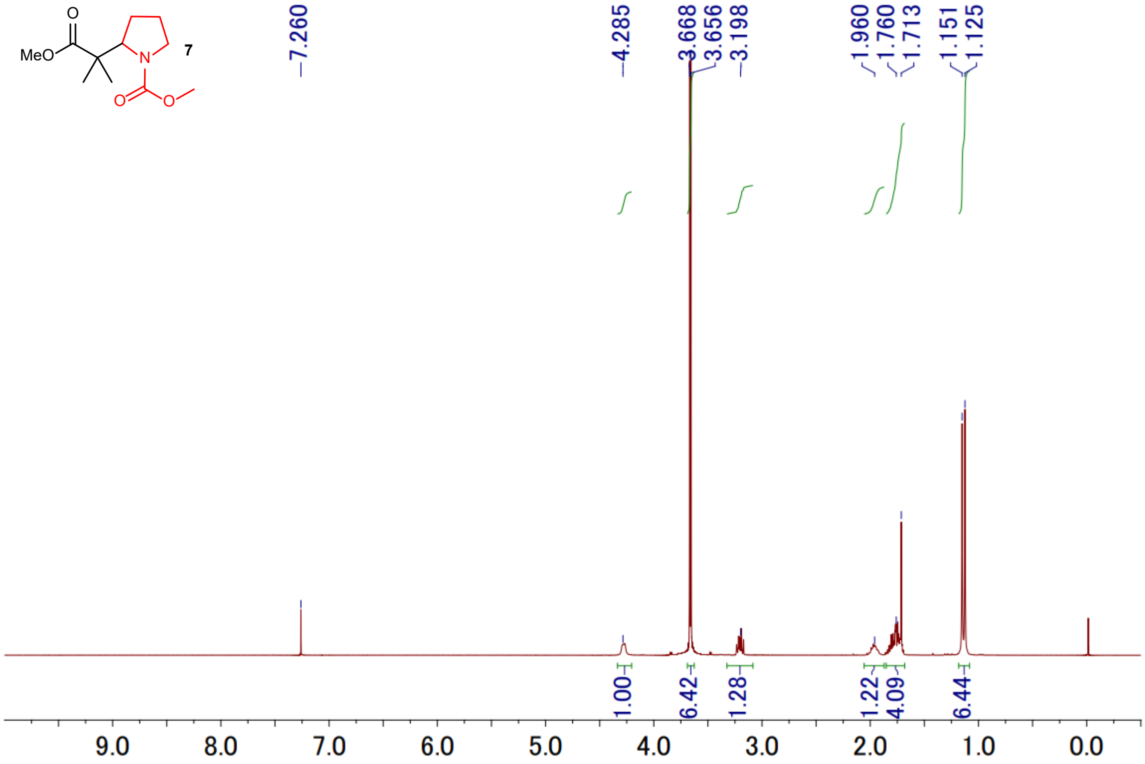

^{1}H NMR spectrum of methyl 2-(1-methoxy-2-methyl-1-oxopropan-2-yl)pyrrolidine-1-carboxylate (7) (400 MHz, CDCl3)

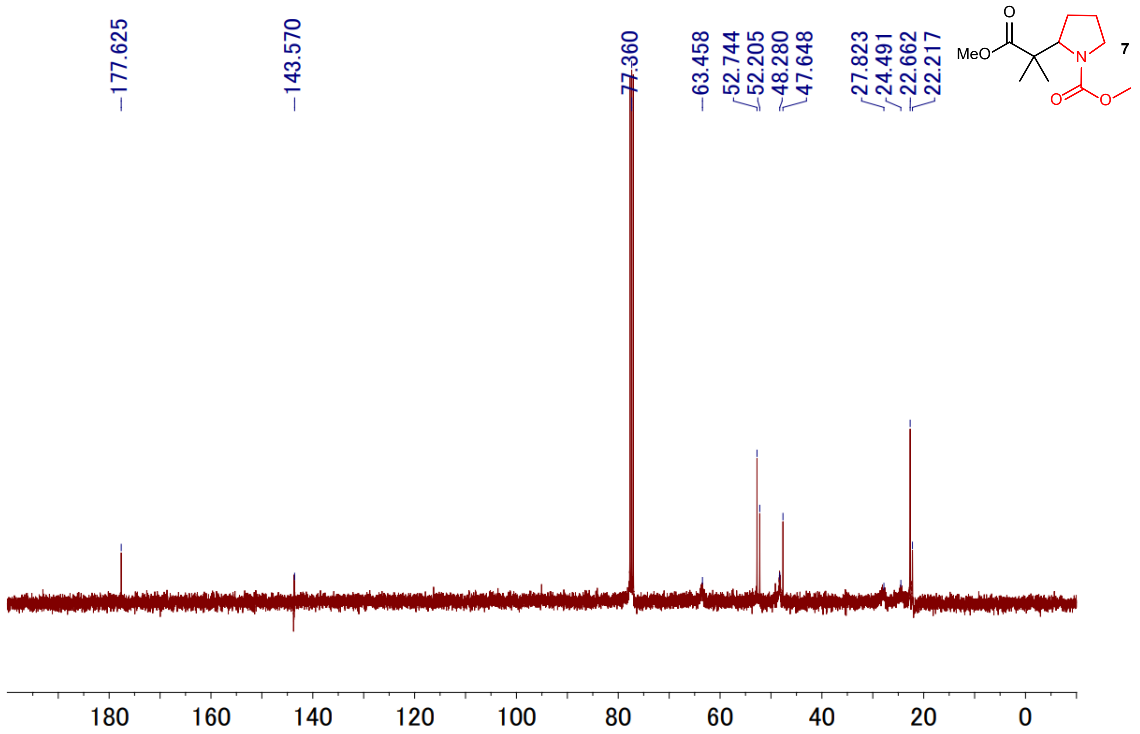

^{13}C NMR spectrum of methyl 2-(1-methoxy-2-methyl-1-oxopropan-2-yl)pyrrolidine-1-carboxylate (7) (100 MHz, CDCl3)

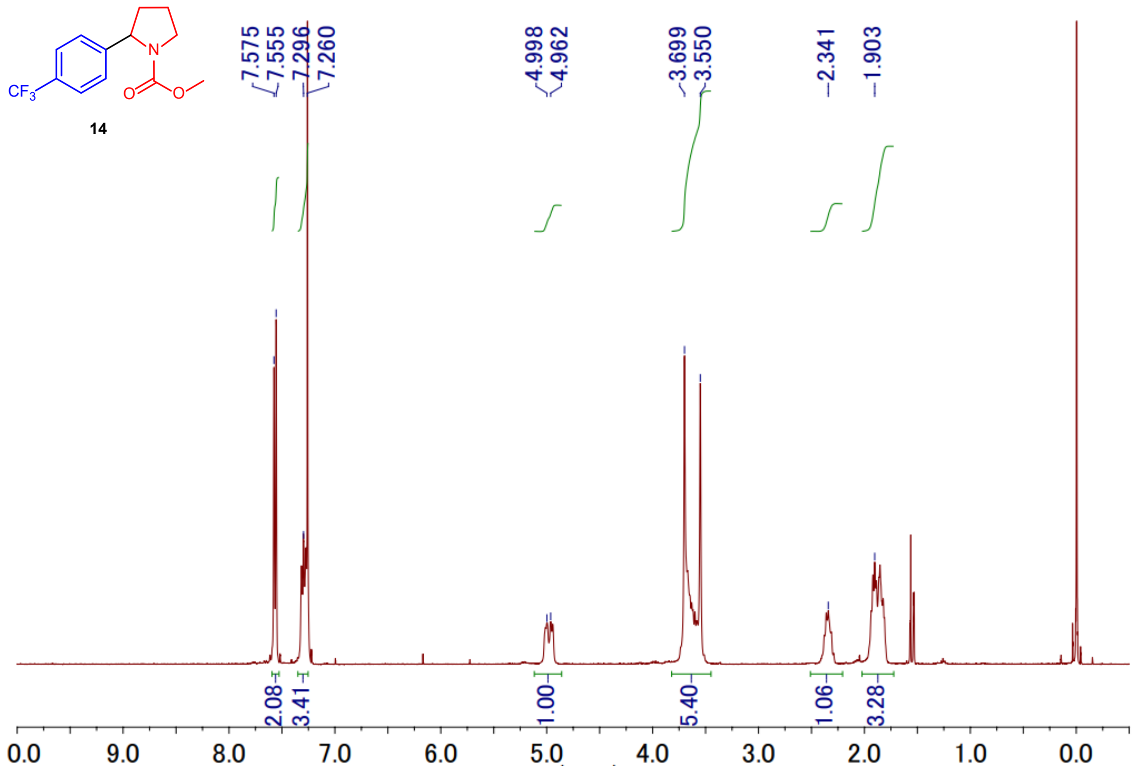

^{1}H NMR of methyl 2-(4-(trifluoromethyl)phenyl)pyrrolidine-1-carboxylate (14) (400 MHz, $CDCl_3$)

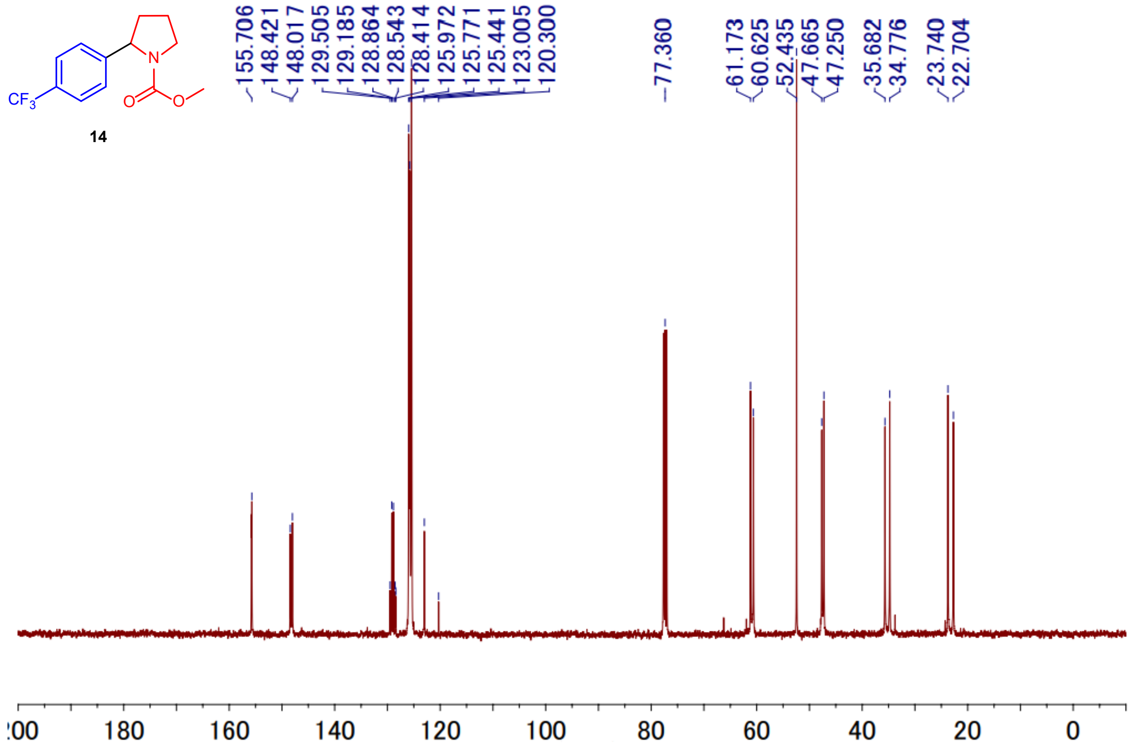

^{13}C NMR spectrum of methyl 2-(4-(trifluoromethyl)phenyl)pyrrolidine-1-carboxylate (14) (100 MHz, $CDCl_3$)

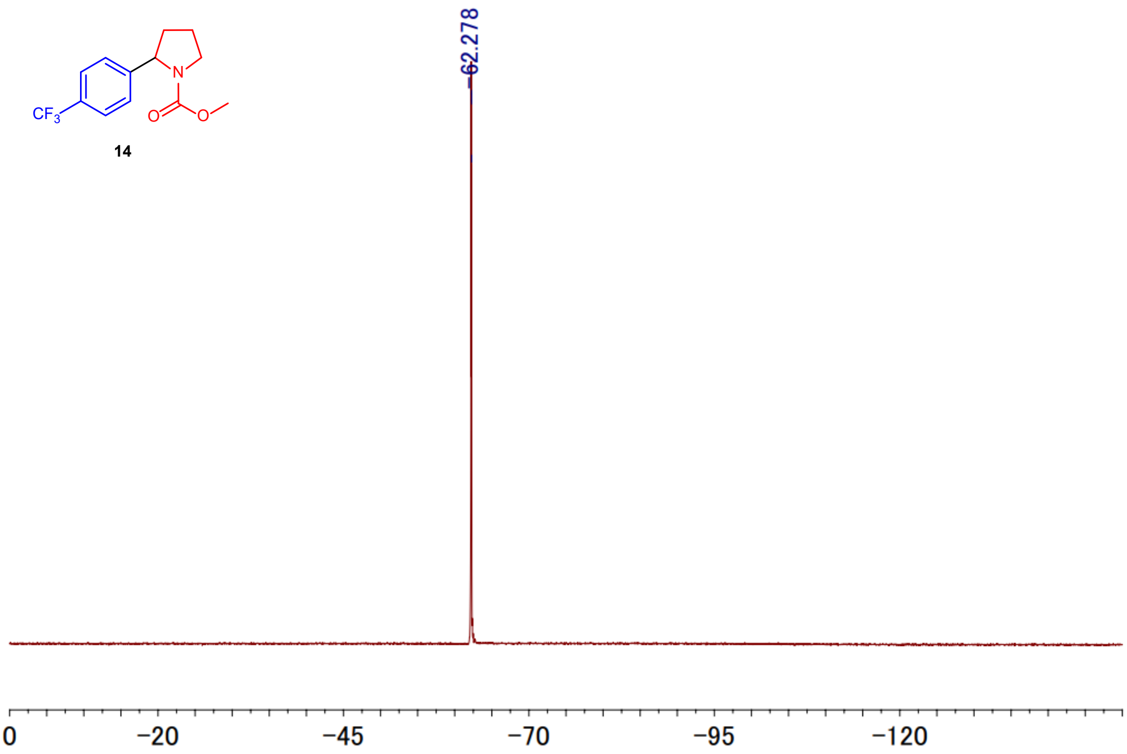

^{19}F NMR spectrum of methyl 2-(4-trifluoromethylphenyl)pyrrolidine-1-carboxylate (14) (376 MHz, $CDCl_3$)

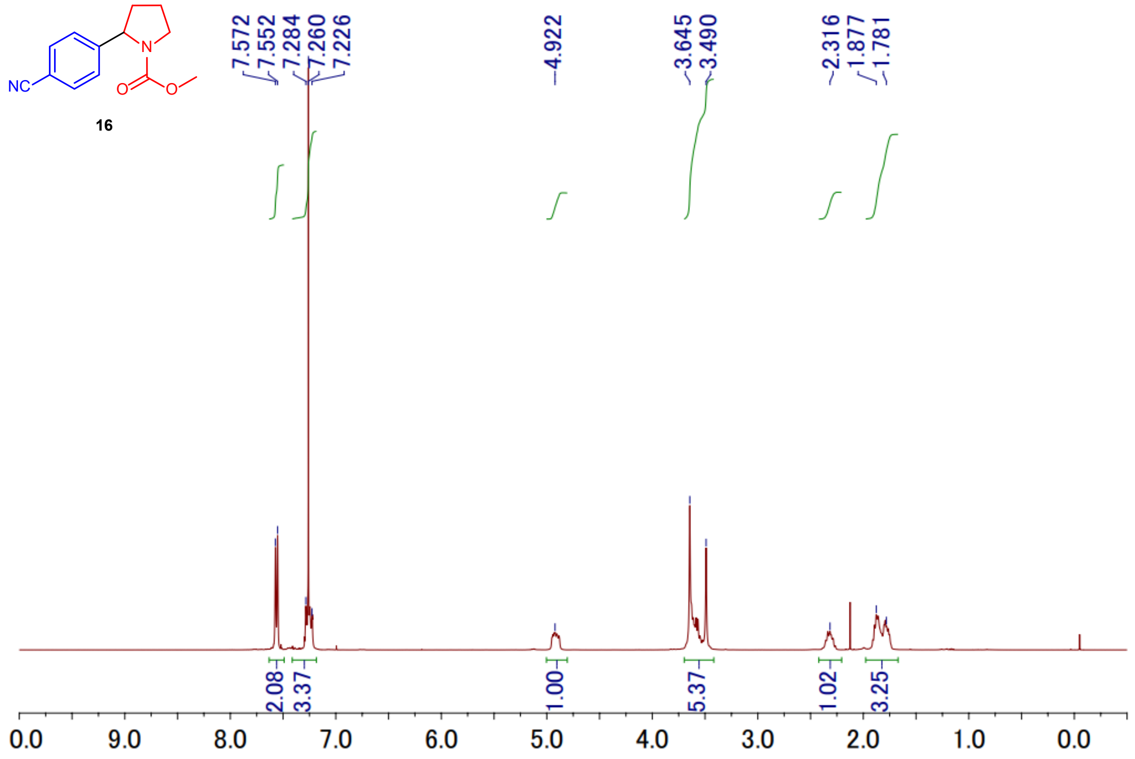

^{1}H NMR spectrum of methyl 2-(4-cyanophenyl)pyrrolidine-1-carboxylate (16) (400 MHz, $CDCl_3$)

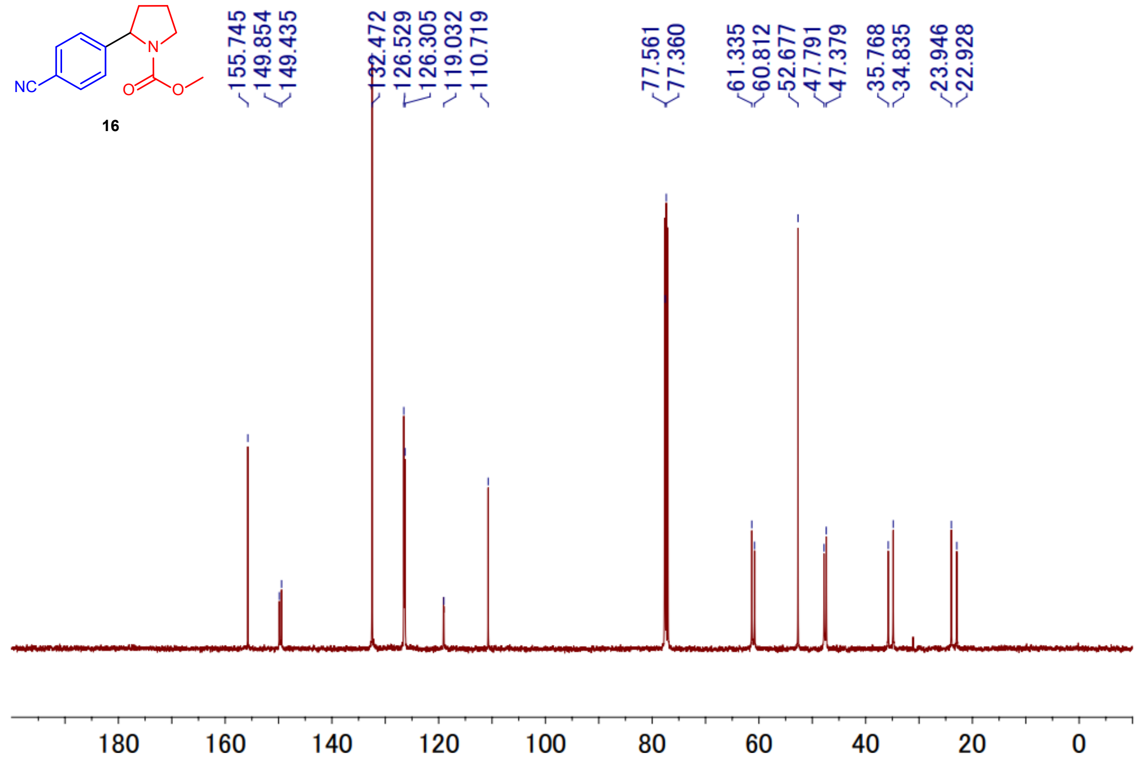

^{13}C NMR spectrum of methyl 2-(4-cyanophenyl)pyrrolidine-1-carboxylate (16) (100 MHz, $CDCl_3$)

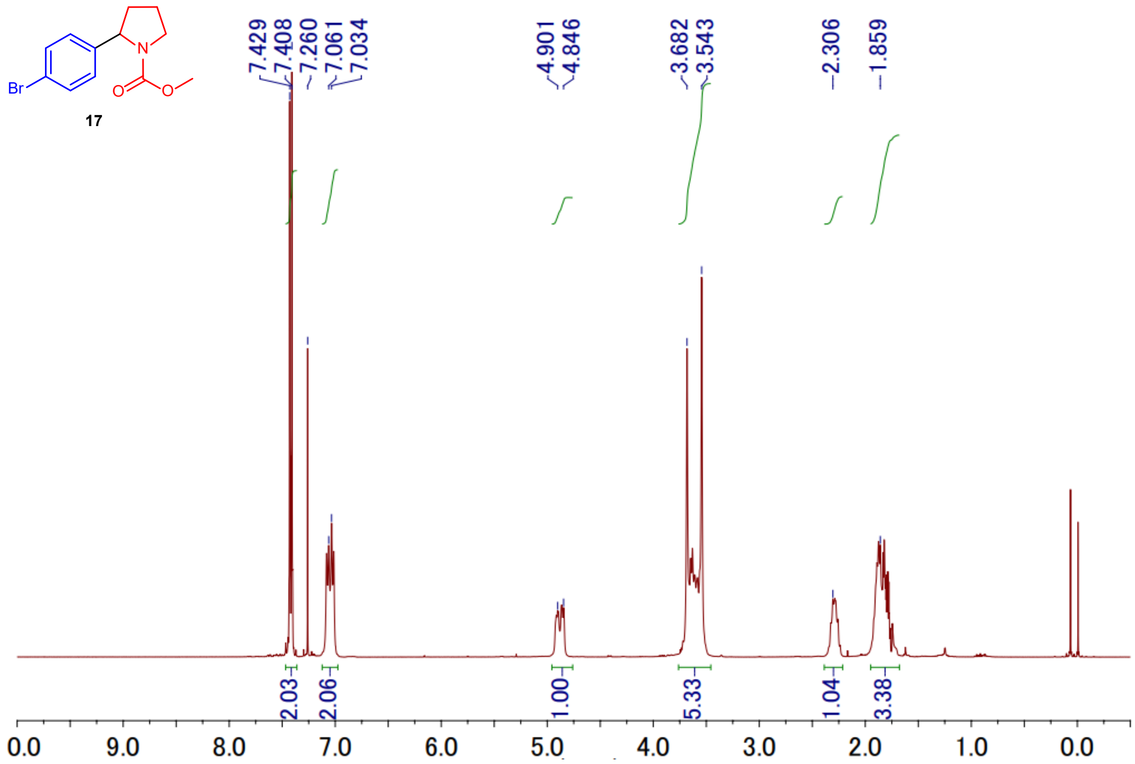

^{1}H NMR spectrum of methyl 2-(4-bromophenyl)pyrrolidine-1-carboxylate (17) (400 MHz, $CDCl_3$)

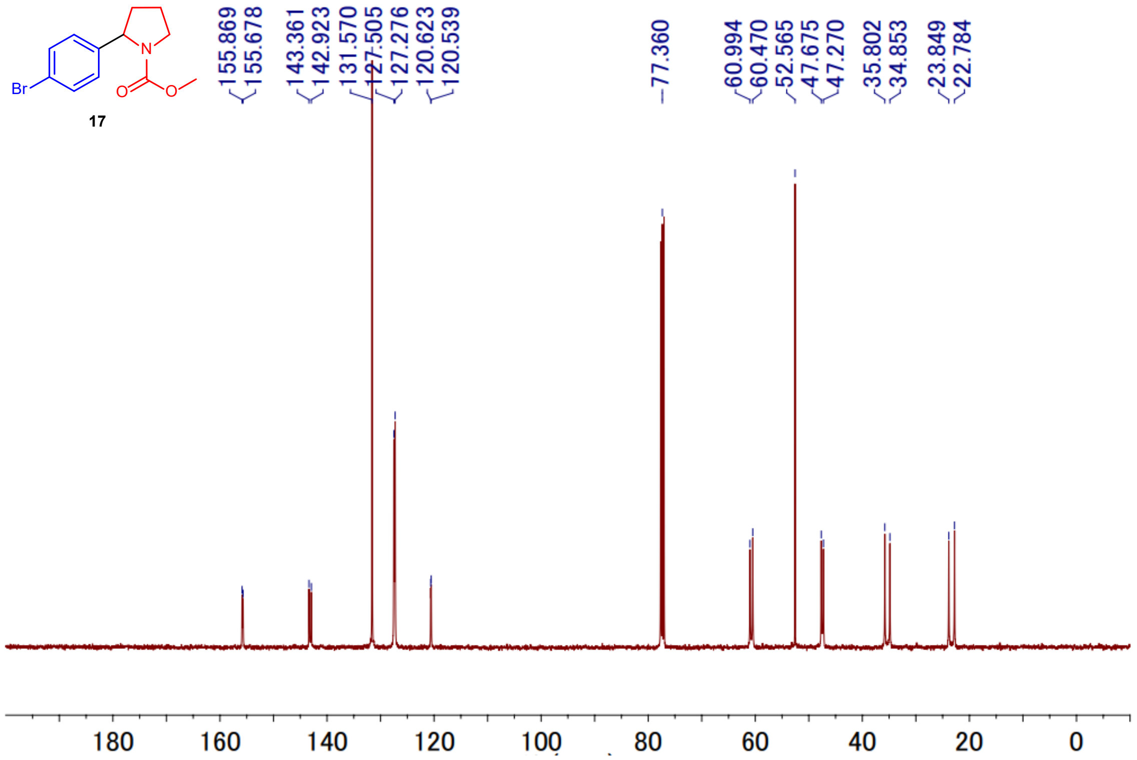

^{13}C NMR spectrum of methyl 2-(4-bromophenyl)pyrrolidine-1-carboxylate (17) (100 MHz, $CDCl_3$)

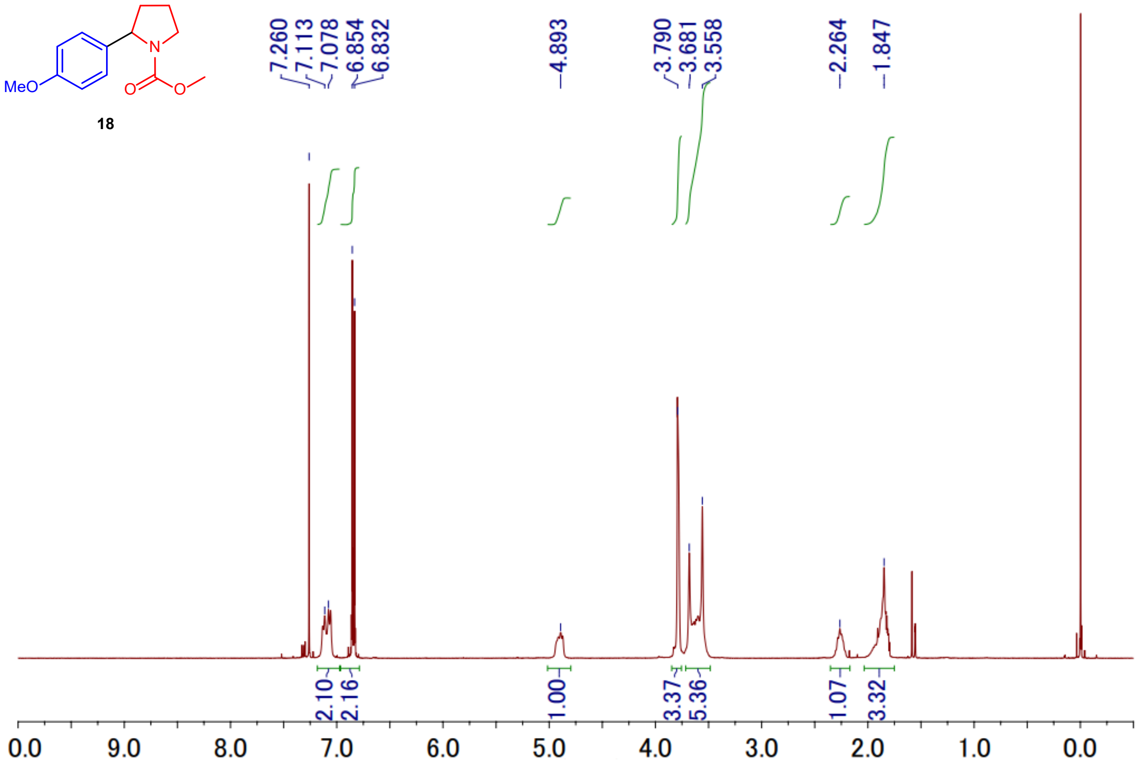

^{1}H NMR spectrum of methyl 2-(4-methoxyphenyl)pyrrolidine-1-carboxylate (18) (400 MHz, $CDCl_3$)

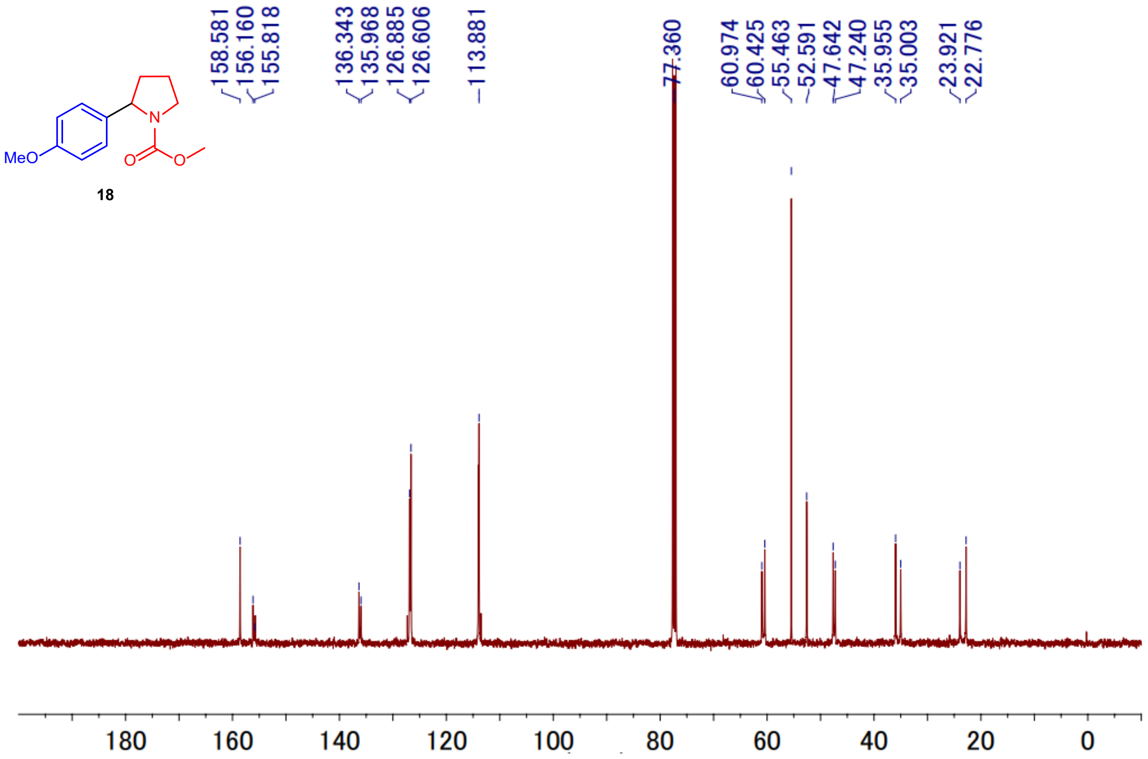

^{13}C NMR spectrum of methyl 2-(4-methoxyphenyl)pyrrolidine-1-carboxylate (18) (100 MHz, $CDCl_3$)

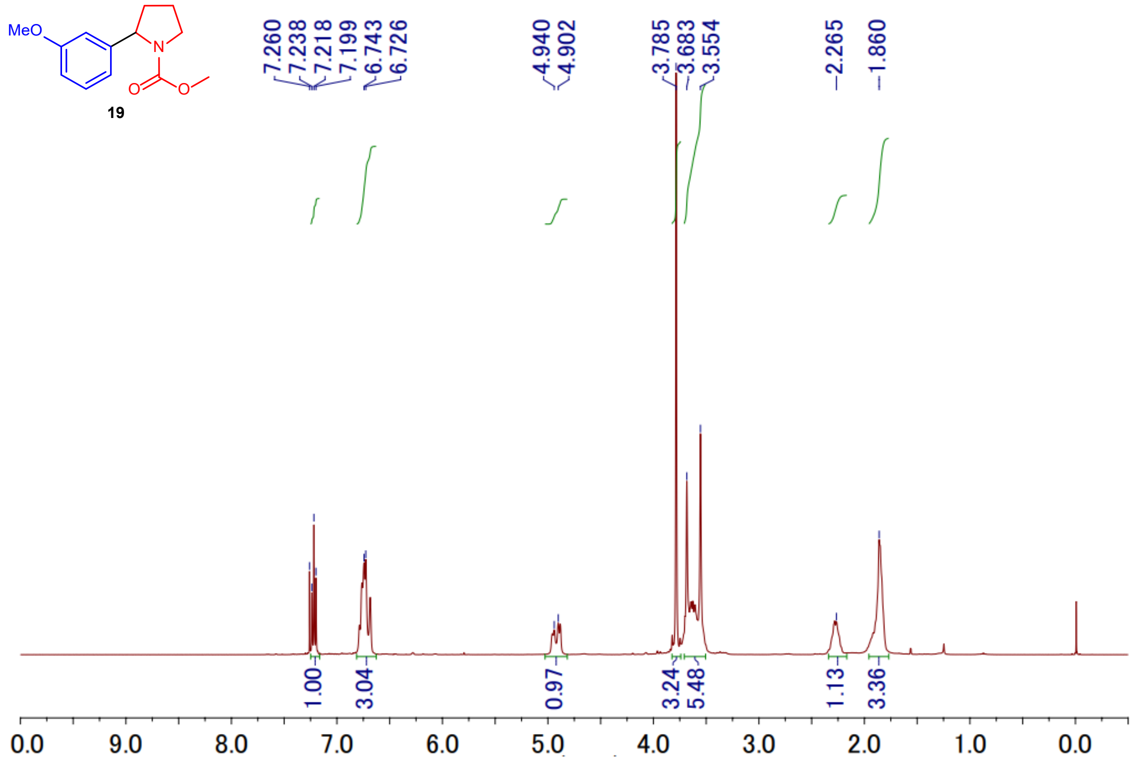

^{1}H NMR spectrum of methyl 2-(3-methoxyphenyl)pyrrolidine-1-carboxylate (19) (400 MHz, $CDCl_3$)

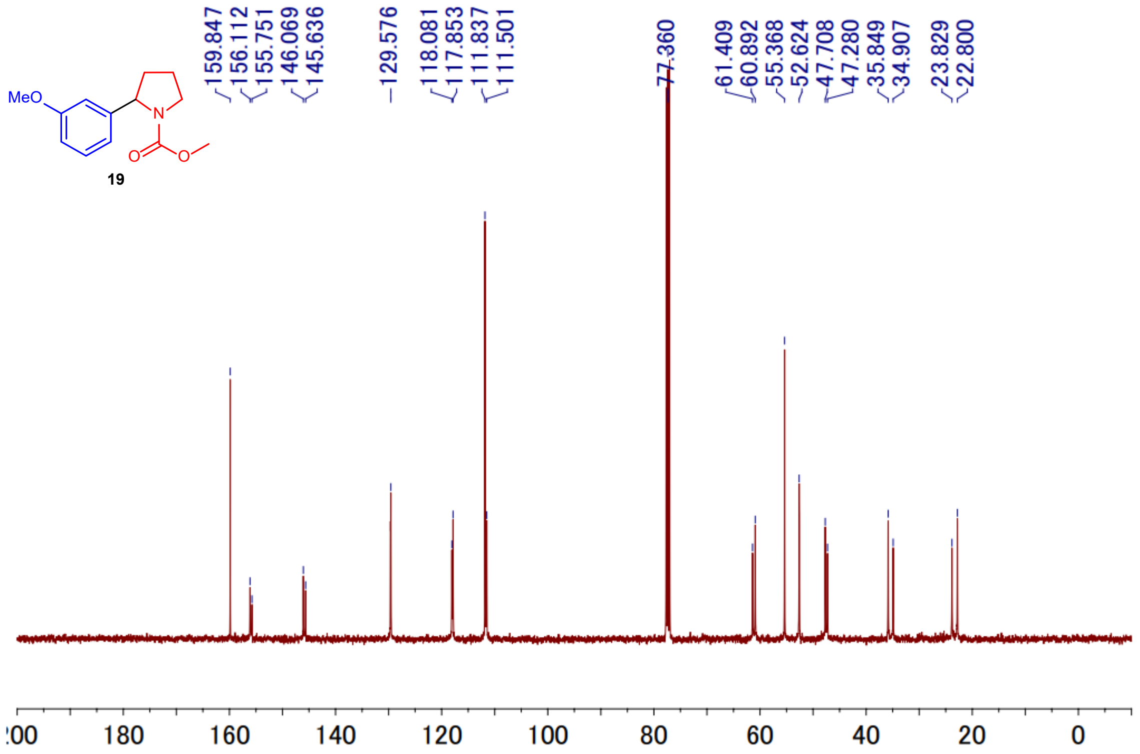

^{13}C NMR spectrum of methyl 2-(3-methoxyphenyl)pyrrolidine-1-carboxylate (19) (100 MHz, $CDCl_3$)

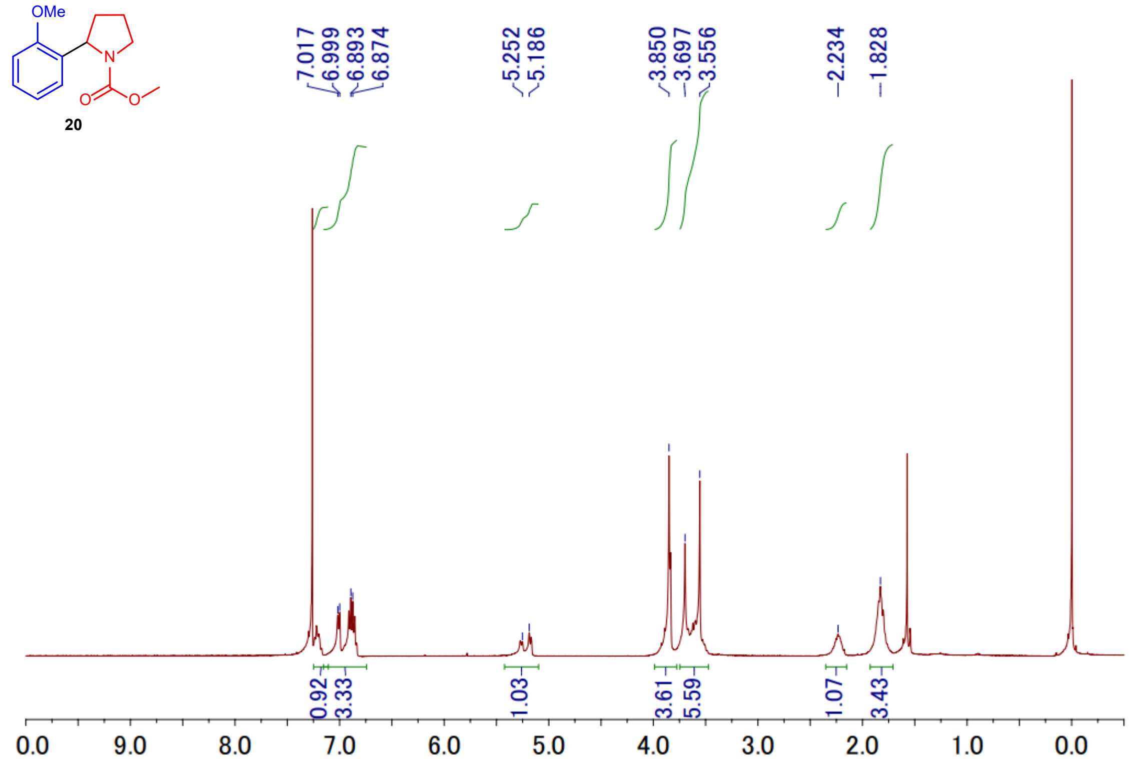

^{1}H NMR spectrum of methyl 2-(2-methoxyphenyl)pyrrolidine-1-carboxylate (20) (400 MHz, $CDCl_3$)

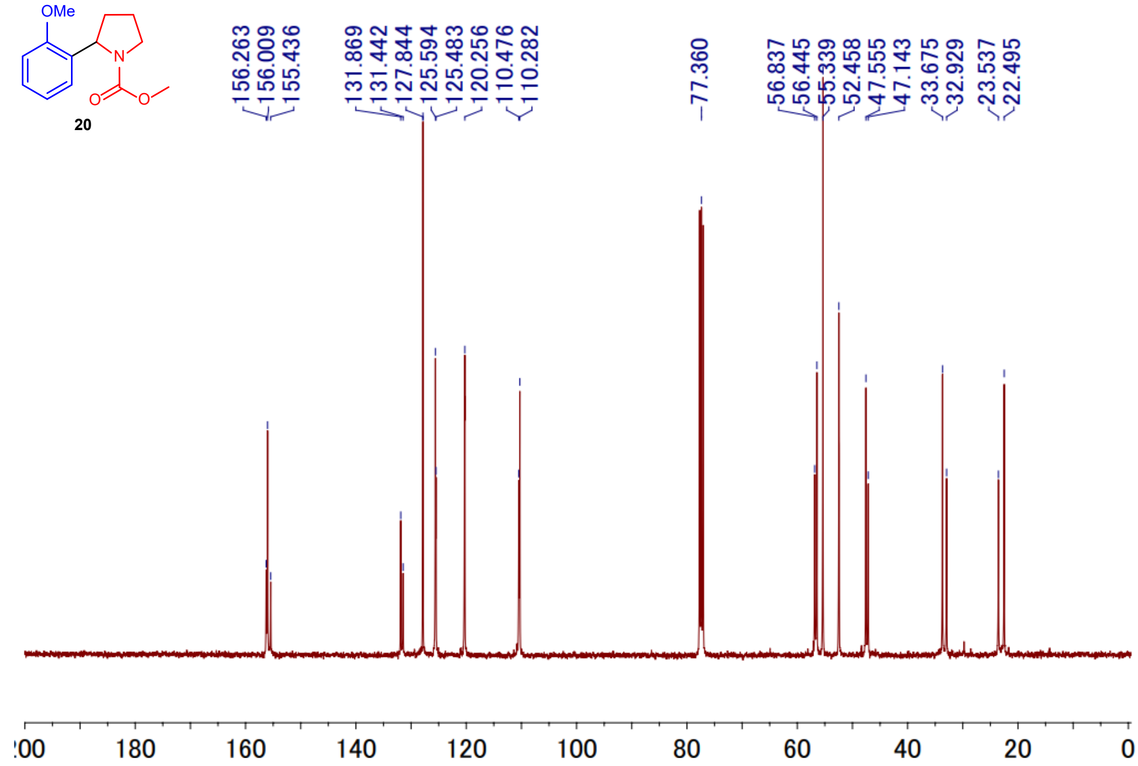

^{13}C NMR spectrum of methyl 2-(2-methoxyphenyl)pyrrolidine-1-carboxylate (20) (100 MHz, $CDCl_3$)

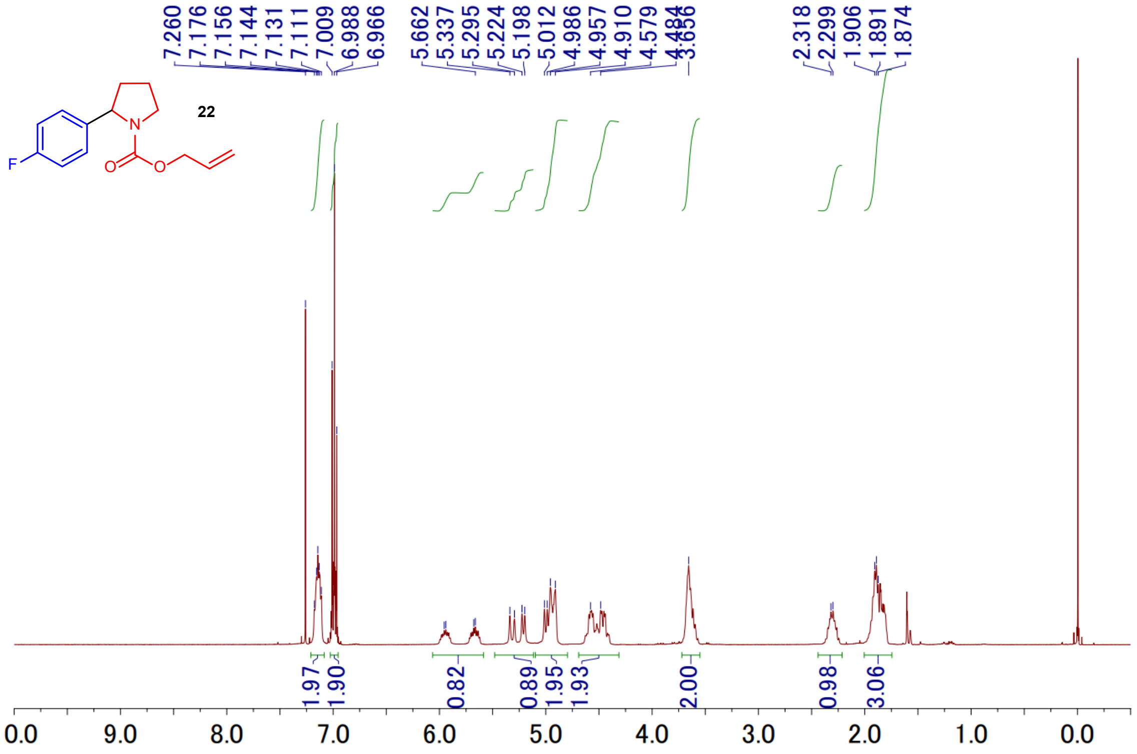

^{1}H NMR spectrum of allyl 2-(4-fluorophenyl)pyrrolidine-1-carboxylate (22) (400 MHz, $CDCl_3$)

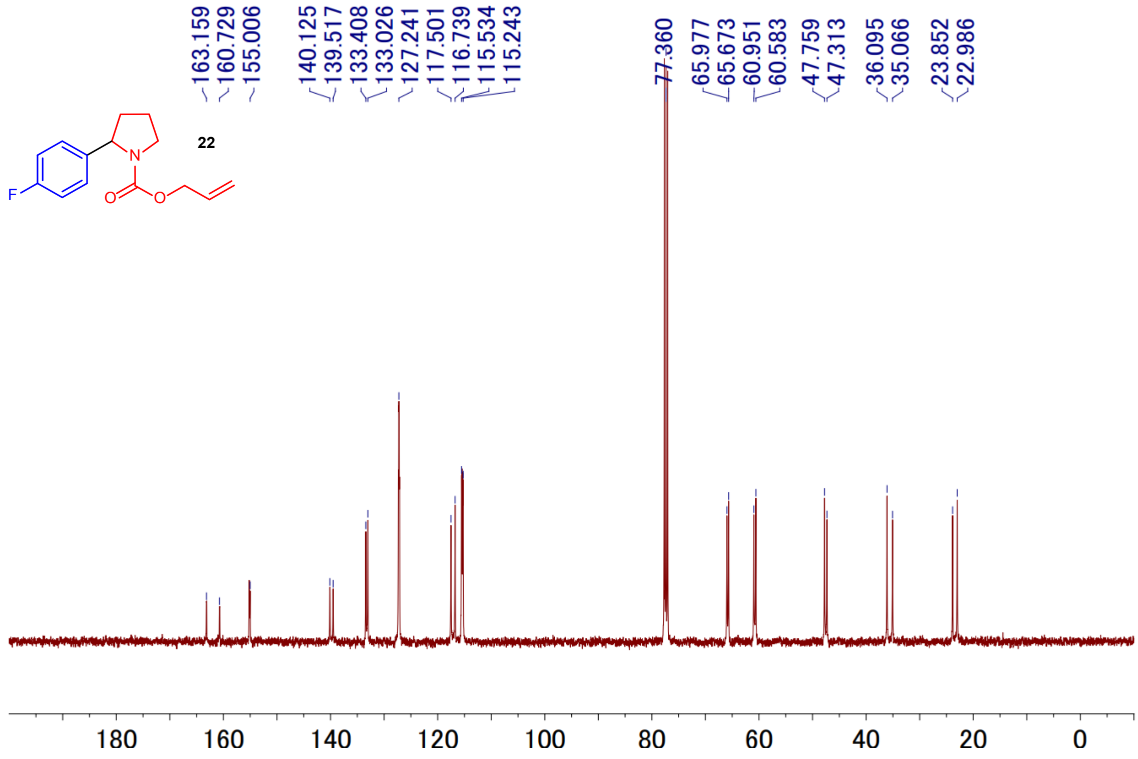

^{13}C NMR spectrum of allyl 2-(4-fluorophenyl)pyrrolidine-1-carboxylate (22) (100 MHz, $CDCl_3$)

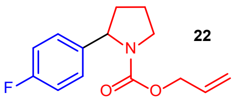

22

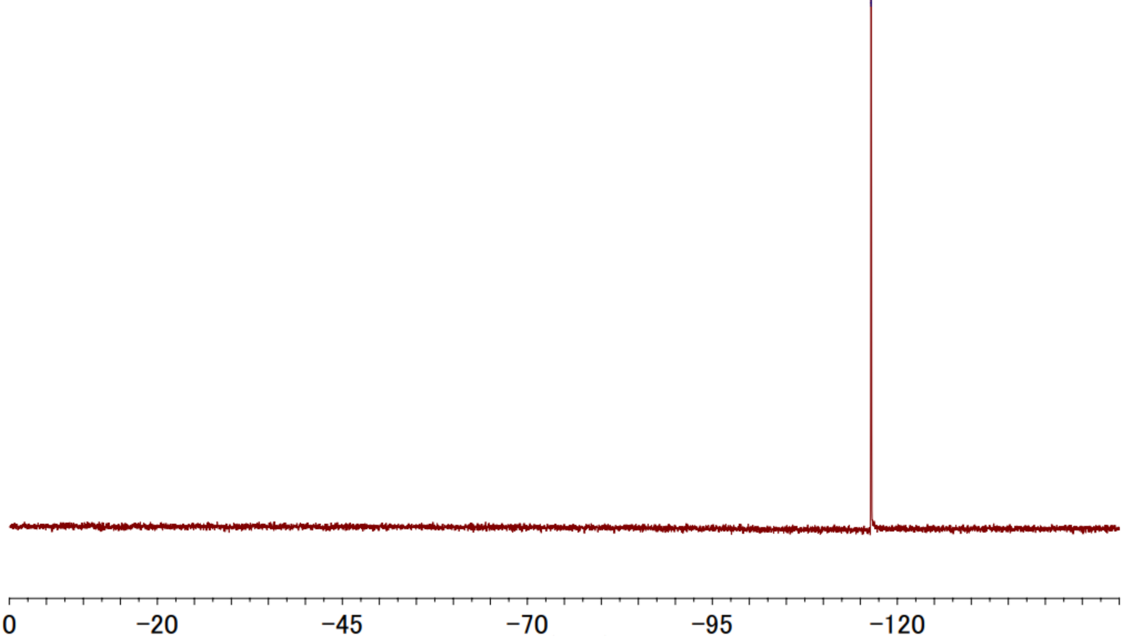

^{19}F NMR spectrum of allyl 2-(4-fluorophenyl)pyrrolidine-1-carboxylate (22) (376 MHz, CDCl₃)

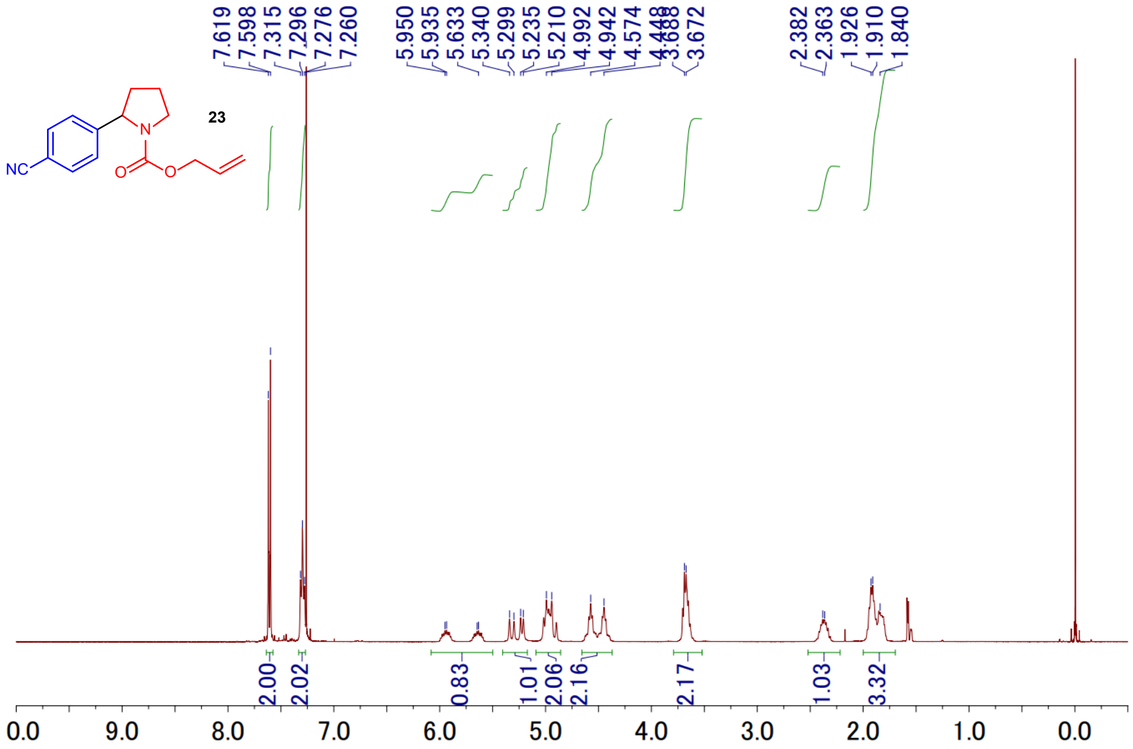

^{1}H NMR spectrum of allyl 2-(4-cyanophenyl)pyrrolidine-1-carboxylate (23) (400 MHz, CDCl$_3$)

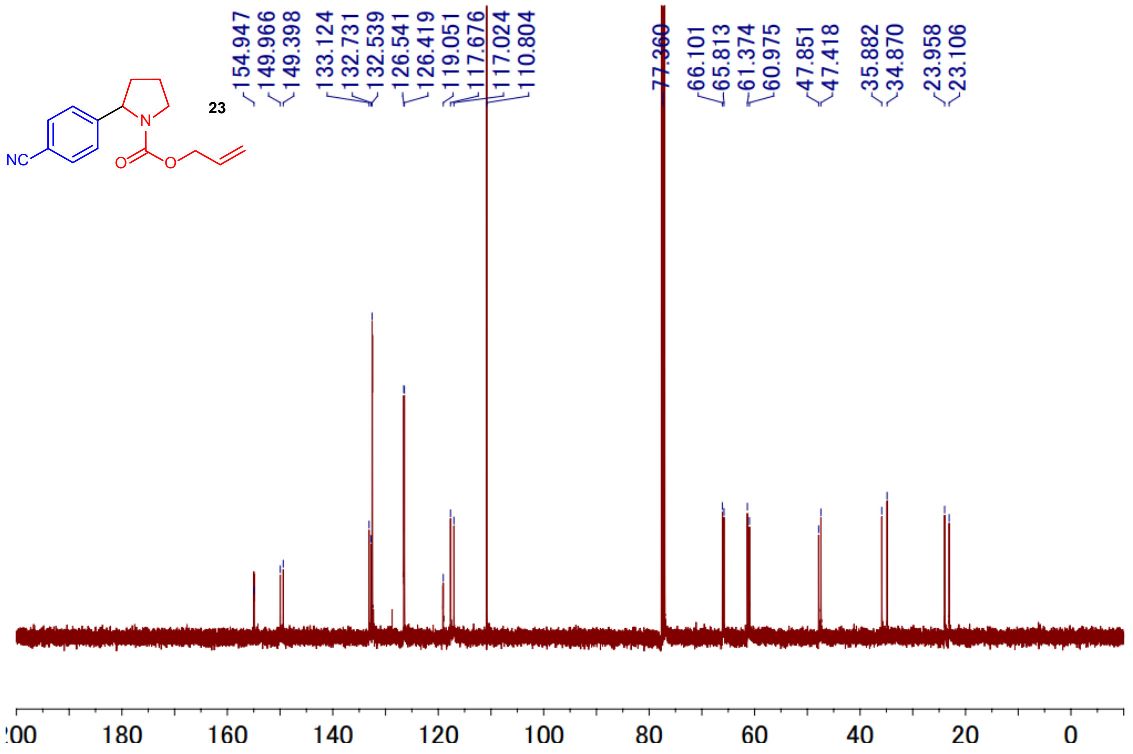

^{13}C NMR spectrum of allyl 2-(4-cyanophenyl)pyrrolidine-1-carboxylate (23) (100 MHz, CDCl$_3$)

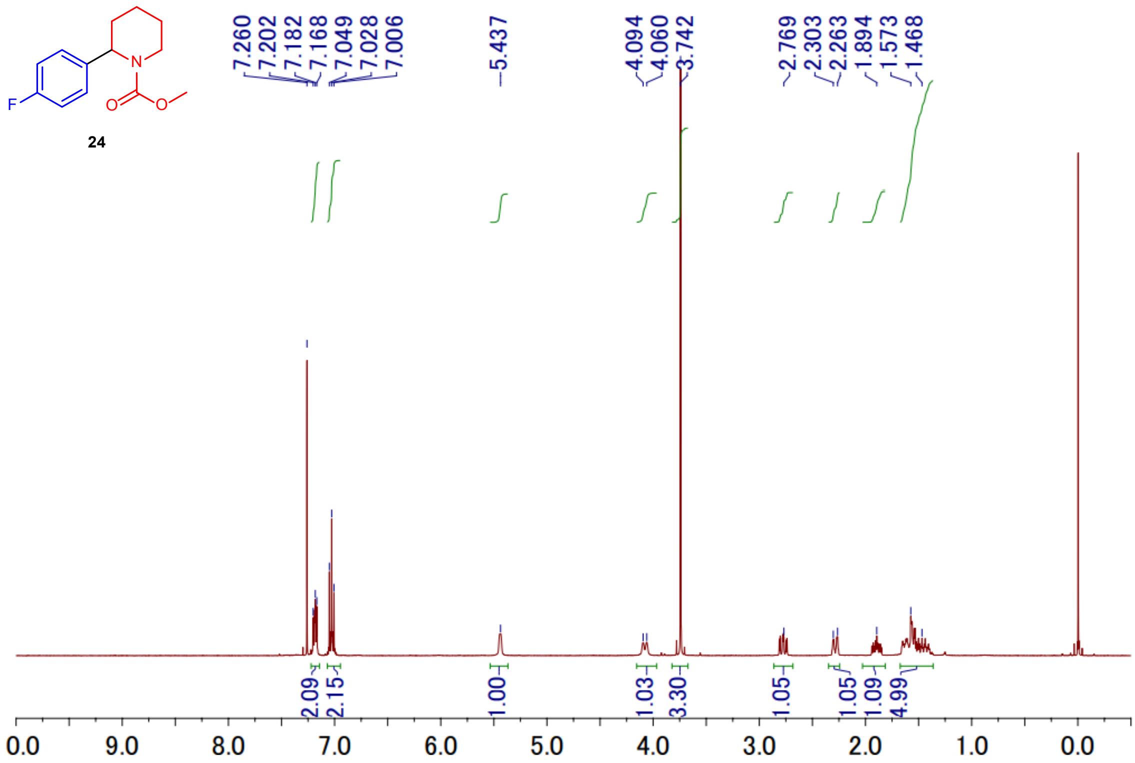

^{1}H NMR spectrum of methyl 2-(4-fluorophenyl)piperidine-1-carboxylate (24) (400 MHz, $CDCl_3$)

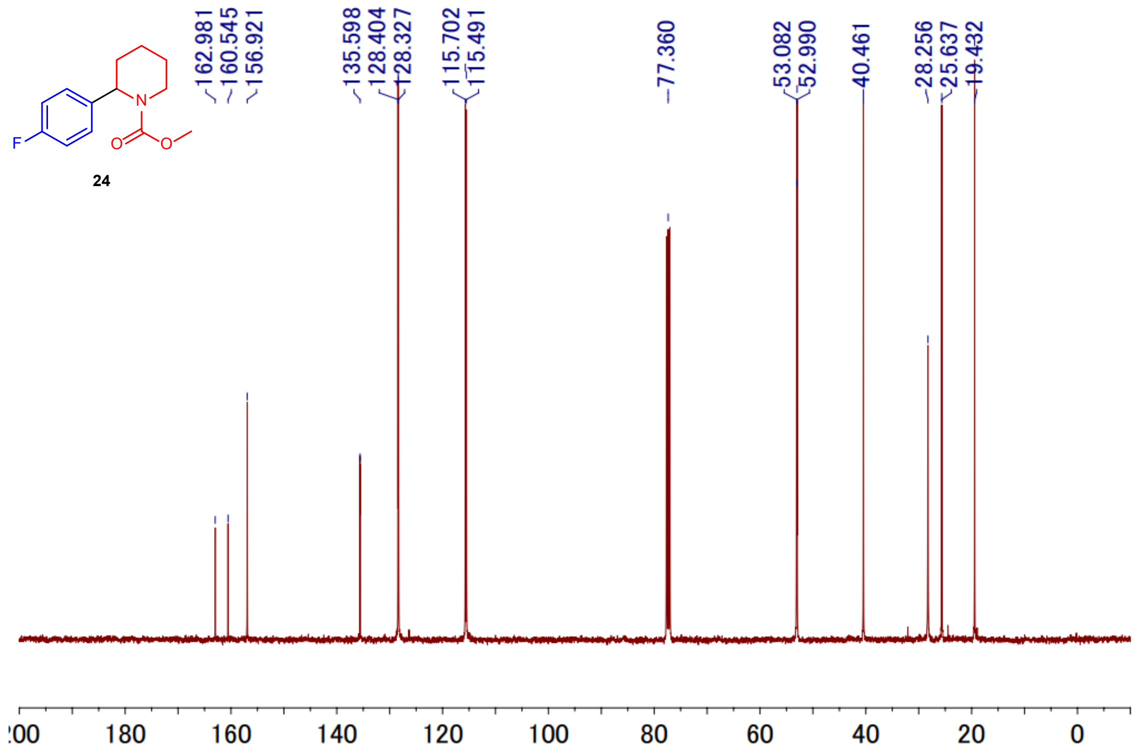

^{13}C NMR spectrum of methyl 2-(4-fluorophenyl)piperidine-1-carboxylate (24) (100 MHz, $CDCl_3$)

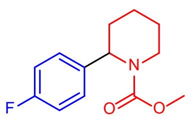

24

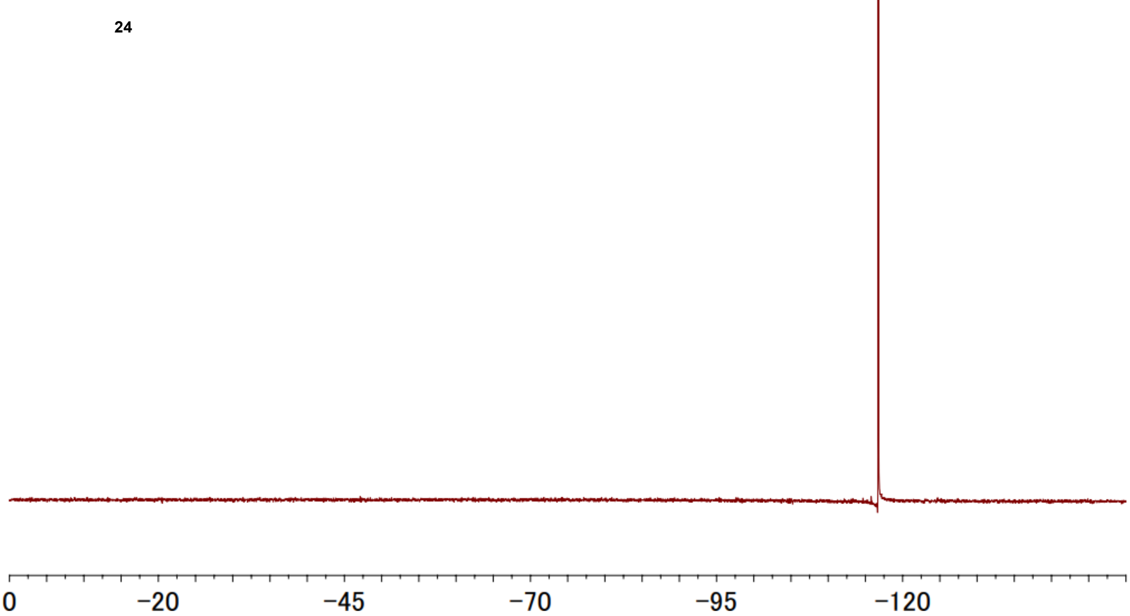

^{19}F NMR spectrum of methyl 2-(4-fluorophenyl)piperidine-1-carboxylate (24) (376 MHz, $CDCl_3$)

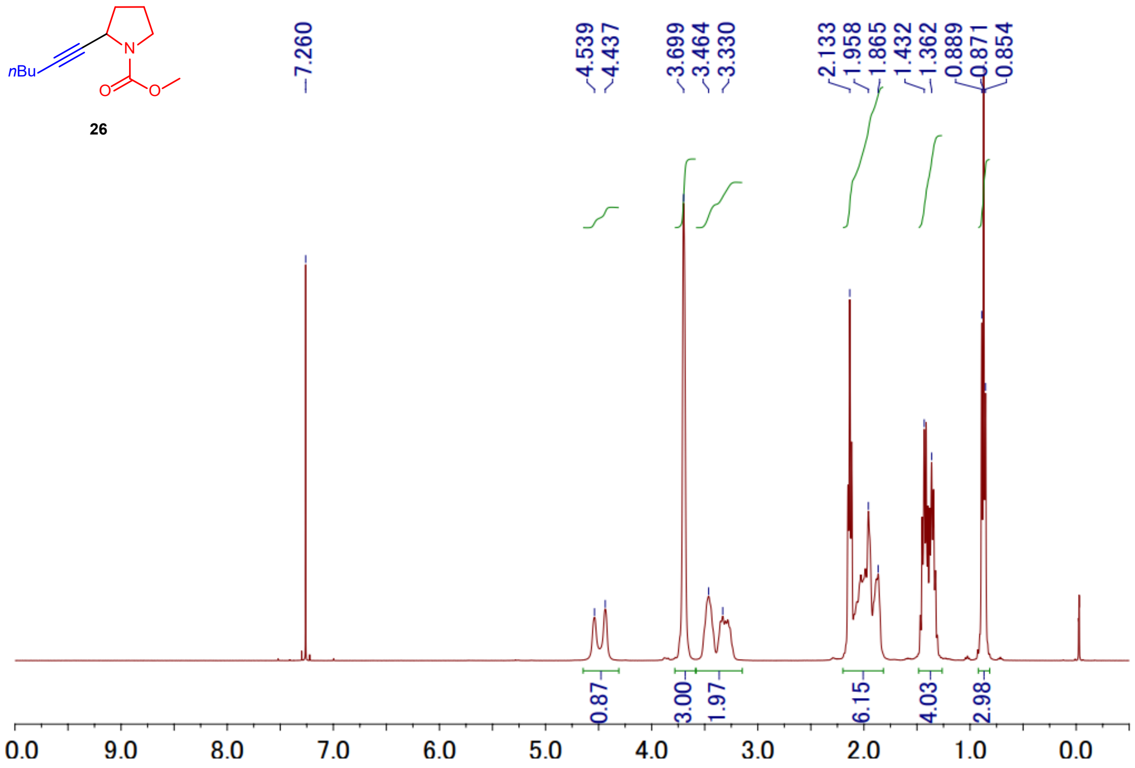

^{1}H NMR spectrum of methyl 2-(1-hexyn-1-yl)pyrrolidine-1-carboxylate (26) (400 MHz, $CDCl_3$)

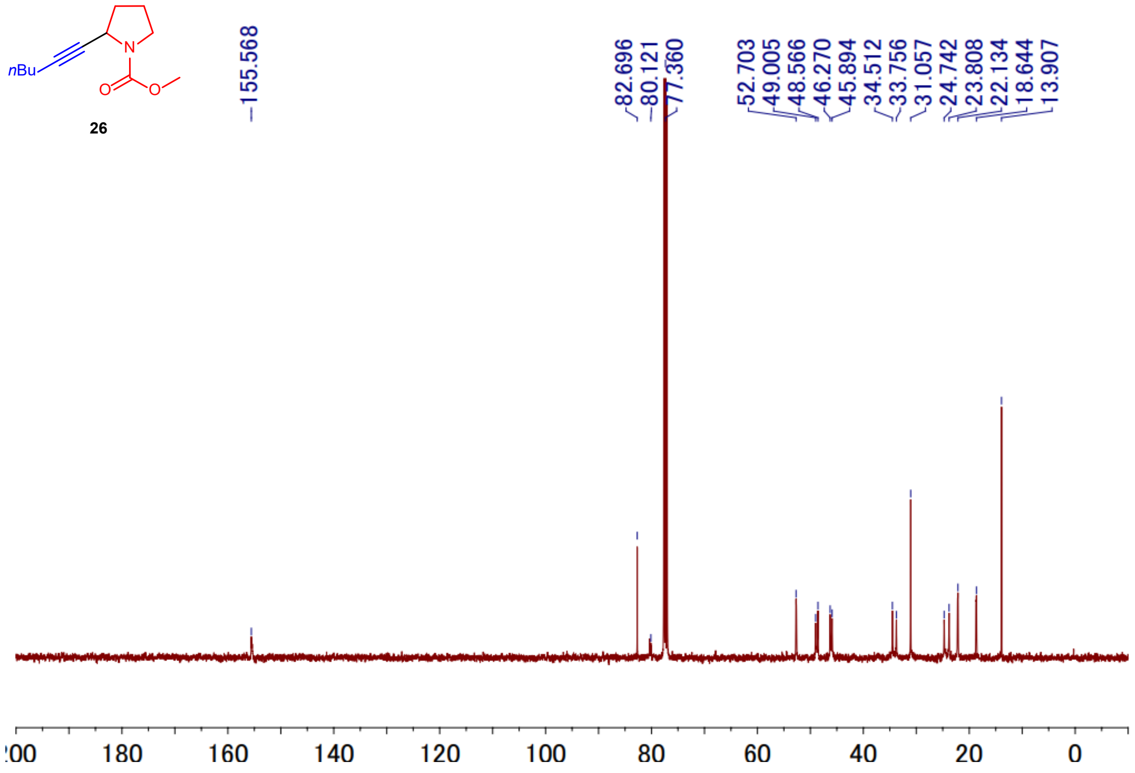

^{13}C NMR spectrum of methyl 2-(1-hexyn-1-yl)pyrrolidine-1-carboxylate (26) (100 MHz, $CDCl_3$)

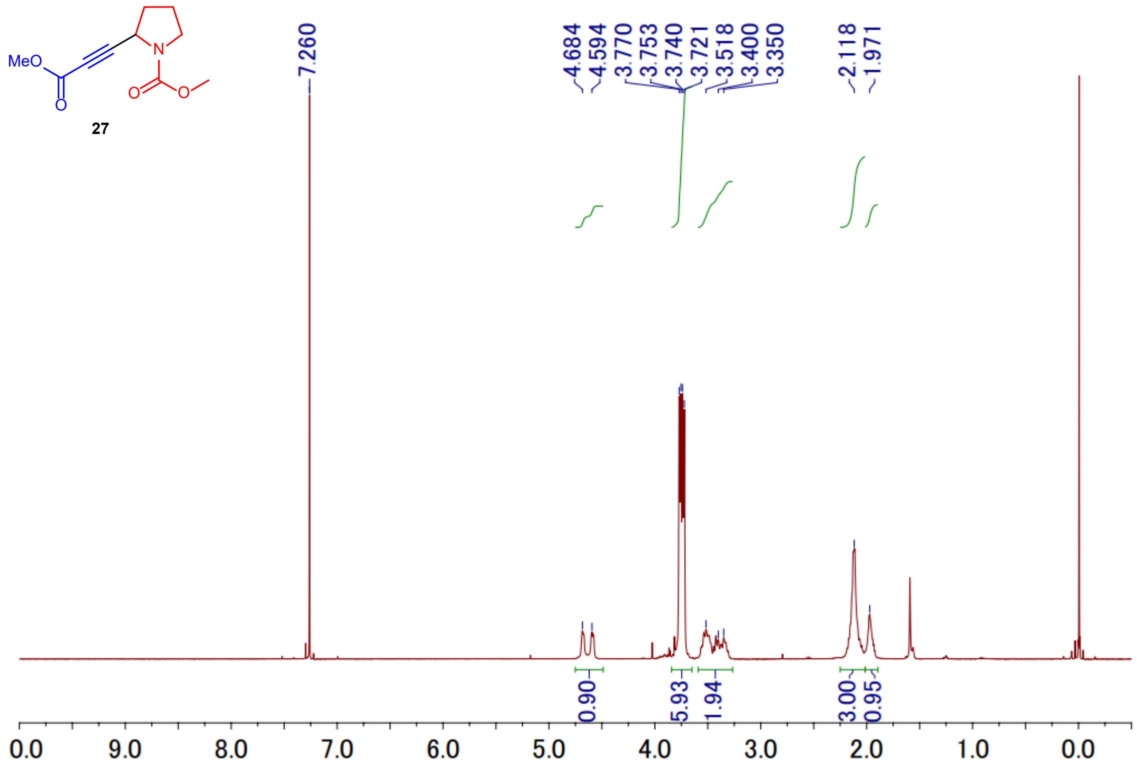

^{1}H NMR spectrum of methyl 2-(3-methoxy-3-oxopropynyl)pyrrolidine-1-carboxylate (27) (400 MHz, $CDCl_3$)

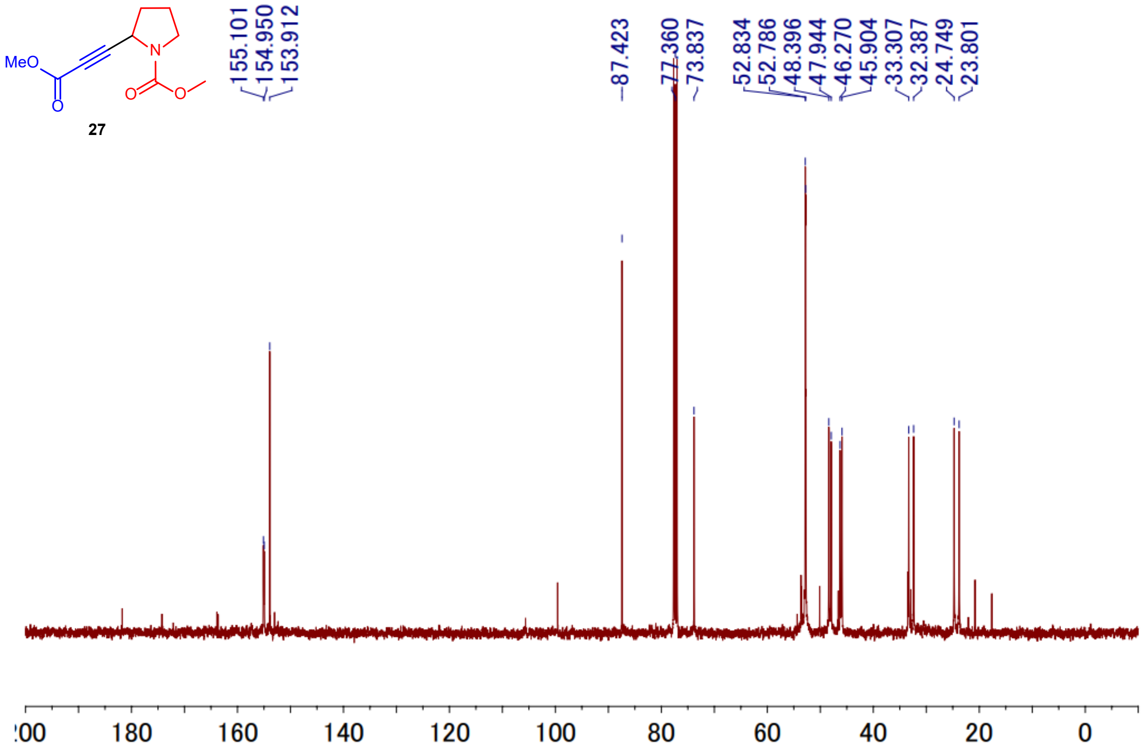

^{13}C NMR spectrum of methyl 2-(3-methoxy-3-oxopropynyl)pyrrolidine-1-carboxylate (27) (100 MHz, $CDCl_3$)

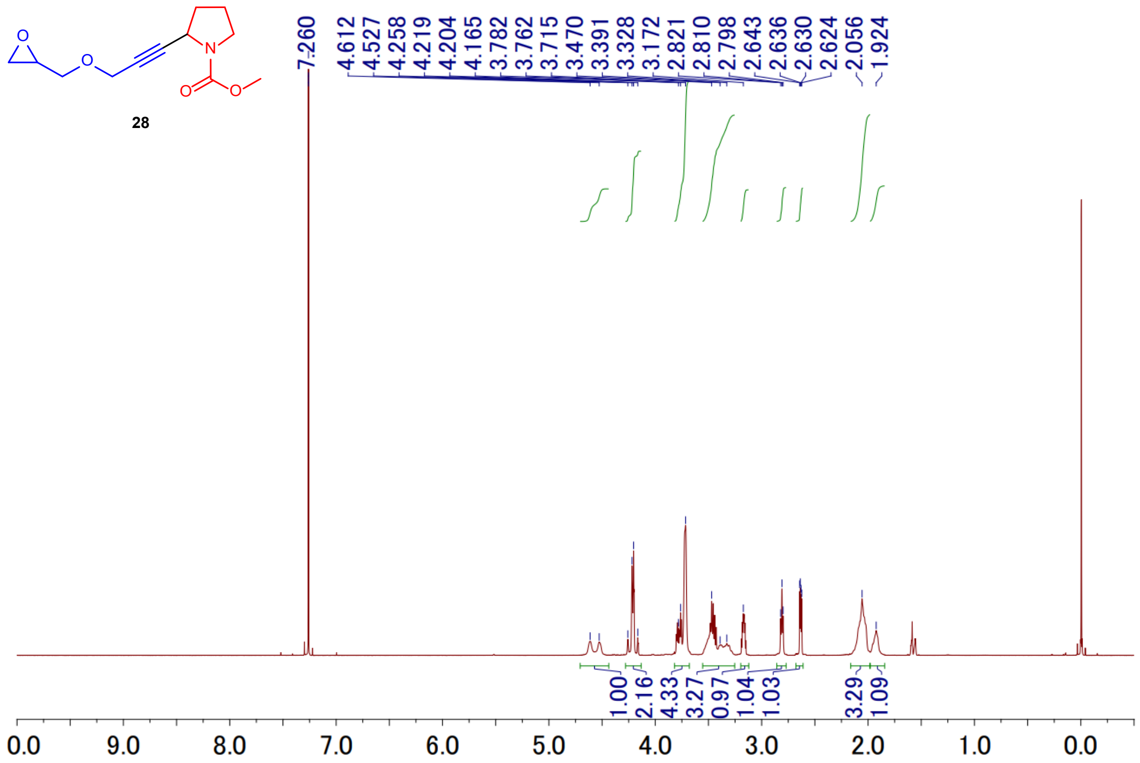

^{1}H NMR spectrum of methyl 2-(3-glycidyloxy-1-propynyl)pyrrolidine-1-carboxylate (28) (400 MHz, $CDCl_3$)

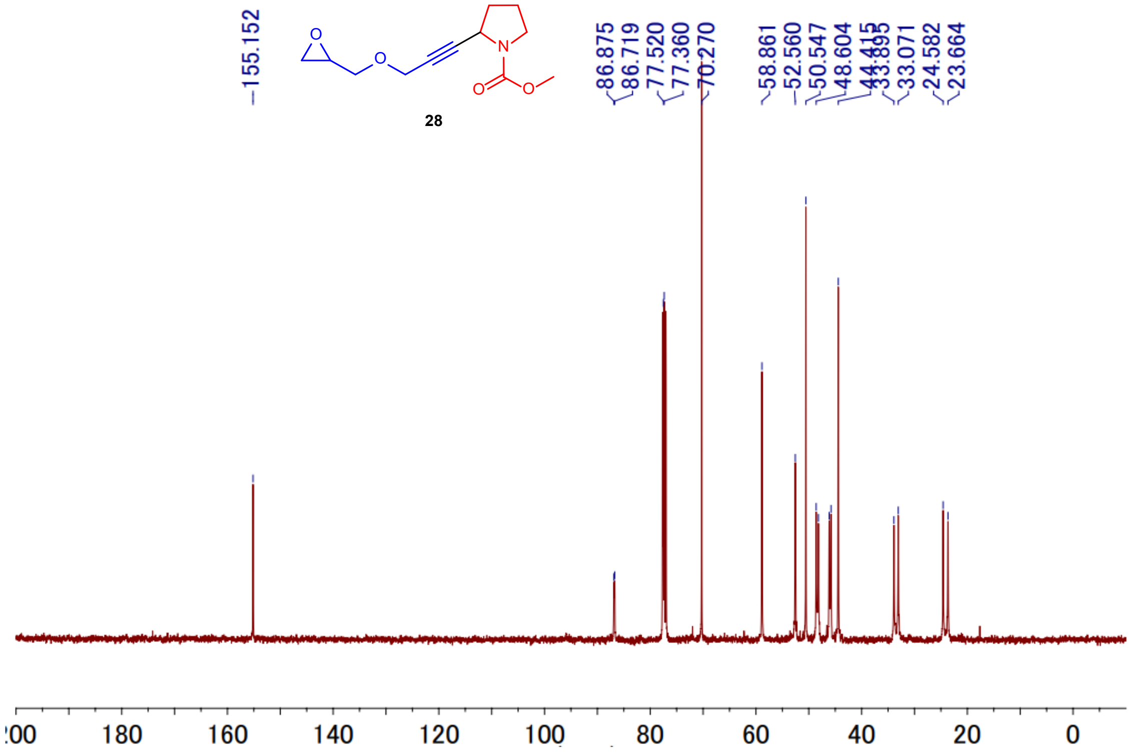

^{13}C NMR spectrum of methyl 2-(3-glycidyloxy-1-propynyl)pyrrolidine-1-carboxylate (28) (100 MHz, $CDCl_3$)

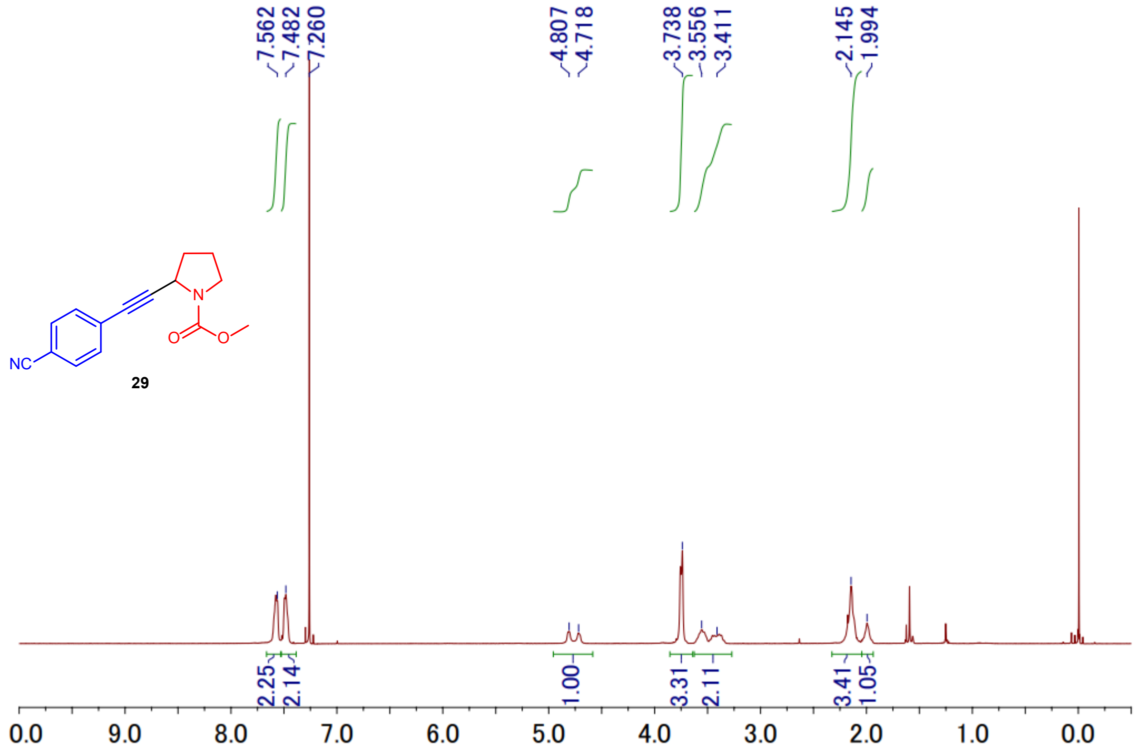

^{1}H NMR spectrum of methyl 2-(4-cyanophenylethynyl)pyrrolidine-1-carboxylate (29) (400 MHz, $CDCl_3$)

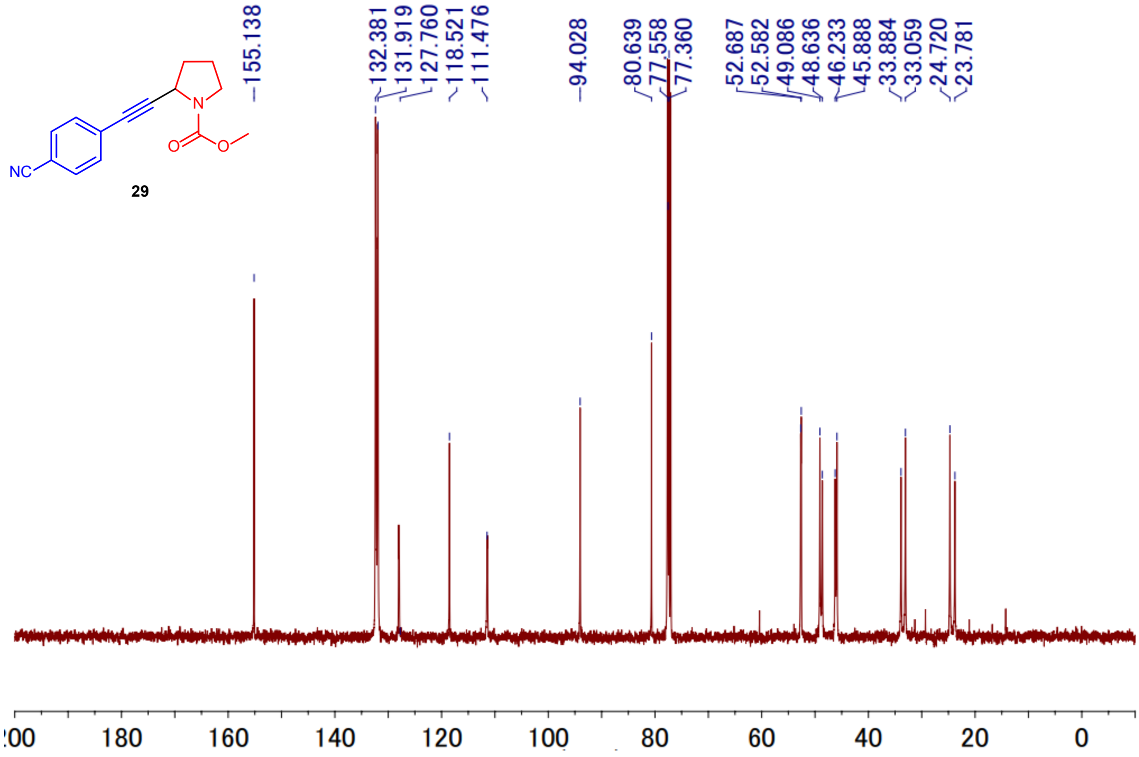

^{13}C NMR spectrum of methyl 2-(4-cyanophenylethynyl)pyrrolidine-1-carboxylate (29) (100 MHz, $CDCl_3$)

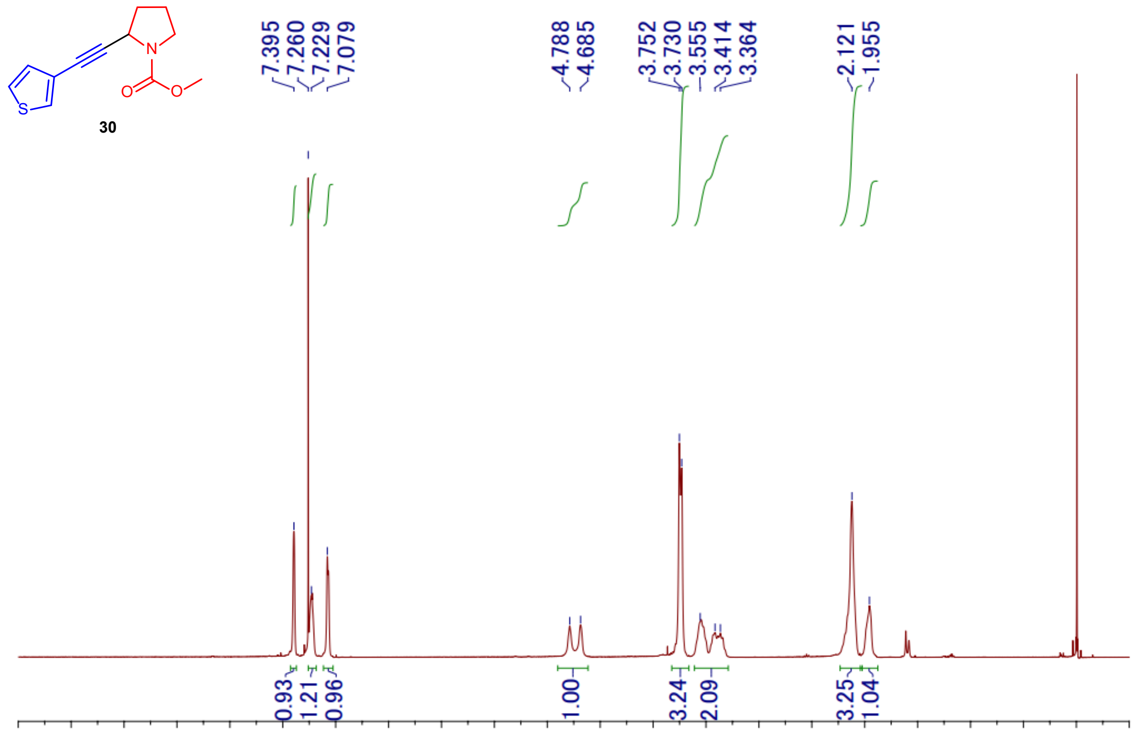

^{1}H NMR spectrum of methyl 2-(thiophen-3-ylethynyl)pyrrolidine-1-carboxylate (30) (400 MHz, $CDCl_3$)

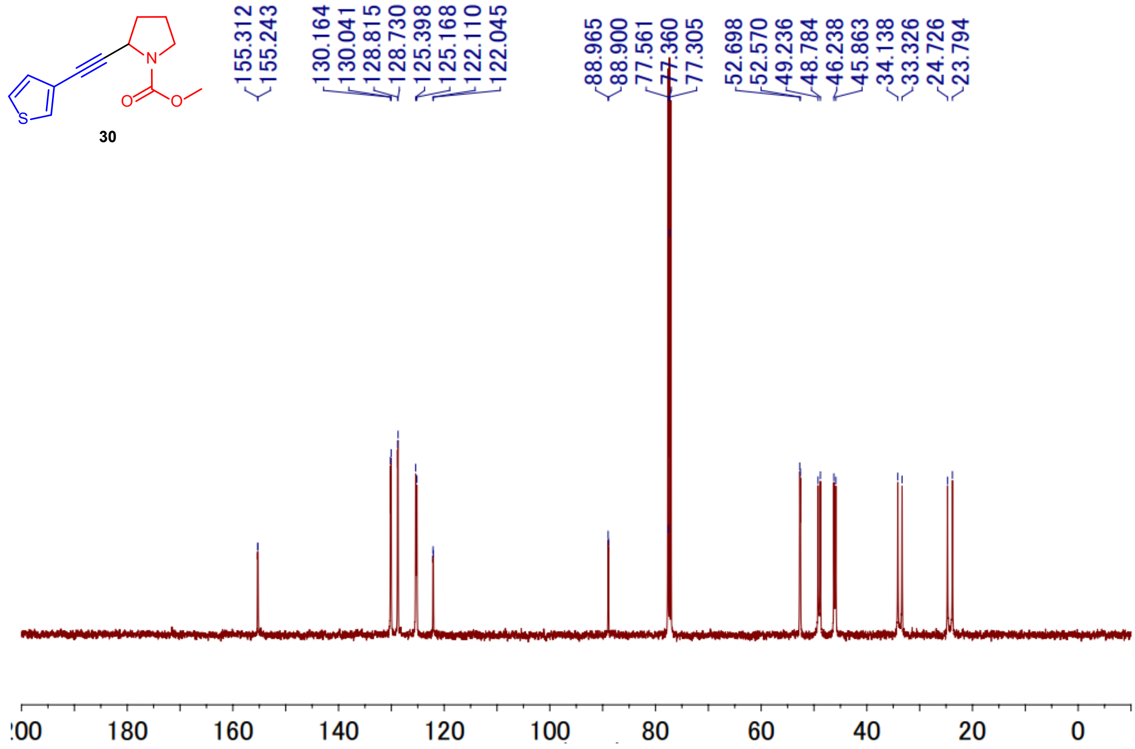

^{13}C NMR spectrum of methyl 2-(thiophen-3-ylethynyl)pyrrolidine-1-carboxylate (30) (100 MHz, $CDCl_3$)

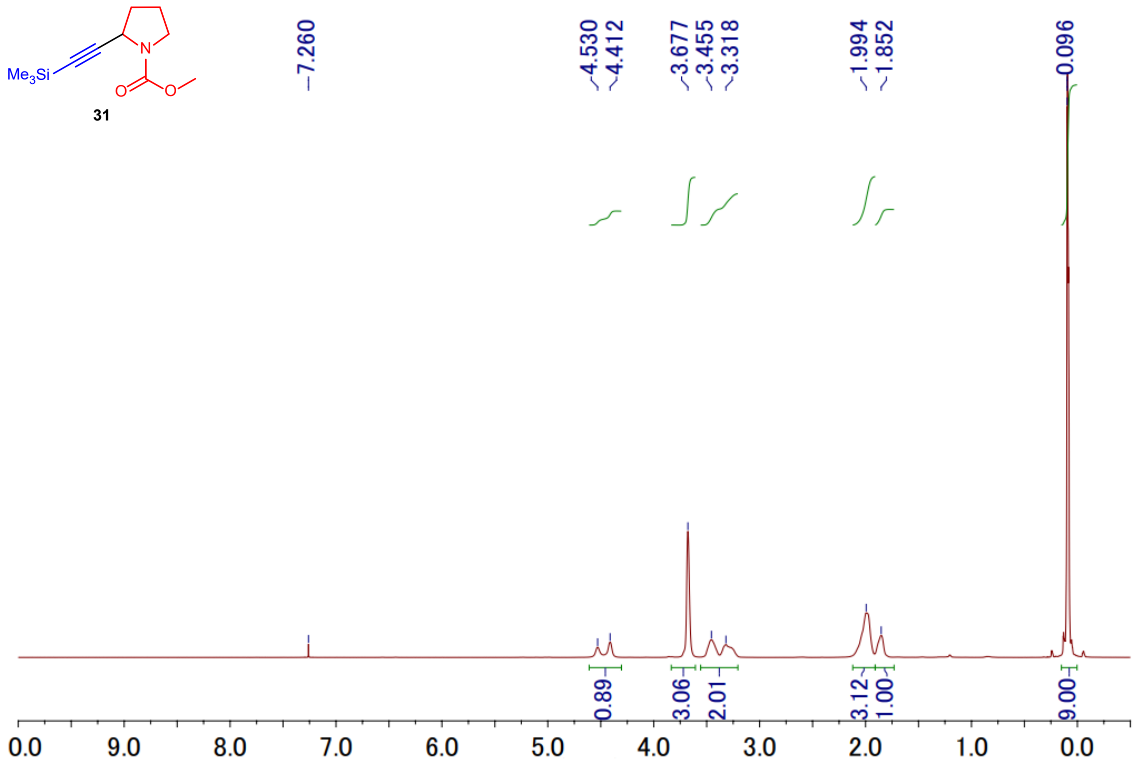

^{1}H NMR spectrum of methyl 2-(trimethylsilylethynyl)pyrrolidine-1-carboxylate (31) (400 MHz, $CDCl_3$)

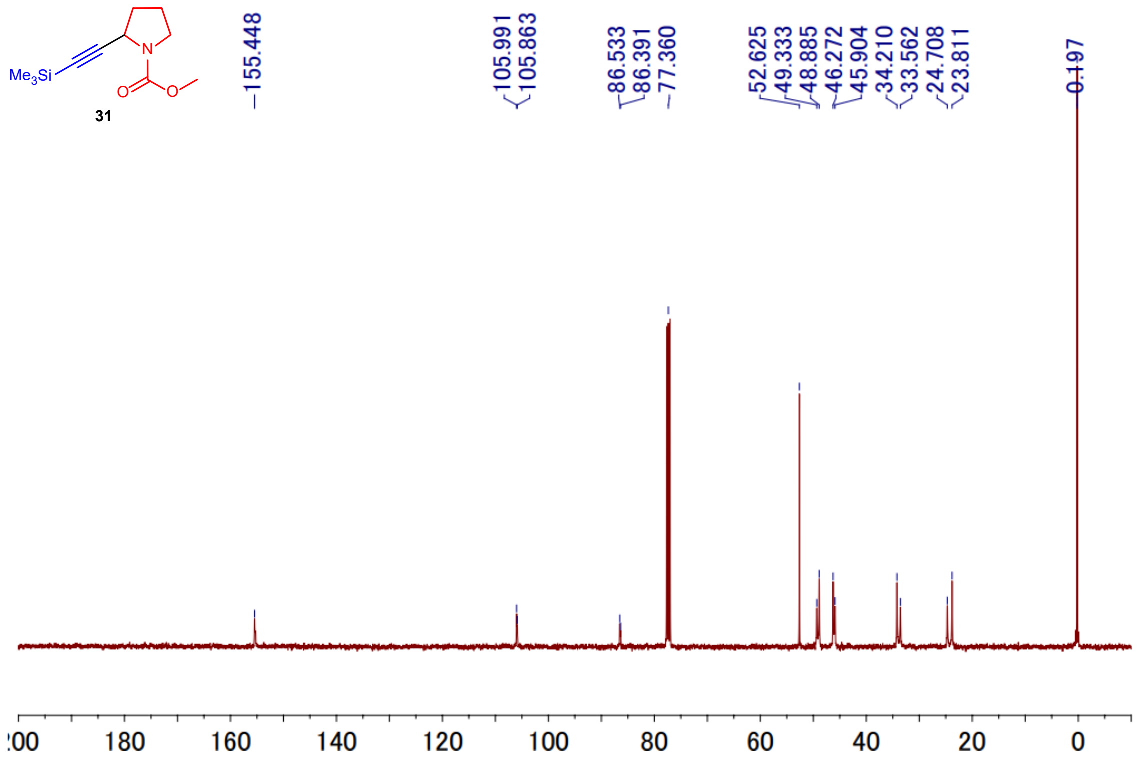

^{13}C NMR spectrum of methyl 2-(trimethylsilylethynyl)pyrrolidine-1-carboxylate (31) (100 MHz, $CDCl_3$)

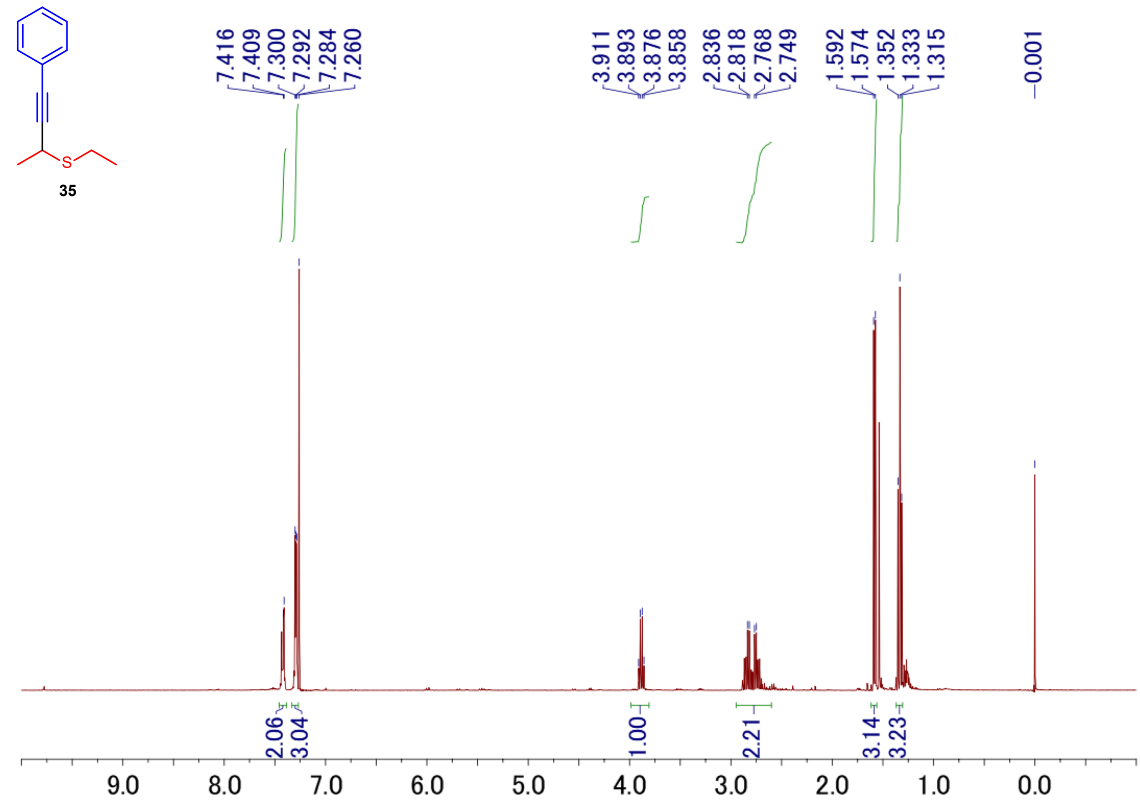

^{1}H NMR spectrum of ethyl 4-phenyl-3-butyn-2-yl sulfide (35) (400 MHz, $CDCl_3$)

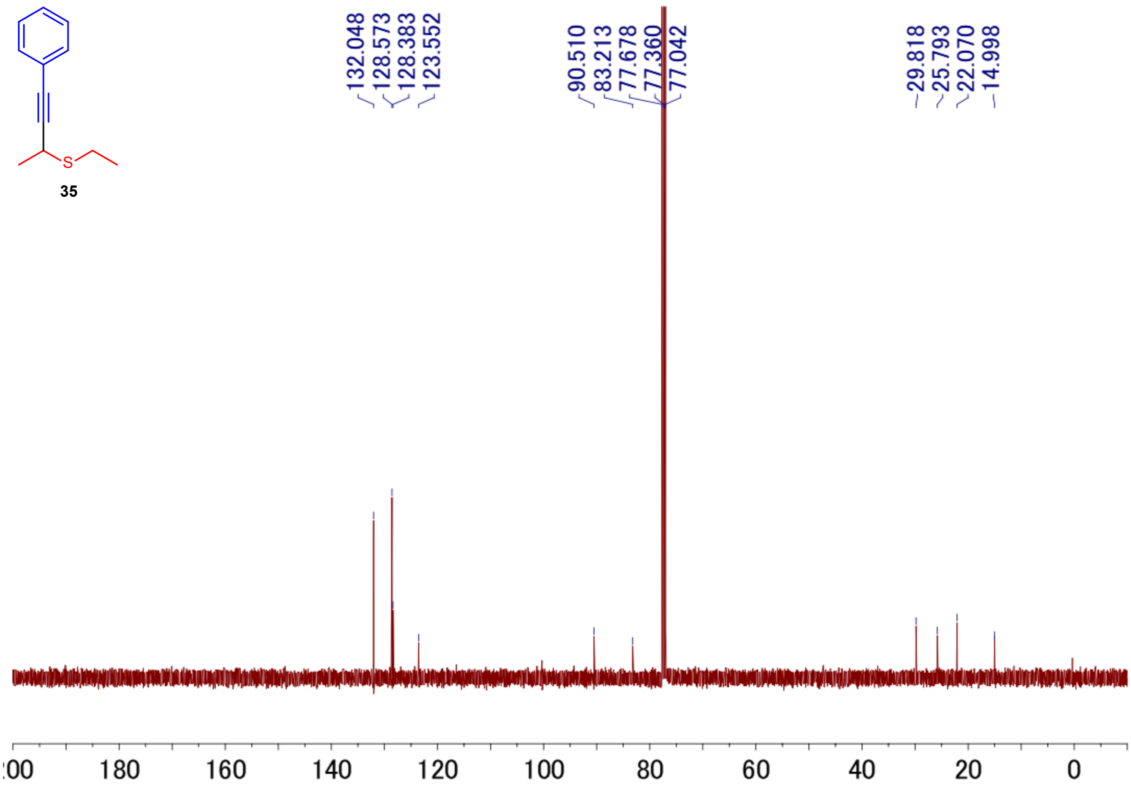

^{13}C NMR spectrum of ethyl 4-phenyl-3-butyn-2-yl sulfide (35) (100 MHz, $CDCl_3$)

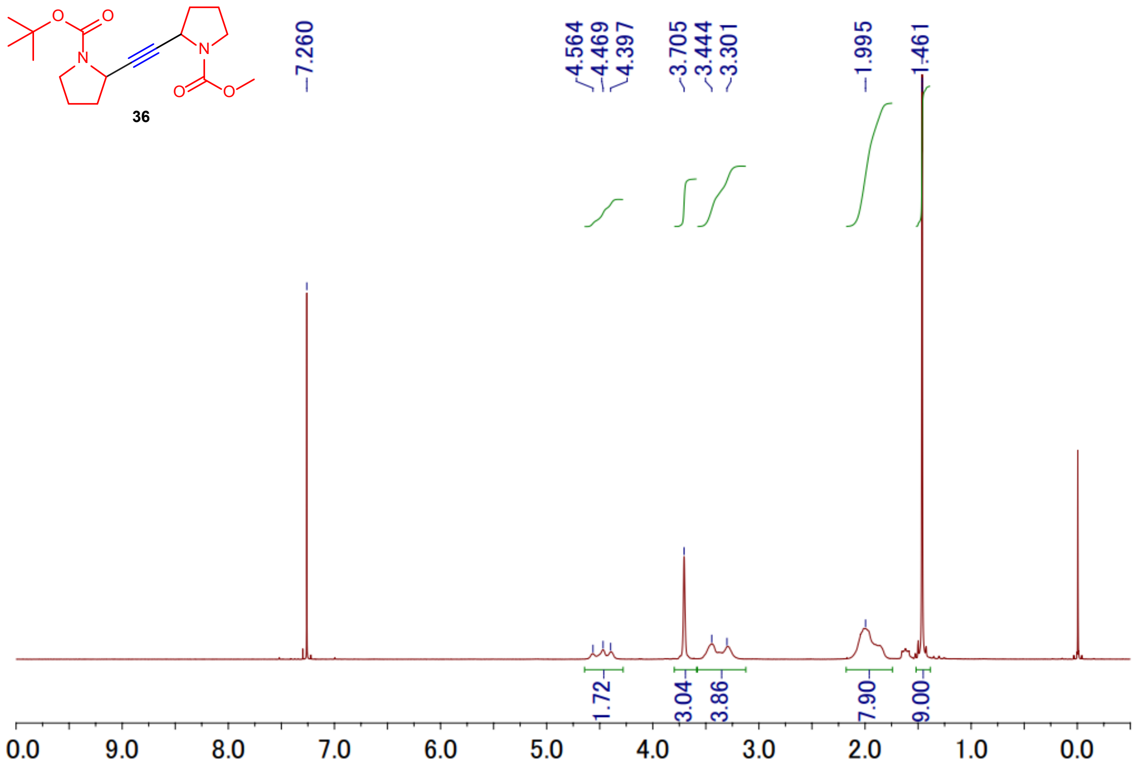

^{1}H NMR spectrum of *tert*-butyl 2-((1-(methoxycarbonyl)pyrrolidin-2-yl)ethynyl)pyrrolidine-1-carboxylate (36) (400 MHz, $CDCl_3$)

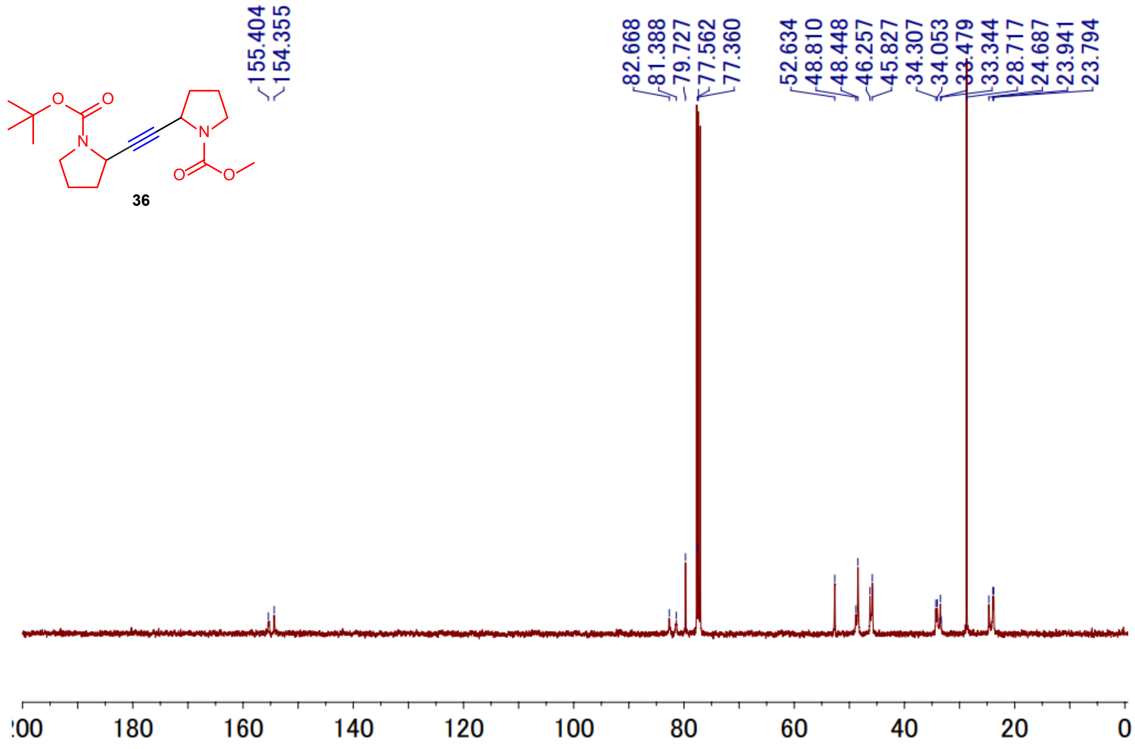

^{13}C NMR spectrum of *tert*-butyl 2-((1-(methoxycarbonyl)pyrrolidin-2-yl)ethynyl)pyrrolidine-1-carboxylate (36) (100 MHz, $CDCl_3$)

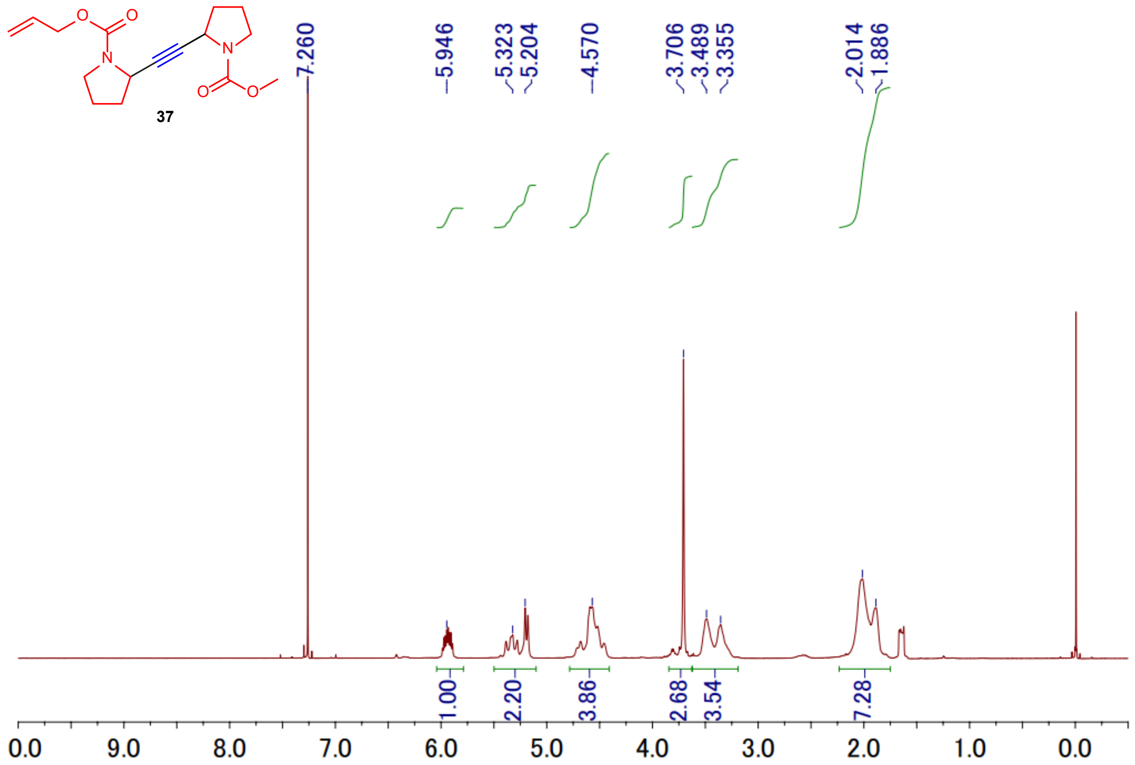

^{1}H NMR spectrum of allyl 2-((1-(methoxycarbonyl)pyrrolidin-2-yl)ethynyl)pyrrolidine-1-carboxylate (37) (400 MHz, $CDCl_3$)

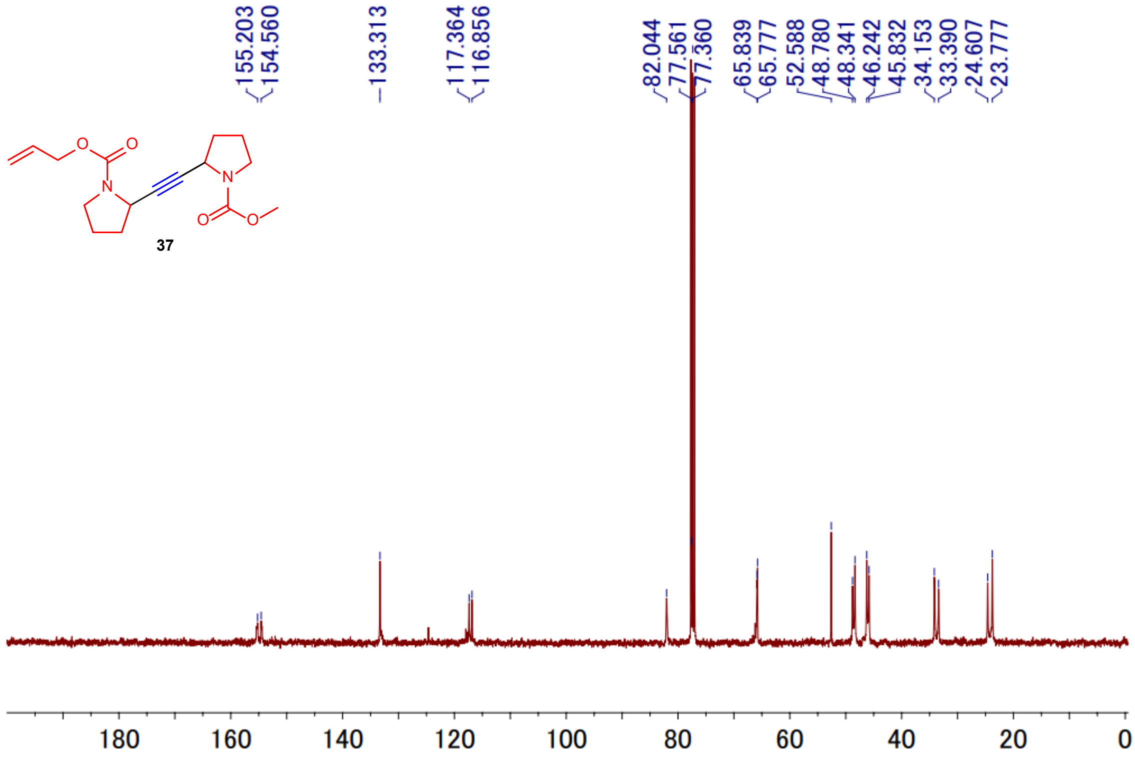

^{13}C NMR spectrum of allyl 2-((1-(methoxycarbonyl)pyrrolidin-2-yl)ethynyl)pyrrolidine-1-carboxylate (37) (100 MHz, $CDCl_3$)

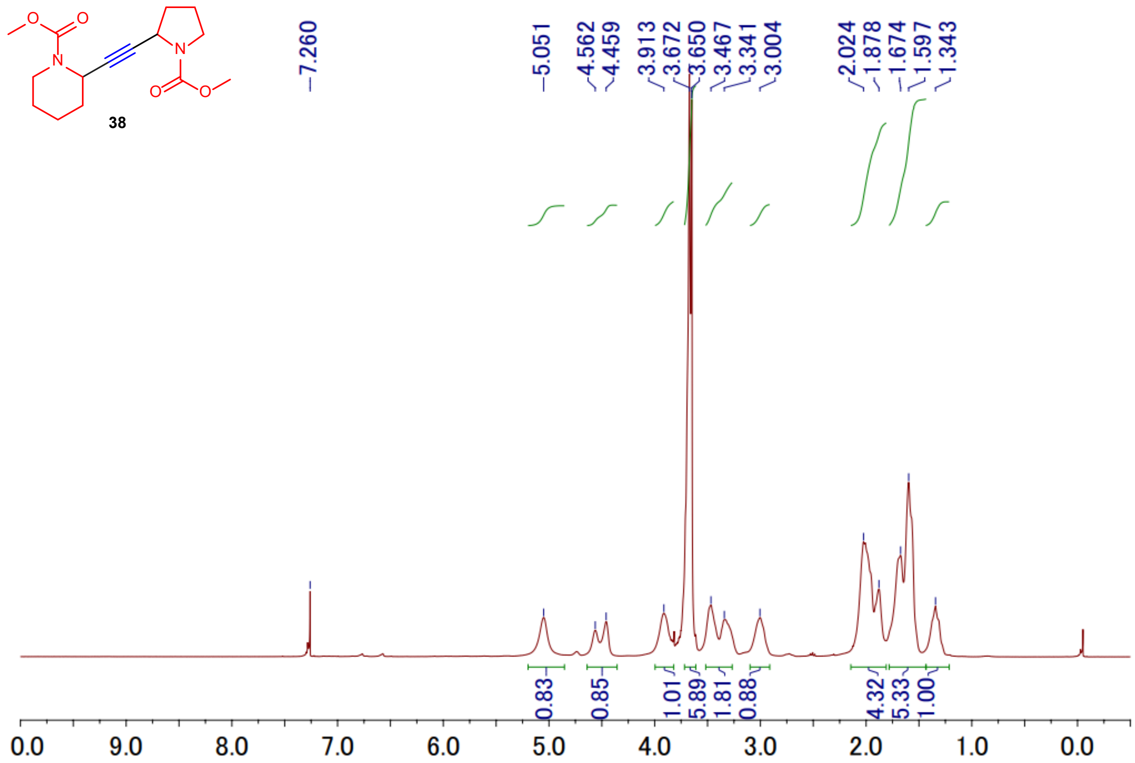

^{1}H NMR methyl 2-((1-(methoxycarbonyl)pyrrolidin-2-yl)ethynyl)piperidine-1-carboxylate (38) (400 MHz, $CDCl_3$)

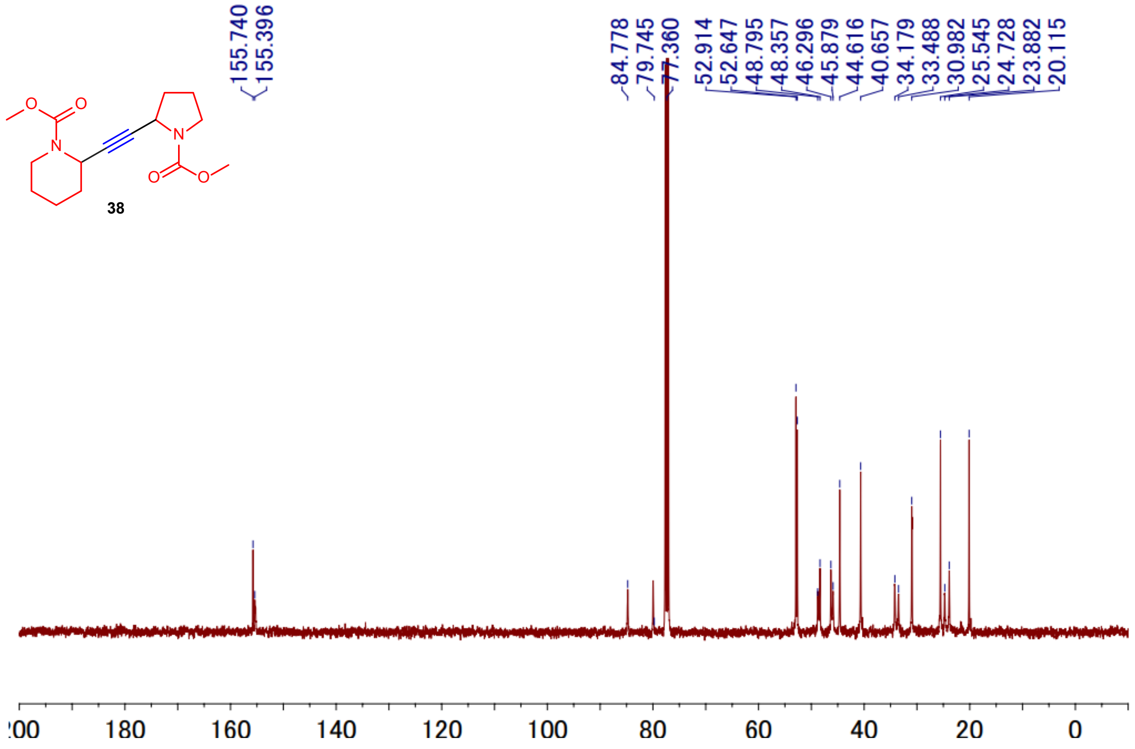

^{13}C NMR methyl 2-((1-(methoxycarbonyl)pyrrolidin-2-yl)ethynyl)piperidine-1-carboxylate (38) (100 MHz, $CDCl_3$)

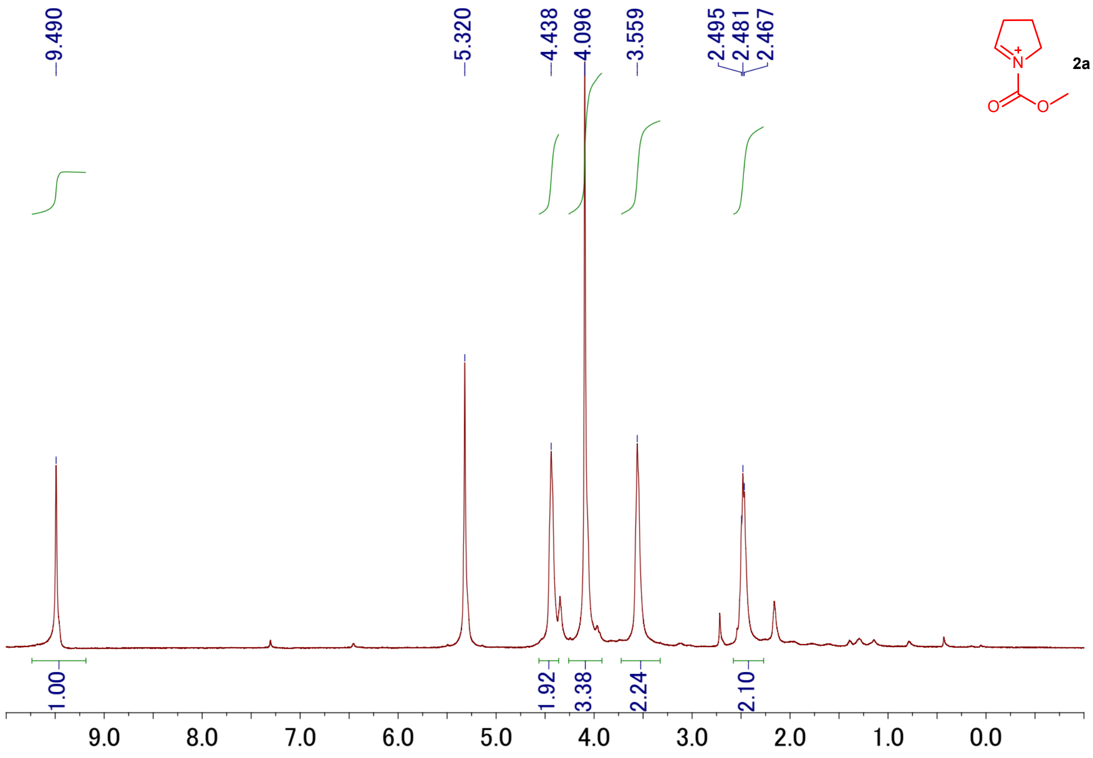

^{1}H NMR spectrum of *N*-acyliminium ion 2a (500 MHz, CD_2Cl_2, −78 °C).

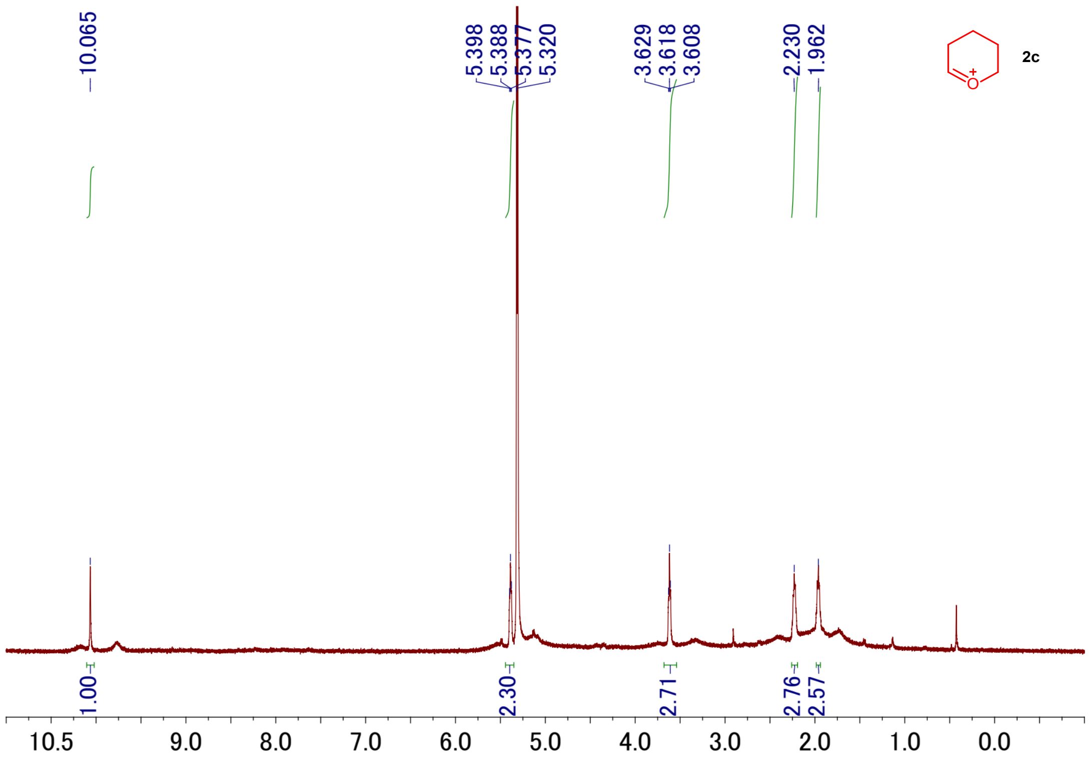

^{1}H NMR spectrum of oxonium ion 2c (500 MHz, CD_2Cl_2, −78 °C).

4. References

1. Mikami, R.; Nakamura, Y.; Shida, N.; Atobe, M. *React. Chem. Eng.* **2021**, *6*, 2024.
2. Merx, J.; Houthuijs, K. J.; Elferink, H.; Witlox, E.; Mecinović, J.; Oomens, J.; Martens, J.; Boltje T. J.; Rutjes, F. P. J. T. *Chem. Eur. J.* **2021**, *28*, e202104078.
3. Li, G.; Kates, P. A.; Dilger, A. K.; Cheng, P. T.; Ewing, W. R.; Groves, J. T. *ACS Catal.* **2019**, *9*, 9513.
4. Aguilar, N.; Fernandez, J. C.; Terricabras, E.; Carceller, G. E.; Garcia, F. J.; Salas, S. J. PCT WO2013149996.
5. Yan, J.; Zhang, W.; He, Q.; Hou, J.; Zeng, H.; Wei, H.; Xie, W. *Org. Biomol. Chem*. **2022**, *20*, 2387.
6. Chen, C.; Kattanguru, P.; Tomashenko, O. A.; Karpowicz, R.; Siemiaszko, G.; Bhattacharya, A.; Calasans, V.; Six, Y. *Org. Biomol. Chem*. **2017**, *15*, 5364.
7. Leroux, M.; Vorherr, T.; Lewis, I.; Schaefer, M.; Koch, G.; Karaghiosoff, K.; Knochel, P. *Angew. Chem. Int. Ed.* **2019**, *58*, 8231.
8. Krasovskiy, A.; Knochel, P. *Synthesis* **2006**, 890.
9. lv, P.; Zhang, L.; Srinivasakannan, C.; Li, S.; He, Y.; Chen K.; Yin, S. *Macrochem. J.* **2020**, *155*, 104662.
10. Commenge, J.-M.; Falk, L. *Chem. Eng. Process.; Process Intensif.* **2011**, *50*¸ 979.
11. Phillips, T. W.; Murphy, K. P. *J. Chem. Eng. Data* **1970**, *15*, 304.
12. Asano, S.; Yatabe, S.; Maki, T.; Mae, K. *Org. Process Res. Dev.* **2019**, *23*, 807.
13. Yoshida, J.; Suga, S.; Suzuki, S.; Kinomura, N.; Yamamoto, A.; Fujiwara, K. *J. Am. Chem. Soc.* **1999**, *121*, 9546.
14. Grossmann, O.; Maji, R.; Aukland, M. H.; Lee, S.; List, B. *Angew. Chem. Int. Ed.* **2022**, *61*, e202115036.
15. Sun, Z.; Kumagai, N.; Shibasaki, M. *Org. Lett.* **2017**, *19*, 3727.
16. Okajima, M. *New Developments in "Cation Pool" Method and Their Applications to Microflow Systems*, PhD Dissertation, Kyoto University, Japan, **2005**.
17. Wang, F.; Rafiee, M.; Stahl, S. S. *Angew. Chem. Int. Ed.* **2018**, *130*, 6796.
18. You, T.; Zeng, S.-H. Fan, J.; Wu, L.; Kang, F.; Liu, Y.; Che, C.-M. *Chem. Commun.* **2021**, *57*, 10711.
19. Im, H.; Kang, D.; Choi, S.; Shin, S.; Hong, S. *Org. Lett.* **2018**, *20*, 7437.
20. Wan, M.; Meng, Z.; Lou, H.; Liu, L. *Angew. Chem. Int. Ed.* **2014**, *53*, 13845.
21. Hoshikawa, T.; Kamijo, S.; Inoue, M. *Org. Biomol. Chem.* **2013**, *11*, 164.
22. Ying, W.; Herndon, J. W. *Eur. J. Org. Chem.* **2013**, 3112.
